# Exercise and nutrition strategies for sarcopenia in older adults: evidence from a network meta-analysis based on EWGSOP and AWGS criteria

**DOI:** 10.3389/fnut.2025.1685014

**Published:** 2025-10-16

**Authors:** Rongting Zhao, Yangjian Dong, Quansheng Zheng, Jiwei Yao

**Affiliations:** ^1^College of Physical Education and Health, Guangxi Normal University, Guilin, Guangxi, China; ^2^College of Physical Education, China Three Gorges University, Yichang, Hubei, China; ^3^College of General Education, Guizhou University of Commerce, Guiyang, China

**Keywords:** sarcopenia, exercise, nutrition, older adults, resistance training

## Abstract

**Objectives:**

This network meta-analysis aimed to evaluate the comparative effectiveness of different exercise and nutritional interventions on muscle strength, skeletal muscle mass, and physical function in older adults with sarcopenia diagnosed according to the European Working Group on Sarcopenia in Older People and Asian Working Group for Sarcopenia criteria.

**Methods:**

We systematically searched PubMed, Embase, Web of Science, and the Cochrane Central Register of Controlled Trials up to July 2025. A Bayesian random-effects network meta-analysis was performed, with additional subgroup and meta-regression analyses. Certainty of evidence was assessed using the Confidence in Network Meta-Analysis (CINeMA) framework, and interventions were ranked according to their relative effectiveness and certainty of evidence. This study was registered in PROSPERO (CRD420251124534).

**Results:**

A total of 35 randomised controlled trials involving 2,331 participants were included. Exercise combined with nutritional supplementation was the most effective intervention for improving handgrip strength (MD = 3.69, 95% CrI 0.72 to 5.10; SUCRA 99.04%), gait speed (MD = 0.11, 95% CrI 0.03 to 0.17; SUCRA 87.12%), and appendicular skeletal muscle mass index (ASMI) (MD = 0.35, 95% CrI 0.19 to 0.49; SUCRA 99.82%), with improvements in handgrip strength and ASMI significantly greater than those achieved with Exercise or Nutrition alone. Exercise alone improved handgrip strength, gait speed, and ASMI, whereas protein supplementation alone improved handgrip strength and gait speed but had no significant effect on ASMI. Subgroup analyses indicated that resistance training with protein supplementation produced the most significant improvements in handgrip strength and gait speed. In contrast, resistance training with protein and vitamin D supplementation was most effective for improving ASMI. Meta-regression analysis did not identify any significant sources of heterogeneity.

**Conclusion:**

These findings support combined exercise and nutritional interventions as a preferred treatment option for improving muscle strength, muscle mass, and physical function in older adults with sarcopenia. However, the overall certainty of the evidence ranged from low to very low. In particular, multicomponent exercise programmes centred on resistance training and combined with protein supplementation may offer superior benefits for enhancing muscle strength and physical function.

## 1 Introduction

Sarcopenia is an age-related condition characterised by loss of muscle mass, decline in muscle strength, and deterioration in physical function (1, 2). It is associated with an increased risk of adverse clinical outcomes, including disability, falls, and mortality (1, 2). It is common among older adults, particularly those aged 65 years and above. Depending on the diagnostic criteria and population background, prevalence estimates vary widely from 10 to 50%, and even the most conservative estimates suggest that 5–10% of the general population is affected (3, 4). This burden represents a persistent and substantial challenge for public health systems and the allocation of healthcare resources (5).

Currently, no pharmacological therapy has been proven in clinical practice to have definitive efficacy against sarcopenia (6). International guidelines consistently recommend non-pharmacological interventions as the preferred approach, with particular emphasis on the role of modifiable factors such as exercise and nutrition in prevention and reversal (2, 7). These strategies are considered feasible to implement and carry significant public health relevance (6). Among them, resistance training is recognised as one of the most effective interventions for increasing muscle strength and mass and is supported by high-quality evidence (2, 5, 7). Its effects are primarily mediated through mechanical loading that activates the mTORC1 signalling pathway, thereby enhancing muscle protein synthesis, while also improving motor unit recruitment and neuromuscular coordination to promote gains in strength and function (8). However, because current resistance training guidelines are primarily based on machine- or free-weight exercises, standardized programmes may lack key components that facilitate functional transfer, and the resulting strength gains may not fully translate into functional improvements (9). In fact, functional recovery requires not only strength but also neuromuscular control, balance, and coordination, and therefore resistance training alone is often insufficient to provide comprehensive benefits for individuals with functional limitations (10). For individuals with functional limitations, resistance training alone may not provide comprehensive benefits (11). In clinical settings, resistance training is frequently incorporated into multicomponent programmes that include aerobic and balance training to facilitate the translation of strength gains into functional improvements (10). In parallel, Protein supplementation is a key nutritional strategy to enhance muscle protein synthesis after exercise and is recommended in international consensus statements as an important adjunctive therapy (7). Leucine-rich proteins can directly stimulate mTORC1 signalling and provide the substrates required for protein accretion, thereby amplifying the post-exercise anabolic response and partially overcoming age-related anabolic resistance (12). Although protein supplementation is often combined with exercise in clinical practice to reinforce training effects, the proposed synergistic effect remains debated. It has not yet been confirmed by high-quality evidence (5, 7, 13).

In addition, with the growing diversity of exercise and nutritional interventions, and their various combinations, it remains unclear which strategies provide the greatest clinical benefits for sarcopenia, particularly in terms of skeletal muscle mass, muscle strength, and physical performance. However, direct head-to-head comparisons between different interventions are scarce, and previous reviews have often been unable to determine the relative effectiveness of exercise, nutrition, or their combination. A further major challenge lies in the lack of universally accepted diagnostic criteria, with many primary studies using inconsistent or even non-standard definitions. This has led to systematic differences in participants’ functional status and disease severity across studies, thereby undermining the interpretability and generalisability of the findings (12, 13). As a result, this methodological heterogeneity has also constrained the conclusions of earlier meta-analyses, which often pooled trials with disparate definitions and thus provided evidence of limited clinical applicability.

To address this limitation, the present study employed a network meta-analysis to compare and synthesise the available interventions systematically. However, in network meta-analyses, ensuring comparability of disease severity and functional status across study populations is a prerequisite for minimising between-study heterogeneity and ensuring the reliability of intervention rankings (14). To address this methodological challenge and strengthen clinical applicability, the present study included only randomised controlled trials that applied the most internationally recognised and clinically relevant consensus definitions for sarcopenia, namely the EWGSOP and AWGS criteria (15, 16). Within this standardised diagnostic framework, we synthesised the best available evidence to compare and rank the relative effects of different exercise and nutritional interventions on skeletal muscle mass, muscle strength, and physical performance in older adults with sarcopenia. We aimed to generate robust evidence-based recommendations to guide clinical practice.

## 2 Methods

### 2.1 Protocol and registration

The protocol for this systematic review and network meta-analysis was registered in PROSPERO (CRD420251124534). The study followed the PRISMA 2020 guidelines for systematic reviews and meta-analyses and the PRISMA-NMA extension for network meta-analyses (17, 18).

### 2.2 Search strategy and study selection

We searched PubMed, Web of Science, the Cochrane Central Register of Controlled Trials (CENTRAL), and Embase for randomised controlled trials (RCTs) published from database inception to July 2025. The search strategy combined Medical Subject Headings (MeSH) and free-text keywords, with Boolean operators (AND, OR) applied to ensure comprehensive coverage. An example of a core search string was: Sarcopenia[MeSH Terms] AND (Resistance training[MeSH Terms] OR Whey Proteins[MeSH Terms] OR beta-hydroxyisovaleric acid[MeSH Terms] OR Amino Acids, Essential[MeSH Terms] OR Leucine[MeSH Terms] OR Amino Acids, Branched-Chain[MeSH Terms] OR Amino Acids[MeSH Terms]). The complete search strategies for each database are provided in Appendix 1. For study selection, three reviewers (GS, BW, and LX) independently screened titles, abstracts, and full texts according to predefined eligibility criteria. Disagreements were resolved through consultation with a fourth reviewer (YE). To minimise the risk of missing relevant studies, we also screened the reference lists of included articles and related systematic reviews.

### 2.3 Eligibility criteria

Eligibility was assessed using the PICOS framework (Participants, Interventions, Comparators, Outcomes, and Study design) (19). Studies were included if they met all of the following criteria: (1) Participants were adults aged ≥65 years with sarcopenia diagnosed according to consensus definitions from either the European Working Group on Sarcopenia in Older People (EWGSOP) or the Asian Working Group for Sarcopenia (AWGS). (2) Interventions consisted of structured exercise programmes centred on resistance training, protein-based supplementation, or a combination of the two. (3) Control groups received health education, usual care, or placebo. (4) At least one of the following primary outcomes was reported: handgrip strength, gait speed, or appendicular skeletal muscle mass index (ASMI). These outcomes are widely recommended in international consensus statements as core measures of muscle strength, physical performance, and skeletal muscle mass (2). (5) Study design was restricted to RCTs.

Exclusion criteria were: (1) Participants with sarcopenia secondary to specific health conditions such as cancer, diabetes, stroke, HIV, chronic obstructive pulmonary disease, chronic kidney disease, liver cirrhosis, other severe illnesses, or recent organ transplantation. (2) Studies published in languages other than English. (3) Studies without sufficient data for analysis. (4) Full-text articles unavailable from databases or other sources.

### 2.4 Data extraction

Data were extracted independently by two reviewers (RT and YJ) using a predesigned standardized Excel spreadsheet (Microsoft Excel, 2019; Microsoft Corporation, Redmond, WA, USA) and verified by a third reviewer (JW). Extracted data included: study characteristics (first author, year, country, diagnostic criteria), population characteristics (age, sample size), intervention characteristics (duration, type of exercise, nutritional supplementation details), and outcome data (mean and standard deviation for all continuous variables). Where data were missing, up to three email requests were sent to corresponding authors at three-week intervals.

### 2.5 Measures of treatment effect

Treatment effects were expressed as mean differences (MD) with standard deviations (SD). When SDs were not reported, they were calculated from standard errors, 95% confidence intervals, *p* values, or t statistics (20). For studies lacking SDs of change scores, we estimated these using the following formula:


$${\mathrm{SD}}_{\text{change}}=\sqrt{{\mathrm{SD}}_{\text{baseline}}^{2}+{\mathrm{SD}}_{\text{Post}}^{2}-2\times r\times {\mathrm{SD}}_{\text{baseline}}\times {\mathrm{SD}}_{\text{post}}}$$

Where the correlation coefficient (r) was set at 0.5, reflecting a moderate level of test–retest reliability commonly accepted in the literature (20). This assumption balances potential variability between pre- and post-intervention measurements to enhance robustness.

### 2.6 Quality assessment of evidence

The risk of bias for each included trial was assessed using the Cochrane Risk of Bias tool for randomised trials (ROB 2.0), covering random sequence generation, allocation concealment, blinding, incomplete outcome data, and selective outcome reporting (21). A study was considered to have an overall low risk of bias if all domains were rated as low risk (score 1). If at least one domain was rated as high risk, the overall risk of bias was considered high (score 3). In all other cases, the risk of bias was considered to raise some concerns (score 2). Two reviewers independently performed the assessments, and disagreements were resolved through discussion.

The certainty of evidence was evaluated using the CINeMA framework, considering six domains: within-study bias, reporting bias, indirectness, imprecision, heterogeneity, and incoherence (22, 23). These domains address potential systematic errors within individual studies, the impact of selective reporting and publication bias, the relevance of evidence to the research question, the degree of uncertainty in effect estimates, consistency across study results, and the agreement between direct and indirect evidence.

### 2.7 Minimally contextualised framework

A minimally contextualised framework was applied to grade imprecision, using the control group as the reference and classifying interventions according to the magnitude of effect and certainty of evidence (24). An effect size of zero was taken as the threshold for no effect. Interventions were categorised as among the most effective if the credible interval did not include zero. The point estimate was clearly away from the null, and intermediate if the point estimate was away from the null. Still, the credible interval was close to or overlapped zero, and among the least effective if the credible interval included zero and the point estimate was close to zero (25, 26). Certainty of evidence was further classified as high or moderate versus low or very low based on the GRADE framework to guide interpretation (24).

To assess clinical relevance, we referred to previously established minimal important differences (MID) for key sarcopenia outcomes: 5.0 kg for handgrip strength (27) and 0.10 m/s for gait speed (28).

### 2.8 Statistical analysis

The network meta-analysis was conducted in R (version 4.3.3; R Foundation for Statistical Computing, Vienna, Austria) using the multinma package within a Bayesian framework. The treatment network was depicted with nodes representing interventions and edges representing head-to-head comparisons. Treatment effects were estimated using MCMC methods, with random-effects models fitted to account for between-study heterogeneity (29, 30). MD with 95% credible intervals (CrIs) were used as the measure of effect for all outcomes, applying consistent units or scales across studies. Heterogeneity was quantified using τ^2^, interpreted as low (<0.04), low to moderate (0.04–0.16), moderate to high (0.16–0.36), or high (>0.36) (31, 32). Global inconsistency was assessed by comparing model fit between the consistency model and the unrelated mean effects model, using residual deviance, deviance information criterion (DIC), and the effective number of parameters (pD).^33 Local inconsistency was examined using the node-splitting method, which compares direct and indirect evidence; a *p*-value below 0.05 was considered to indicate significant inconsistency (33).

To rank interventions, the surface under the cumulative ranking curve (SUCRA) was calculated using the MetaInsight tool (version 6.4.0; University of Leicester, Leicester, UK). SUCRA values quantify the overall probability of each intervention being the best option, enabling the identification of the most effective approach (34). Meta-regression analyses were conducted to explore potential effect modifiers, including age, proportion of male participants, sample size, intervention duration, baseline BMI, baseline handgrip strength, and baseline ASMI, and to test the robustness of the results. Publication bias was assessed by visual inspection of funnel plots generated using the netmeta package and further evaluated with Egger’s test.

## 3 Results

### 3.1 Literature selection and study characteristics

**Figure 1:**
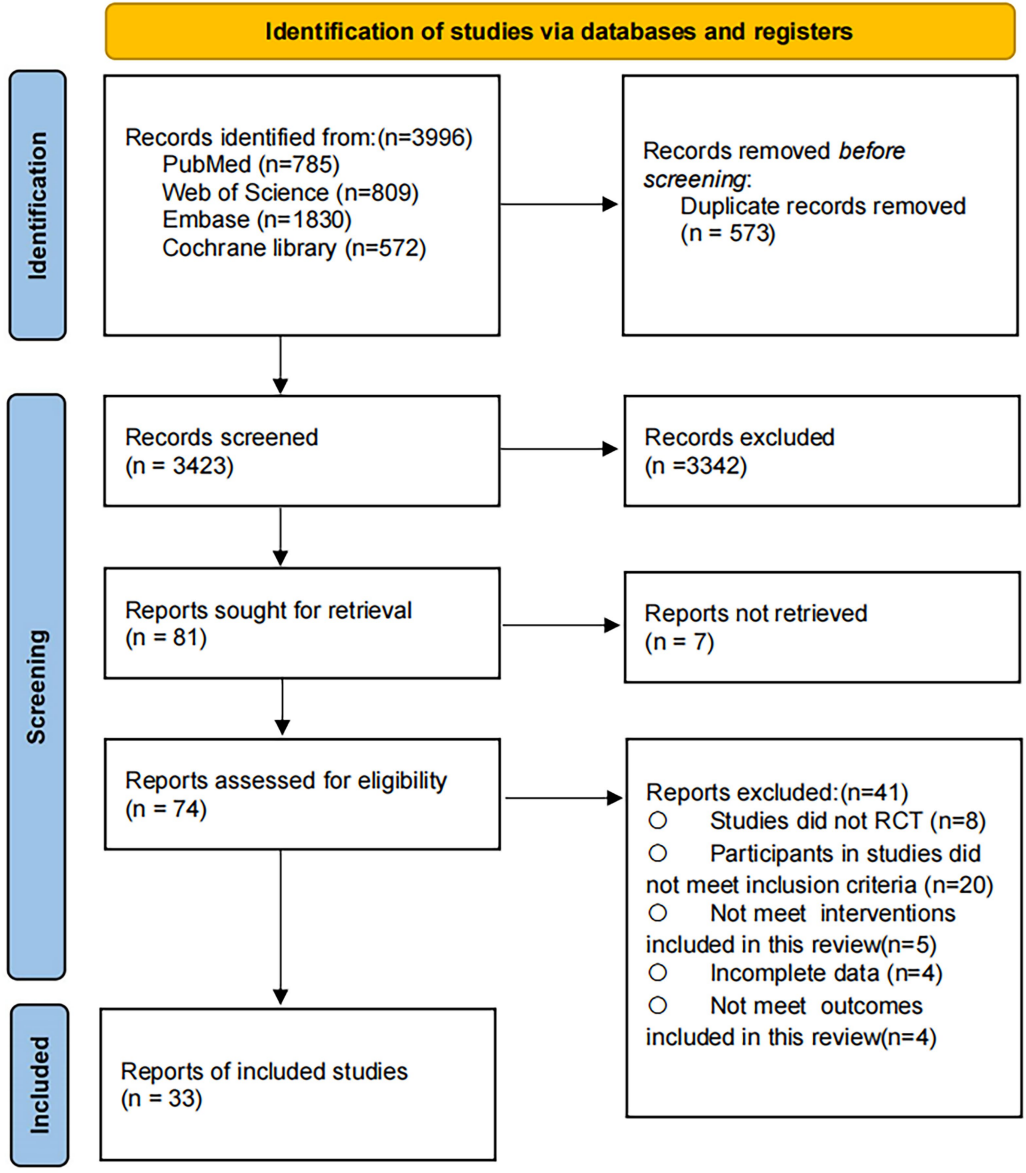
PRISMA flow diagram of the search process for studies.

The systematic search identified 3,996 potential records. After removal of duplicates, 3,423 articles remained for title and abstract screening. Of these, 74 articles were retrieved for full-text review. In total, 35 studies met the inclusion criteria and were included in the systematic review and meta-analysis, comprising 2,331 participants with a mean age of 74.95 (SD 5.58) years. The whole selection process is presented in Figure 1, detailed characteristics of included studies are provided in Appendix 2, and the complete search strategies are reported in Appendix 1.

### 3.2 Risk of bias and certainty of evidence

**Figure 2:**
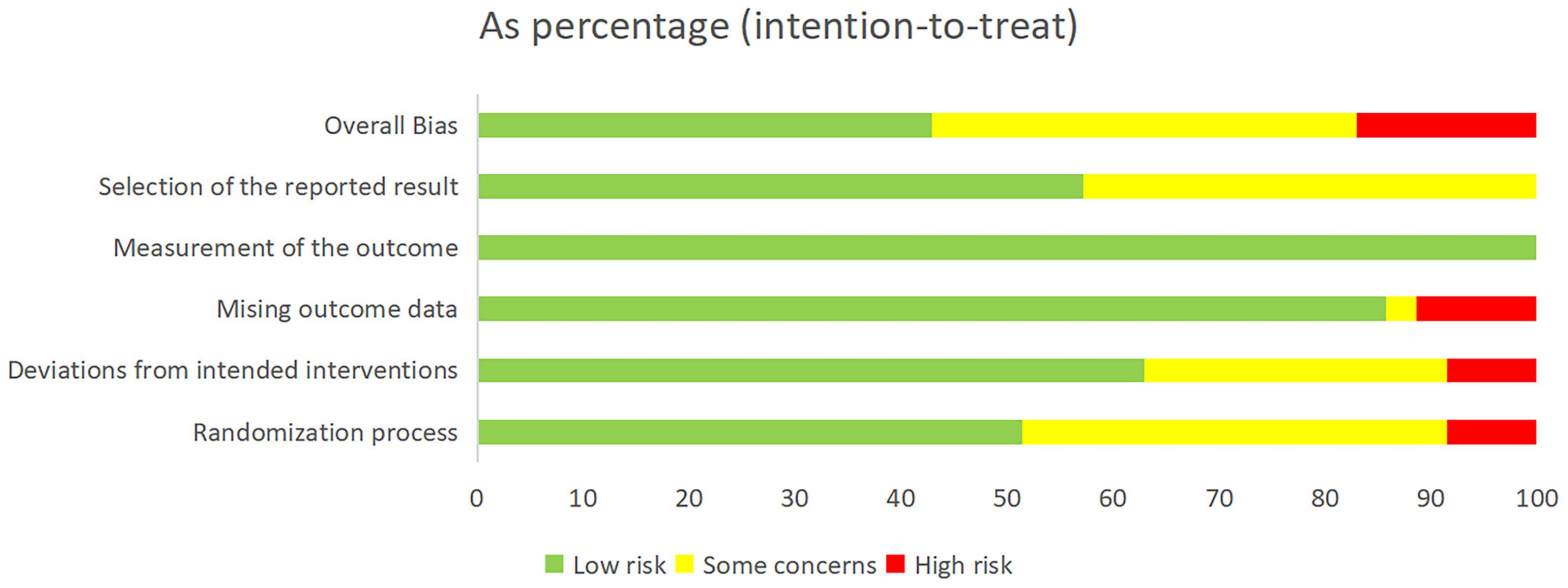
Overall risk of bias is presented as a percentage of each risk of bias item across all included studies.

Overall, 15 studies (42.9%) were judged to be at low risk of bias, 14 (40%) at some concerns, and 6 (17.1%) at high risk of bias (Figure 2). Risk of bias assessments for each trial are provided in Appendix 3. To evaluate global consistency within the network, the goodness-of-fit of the consistency and inconsistency models was compared. Across all outcomes, the difference in the deviance information criterion (DIC) between the two models was less than 5, indicating comparable fit and no statistically significant evidence of global inconsistency (Appendix 4). In local consistency assessment, the node-splitting approach identified discrepancies between direct and indirect evidence for specific comparisons, leading to downgrading of the certainty for those specific outcomes (Appendix 5). Using the CINeMA framework, the certainty of evidence for all pairwise comparisons was rated, with most classified as “very low” to “low” (Appendix 8). The results of the minimally contextualised framework are summarised in Table 1. Funnel plot analysis did not reveal evidence of asymmetry, suggesting no substantial publication bias (Appendix 9).

**Table 1:** The results of the minimally contextualised framework.

Outcome	Certainty of evidence	Group	Intervention	Intervention vs Control	SUCRA
Handgrip strength	Low certainty (low to very low certainty evidence)	Category 2: among the most effective	Exercise + Nutrition	3.69 (0.72; 5.10)	99.04
		Category 1: intermediately effective	Exercise	2.21 (0.6; 3.37)	54.85
			Nutrition	1.95 (0.69; 3.32)	45.95
Gait speed	Low certainty (low to very low certainty evidence)	Category 2: among the most effective	Exercise + Nutrition	0.11 (0.03; 0.17)	87.12
		Category 1: intermediately effective	Nutrition	0.08 (0.03; 0.14)	60.33
			Exercise	0.07 (0.02; 0.12)	52.34
Appendicular skeletal muscle mass index	Low certainty (low to very low certainty evidence)	Category 2: among the most effective	Exercise + Nutrition	0.35 (0.19; 0.49)	99.82
		Category 1: intermediately effective	Exercise	0.16 (0.03; 0.28)	54.06
		Category 0: among the least effective	Nutrition	0.11 (−0.02; 0.23)	44.66

### 3.3 Muscle strength

**Figure 3:**
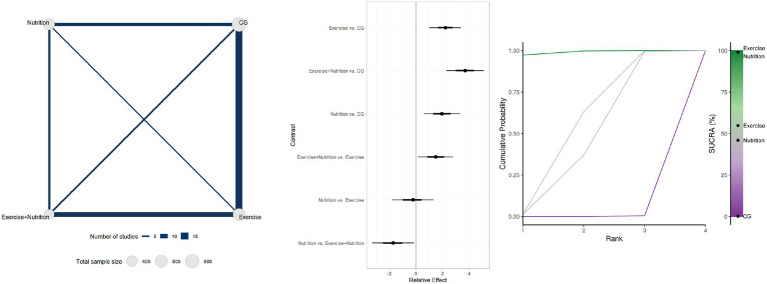
Summary of network meta-analysis results for handgrip strength: evidence network, relative effects, and SUCRA ranking.

Thirty studies involving 1954 participants reported changes in handgrip strength (Figure 3). Low- or very low-certainty evidence indicated that Exercise + Nutrition (MD = 3.69, 95% CI 0.72 to 5.10; SUCRA 99.04%), exercise alone (MD = 2.21, 95% CI 0.60 to 3.37; SUCRA 54.85%), and nutrition alone (MD = 1.95, 95% CI 0.69 to 3.32; SUCRA 45.95%) all significantly improved handgrip strength. However, although all three interventions reached statistical significance, none of their effect estimates reached the predefined MID threshold of 5 kg, indicating limited clinical significance. Nevertheless, further comparisons showed that Exercise + Nutrition was significantly more effective than either Exercise or Nutrition alone.

### 3.4 Physical function

**Figure 4:**
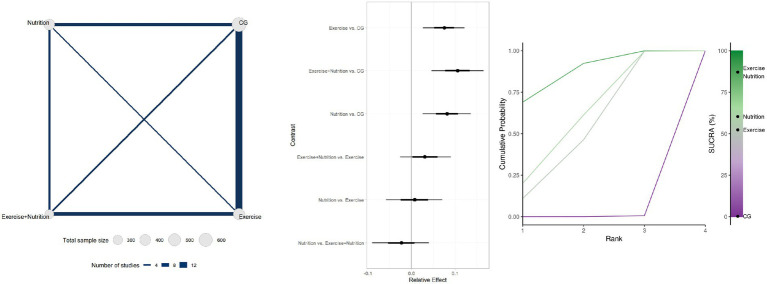
Summary of network meta-analysis results for gait speed: evidence network, relative effects, and SUCRA ranking.

Twenty-seven studies involving 1804 participants reported changes in gait speed (Figure 4). Low-certainty evidence indicated that Exercise + Nutrition (MD = 0.11, 95% CrI 0.03 to 0.17; SUCRA 87.12%), Exercise alone (MD = 0.07, 95% CrI 0.02 to 0.12; SUCRA 60.33%), and nutrition alone (MD = 0.08, 95% CrI 0.03 to 0.14; SUCRA 52.34%) all significantly improved gait speed. However, only the effect estimate for Exercise + Nutrition exceeded the predefined MID threshold of 0.10 m/s, while its 95% CrI crossed the threshold, indicating potential clinical relevance. By contrast, although Exercise alone and Nutrition alone reached statistical significance, their effect estimates did not meet the MID, indicating limited clinical significance.

### 3.5 Muscle mass

**Figure 5:**
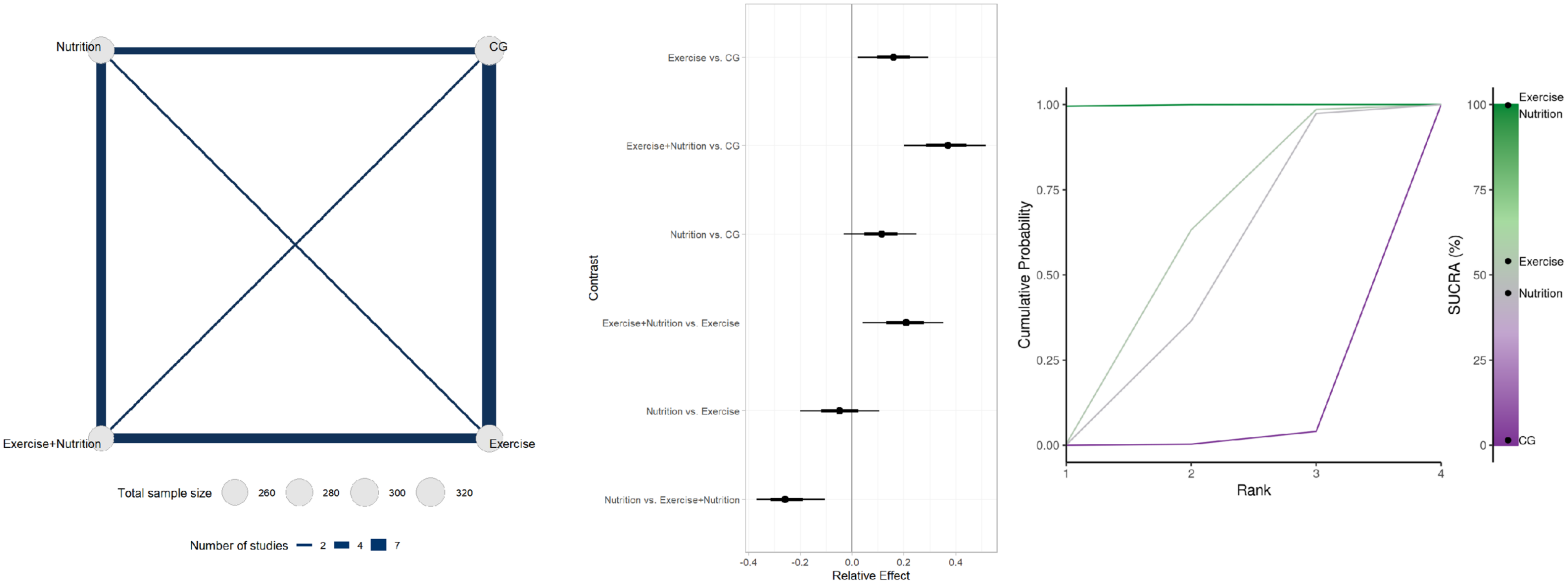
Summary of network meta-analysis results for ASMI: evidence network, relative effects, and SUCRA ranking.

Seventeen studies involving 1,137 participants reported changes in appendicular skeletal muscle mass (Figure 5). Low- or very low-certainty indicated that Exercise + Nutrition (MD = 0.35, 95% CrI 0.19 to 0.49; SUCRA 99.82%) and exercise alone (MD = 0.16, 95% CrI 0.03 to 0.28; SUCRA 54.06%) significantly improved muscle mass, whereas nutrition alone (MD = 0.11, 95% CrI − 0.02 to 0.23; SUCRA 44.66%) did not show a significant effect.

### 3.6 Subgroup analysis

**Figure 6:**
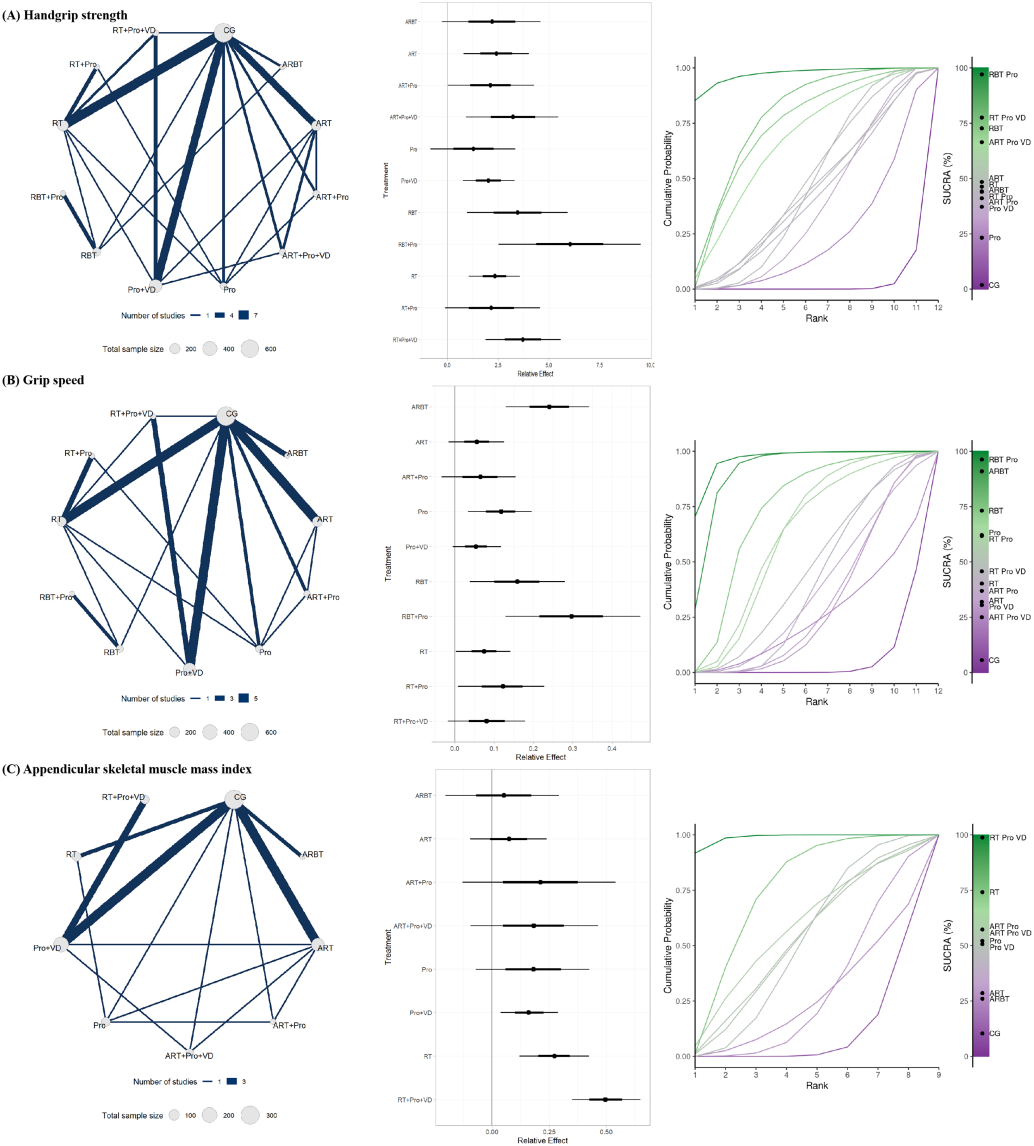
Subgroup analysis results for **(A)** handgrip strength, **(B)** gait speed, and **(C)** ASMI: evidence network, relative effects, and SUCRA rankings.

The subgroup analyses indicated that resistance and balance training plus protein supplementation (RBT + Pro), resistance training plus protein and vitamin D supplementation (RT + Pro + VD), and RBT alone were the most effective interventions for improving handgrip strength (Figure 6A). Among these, the mean effect of RBT + Pro exceeded the predefined MID threshold of 5 kg. However, the lower bound of the confidence interval did not entirely surpass this threshold, suggesting potential clinical relevance. In addition, protein plus vitamin D supplementation (Pro + VD) alone also led to significant improvements in handgrip strength, although its clinical significance remains uncertain. For gait speed, RBT + Pro, aerobic and resistance training combined (ARBT), and RBT alone were the most effective interventions (Figure 6B). Both the mean effect and lower bound of the confidence interval for RBT + Pro and ARBT exceeded the predefined MID threshold of 0.10 m/s, indicating clear clinical relevance. In contrast, although the mean effect of RBT also exceeded the MID threshold, its lower bound did not, suggesting only potential clinical relevance. Regarding ASMI, RT + Pro + VD, RT alone, and Pro + VD were the most effective interventions (Figure 6C). Notably, interventions including aerobic training (AT) did not demonstrate significant improvements in ASMI, suggesting limited benefits for muscle mass and highlighting the need for further high-quality evidence (Appendix 6).

### 3.7 Meta-regressions

To further explore the robustness of the findings, meta-regression analyses were conducted to assess potential sources of heterogeneity across all outcome measures. Covariates included mean age, proportion of male participants, sample size, intervention duration, baseline BMI, baseline handgrip strength, and baseline ASMI. No significant associations were observed between these covariates and effect sizes (*p* > 0.05), suggesting that these factors were unlikely to be major contributors to heterogeneity (Appendix 10).

### 3.8 Sensitivity analysis

Two sensitivity analyses were performed to evaluate the robustness of the network meta-analysis results. First, all potential sources of heterogeneity were adjusted to their median values, and the models were re-estimated. Second, studies at high risk of bias were excluded, and the primary outcomes were reanalysed. In both cases, results remained consistent with the primary analyses, with only minimal changes in key effect estimates and no substantive differences in effect direction or magnitude, supporting the stability of our conclusions (Appendix 11).

## 4 Discussion

To our knowledge, this is the first network meta-analysis to include only RCTs in which sarcopenia was diagnosed according to the EWGSOP or AWGS consensus criteria. This methodological approach minimises the population heterogeneity and selection bias introduced by the use of inconsistent or self-defined diagnostic criteria in previous reviews. A total of 35 RCTs involving 2,331 older adults with sarcopenia were included, allowing direct comparisons of the relative efficacy of Exercise, Nutrition, and their combination under a unified diagnostic framework. The outcomes examined were muscle mass (ASMI), muscle strength (handgrip strength), and physical function (gait speed). The results showed that Exercise, Nutrition, and combined interventions all significantly outperformed control groups in improving handgrip strength and gait speed. Although nutritional interventions alone produced significant improvements in handgrip strength and gait speed, no significant effect on ASMI was observed. By contrast, combined exercise and nutrition interventions significantly improved both handgrip strength and ASMI compared with either modality alone. To further examine differences among intervention types, we performed subgroup analyses. RBT + Pro emerged as the most effective multicomponent strategy for improving handgrip strength and gait speed, whereas RT + Pro+VD was most effective for improving ASMI. These findings provide more precise comparative evidence for the development of evidence-based prescriptions, offering directly applicable guidance for clinicians, rehabilitation professionals, and policymakers seeking to optimise intervention strategies for sarcopenia.

In our analysis, combined exercise and nutrition was the most effective strategy for improving outcomes in sarcopenia. However, meta-analyses by Wu et al. (35) and Yan et al. (13) reported no significant differences between combined exercise plus protein supplementation and either intervention alone for most key outcomes. In Yan et al.’s (13) analysis, protein supplementation alone showed no significant improvement in handgrip strength, gait speed, or ASMI. This discrepancy may relate to the inclusion of only female participants in their analysis, given that postmenopausal hormonal changes can affect protein metabolism and utilisation. Notably, Yan et al. (13) reported in subgroup analyses that RT and RT combined with nutrition were most effective for improving handgrip strength, gait speed, knee extension strength, and appendicular skeletal muscle mass, which is only partly consistent with our findings. In addition, Yang et al. (36) also reported that combined exercise and nutrition significantly improved handgrip strength, skeletal muscle mass index, gait speed, and five-times sit-to-stand performance in older adults with sarcopenia. However, these improvements did not reach clinical significance, and intervention effects appeared to vary by age, BMI, and living environment. This highlights the need for future research to pay greater attention to population differences and to explore more targeted and clinically translatable intervention strategies. Building on this, our study further provided a more refined classification of exercise and nutritional interventions. For instance, we confirmed the potential benefits of combined protein and vitamin D supplementation. Although previous systematic reviews have reported that vitamin D alone may confer minimal or no benefit for muscle strength and mass, raising serum 25-hydroxyvitamin D from deficient or insufficient levels to sufficiency may be a prerequisite for optimising the anabolic effects of protein or amino acid supplementation (37, 38). Our findings suggest that Pro + VD improved handgrip strength more than protein alone, even when combined with RT, RBT, or aerobic and resistance training (ART). Three studies similarly reported greater improvements in handgrip strength with whey protein plus vitamin D compared with whey protein alone, in both healthy older adults (39) and older adults with sarcopenia (40, 41), irrespective of RT status. However, effects on gait speed and ASMI were minimal. Taken together, current evidence suggests that vitamin D supplementation alone is unlikely to improve muscle strength or function substantially. However, when combined with protein, it may enhance muscle anabolism and strength, warranting consideration as a supportive nutritional strategy in selected populations such as patients with osteoporosis receiving antiresorptive therapy. However, supplementation should be administered gradually, and high-dose or bolus regimens should be avoided, as excessive vitamin D intake has been associated with an increased risk of falls (42, 43). Moreover, the potential risks of protein supplementation require consideration. In patients with chronic kidney disease, prolonged high protein intake may accelerate renal function decline; therefore, consumption exceeding 1.3 g/kg/day should be avoided, with individualized adjustments made under clinical supervision (44). Given that patients with chronic kidney disease are at increased risk of developing sarcopenia, protein intake should be carefully balanced to promote muscle anabolism while minimizing renal burden (45).

Existing evidence consistently supports RT as the cornerstone of sarcopenia management. However, guidelines offer limited recommendations on which resistance-based multicomponent intervention should be prioritised (5, 7). Our findings suggest that multicomponent resistance-based interventions offer advantages over RT alone, particularly for individuals with functional limitations. These patients often struggle with complex, multi-joint, closed-chain movements that demand intermuscular coordination and postural control (46), and restricted joint range of motion can further reduce muscle engagement and neuromuscular activation, thereby limiting gains in muscle mass, strength, and power, and reducing the translation of these gains into functional improvements (46, 47). Incorporating BT into RT programmes may therefore enhance postural stability and movement control, indirectly boosting RT effectiveness and accelerating recovery of muscle function and overall fitness (48). Consistently, Shen et al. (49) concluded that adding balance training (BT) to RT, with or without nutritional supplementation, was the most effective approach for improving most measures of muscle strength and physical function, a conclusion in agreement with our results. It is noteworthy that, among all exercise modalities assessed, only ARBT achieved clinically meaningful improvements in gait speed. This may reflect the multifactorial nature of gait speed, which depends on the integration of cardiorespiratory endurance, coordination, and balance, making it more reliant on aerobic capacity (50). While aerobic plus resistance training, commonly termed concurrent training, has been reported to have an “interference effect” that could attenuate gains in muscle strength and mass (51, 52), no significant adverse effects were observed in our analysis. Nevertheless, the magnitude of improvements in muscle strength and mass was smaller than for RT-based combinations without aerobic training. However, concurrent ART may be particularly appropriate for the management of sarcopenic obesity, as the integration of both modalities facilitates the reduction of fat mass and the enhancement of muscle mass, thereby promoting a more favourable balance between catabolic and anabolic processes (53). In clinical practice, multicomponent interventions combining RT and BT may be preferable for older adults with sarcopenia who have poor baseline function, balance deficits, or restricted joint mobility, offering both safety and functional benefits. For patients needing improvements in gait speed and endurance, low- to moderate-intensity aerobic training may be incorporated alongside strength-based programmes, with close monitoring of muscle strength and mass to mitigate potential interference effects. Such individualised, progressive, multicomponent approaches could maximise real-world intervention benefits while reducing fall risk.

Beyond RT-based multicomponent programmes, growing attention has been directed toward novel training modalities that may further mitigate muscle loss and functional decline (54). Among these, recent studies have reported that high-intensity interval training (HIIT) can improve muscle strength, functional capacity, and lean mass, with effects that are at least comparable to, and in some cases superior to, those of continuous aerobic exercise (55, 56). These findings suggest that HIIT may help counteract age-related declines in muscle mass and function (55, 56). However, the evidence remains limited, as few high-quality trials are available and almost all have been conducted in robust older adults, making it difficult to generalise the results to populations with sarcopenia (57, 58). Because individuals with sarcopenia are typically more frail and functionally impaired, careful evaluation of the safety and tolerability of HIIT is required before it can be broadly recommended in clinical practice. For patients unable to perform conventional exercise, alternative modalities such as electrical muscle stimulation and whole-body vibration have also been explored (53, 59). Although some studies have suggested potential benefits of these surrogate modalities in sarcopenic populations, the evidence remains sparse and inconclusive. Future research should prioritise large, well-designed trials in sarcopenic populations to establish the efficacy and safety of HIIT and to define the role of emerging exercise surrogates, such as electrical muscle stimulation and whole-body vibration, within multimodal management strategies.

### 4.1 Limitations and future directions

This study has several limitations. First, most comparisons were graded as “very low” to “low” certainty, primarily due to high heterogeneity. Although meta-regressions were conducted to explore potential sources of heterogeneity, no definitive explanatory factors were identified; hence, results should be interpreted cautiously. Second, in subgroup analyses, some interventions were represented by only a few studies, resulting in sparse network connections, which may have reduced statistical power and increased uncertainty in effect estimates. Third, due to the limited number of eligible studies, whey protein, amino acids, and HMB supplementation were combined into a single “protein supplementation” category for analysis. While this improved feasibility, it may have obscured differences in mechanisms between supplement types.

Future research should reduce heterogeneity and strengthen the certainty of evidence by adopting unified diagnostic criteria (e.g., the latest EWGSOP/AWGS) alongside standardized reporting and implementation protocols that clearly specify training intensity, frequency, volume, and progression. Large-scale, multicenter randomized controlled trials are warranted to increase statistical power, enhance external validity, and improve the connectivity of the evidence network, particularly for combined multicomponent exercise and nutritional interventions, which may provide more reliable comparative evidence. Moreover, direct head-to-head trials are needed to compare different forms of protein supplementation (e.g., whey, leucine, EAA, BCAA, HMB), with prespecified stratification according to baseline protein or vitamin D status, sex, and functional capacity. Finally, future studies should broaden outcome assessment to include clinically relevant endpoints such as falls, functional independence, and quality of life, rather than focusing solely on muscle mass and strength, thereby generating evidence with greater clinical applicability and translational value.

## 5 Conclusion

These findings support combined exercise and nutritional interventions as a preferred treatment option for improving muscle strength, muscle mass, and physical function in older adults with sarcopenia. However, the overall certainty of the evidence ranged from low to very low. In particular, multicomponent exercise programmes centred on resistance training and combined with protein supplementation may offer superior benefits for enhancing muscle strength and physical function. These findings provide direct guidance for clinicians and rehabilitation professionals in developing evidence-based management strategies for sarcopenia, and highlight the importance of multicomponent interventions in restoring function and delaying frailty in older adults.

## Data Availability

The original contributions presented in the study are included in the article/Supplementary material, further inquiries can be directed to the corresponding author.
