# Supplementary material for: Exercise and nutrition strategies for sarcopenia in older adults: evidence from a network meta-analysis based on EWGSOP and AWGS criteria
Source: Front Nutr. 2025 Oct 16;12:1685014. doi: 10.3389/fnut.2025.1685014 (PMC12571659; doi:10.3389/fnut.2025.1685014)

**Supplementary Appendix**

**Table of contents**

*[Appendix 1: Search strategy 2](#_Toc18660)*

*[Appendix 2: Characteristics of included studies 6](#_Toc2746)*

*[Appendix 3: Risk of bias of randomized clinical trials 22](#_Toc24155)*

*[Appendix 4: Model Fit, Global Inconsistency, and Heterogeneity Evaluation 24](#_Toc892)*

*[Appendix 5: Node-Splitting Results for Local Inconsistency 25](#_Toc12734)*

*[Appendix 6: Subgroup Analysis 33](#_Toc5368)*

*[Appendix 7: SUCRA and cumulative probability plots 36](#_Toc3193)*

*[Appendix 8: CINeMA Assessment 39](#_Toc16607)*

*[Appendix 9: Funnel plots 46](#_Toc6047)*

*[Appendix10: Network Meta-Regression 48](#_Toc10612)*

*[Appendix11: Sensitivity analysis 51](#_Toc7143)*

# Appendix 1: Search strategy

**Table S1.1** Search strategy of Pubmed

| **#** | **Searches** |
| --- | --- |
| 1 | (((Sarcopenia[MeSH Terms]) OR (sarcopenia[Title/Abstract])) OR (Muscle loss[Title/Abstract])) OR (Sarcopenias[Title/Abstract]) |
| 2 | (((((((((((((((((((((((Training, Resistance[Title/Abstract]) OR (Strength Training[Title/Abstract])) OR (Training, Strength[Title/Abstract])) OR (Weight-Lifting Strengthening Program[Title/Abstract])) OR (Strengthening Programs, Weight-Lifting[Title/Abstract])) OR (Strengthening Program, Weight-Lifting[Title/Abstract])) OR (Weight Lifting Strengthening Program[Title/Abstract])) OR (Weight-Lifting Strengthening Programs[Title/Abstract])) OR (Weight-Lifting Exercise Program[Title/Abstract])) OR (Exercise Programs, Weight-Lifting[Title/Abstract])) OR (Exercise Program, Weight-Lifting[Title/Abstract])) OR (Weight Lifting Exercise Program[Title/Abstract])) OR (Weight-Lifting Exercise Programs[Title/Abstract])) OR (Weight-Bearing Strengthening Program[Title/Abstract])) OR (Strengthening Programs, Weight-Bearing[Title/Abstract])) OR (Strengthening Program, Weight-Bearing[Title/Abstract])) OR (Weight Bearing Strengthening Program[Title/Abstract])) OR (Weight-Bearing Strengthening Programs[Title/Abstract])) OR (Weight-Bearing Exercise Program[Title/Abstract])) OR (Exercise Programs, Weight-Bearing[Title/Abstract])) OR (Exercise Program, Weight-Bearing[Title/Abstract])) OR (Weight Bearing Exercise Program[Title/Abstract])) OR (Weight-Bearing Exercise Programs[Title/Abstract])) OR (Resistance training[MeSH Terms]) |
| 3 | ((((((((((((Whey Proteins[MeSH Terms]) OR (Proteins, Whey[Title/Abstract])) OR (Protein, Whey[Title/Abstract])) OR (Whey Protein[Title/Abstract])) OR (whey supplementation[Title/Abstract])) OR (whey protein supplementation[Title/Abstract])) OR (whey intake[Title/Abstract])) OR (whey protein isolate[Title/Abstract])) OR (whey protein concentrate[Title/Abstract]) |
| 4 | ((((((beta-hydroxyisovaleric acid[MeSH Terms]) OR (beta hydroxy beta methylbutyrate[Title/Abstract])) OR (beta-hydroxy beta-methylbutyrate[Title/Abstract])) OR (3-hydroxyisovaleric acid[Title/Abstract])) OR (beta-hydroxy-beta-methylbutyrate[Title/Abstract])) OR (HMB-d6[Title/Abstract])) OR (HMB[Title/Abstract]) |
| 5 | ((((((Amino Acids, Essential[MeSH Terms]) OR (Acids, Essential Amino[Title/Abstract])) OR (Essential Amino Acid[Title/Abstract])) OR (Acid, Essential Amino[Title/Abstract])) OR (Amino Acid, Essential[Title/Abstract])) OR (Essential Amino Acids[Title/Abstract])) OR (EAA[Title/Abstract]) |
| 6 | ((((Leucine[MeSH Terms]) OR (Leucine, L-Isomer[Title/Abstract])) OR (Leucine, L Isomer[Title/Abstract])) OR (L-Isomer Leucine[Title/Abstract])) OR (L-Leucine[Title/Abstract]) |
| 7 | ((((((((Amino Acids, Branched-Chain[MeSH Terms]) OR (Acids, Branched-Chain Amino[Title/Abstract])) OR (Branched-Chain Amino Acids[Title/Abstract])) OR (Branched-Chain Amino Acid[Title/Abstract])) OR (Acid, Branched-Chain Amino[Title/Abstract])) OR (Amino Acid, Branched-Chain[Title/Abstract])) OR (Branched Chain Amino Acid[Title/Abstract])) OR (Amino Acids, Branched Chain[Title/Abstract])) OR (BCAA[Title/Abstract]) |
| 8 | (((Amino Acids[MeSH Terms]) OR (Acids, Amino[Title/Abstract])) OR (Amino Acid[Title/Abstract])) OR (Acid, Amino[Title/Abstract]) |
| 9 | (((((randomized controlled trial[Publication Type]) OR (randomized)) OR (clinical trials as topic[MeSH Terms])) OR (placebo)) OR (randomly)) OR (trial) OR (randomised) |
| 10 | #2 OR #3 OR #4 OR #5 OR #6 OR #7 OR #8 |
| 11 | #1 AND #9 AND #10 |

**Table S1.2** Search strategy of Web of Science

| **#** | **Searches** |
| --- | --- |
| 1 | TS=("Sarcopenia" OR "Sarcopenias" OR "Muscle loss") |
| 2 | TS=("Resistance training" OR "Training, Resistance" OR "Strength Training" OR "Training, Strength" OR "Weight-Lifting Strengthening Program" OR "Strengthening Programs, Weight-Lifting" OR "Strengthening Program, Weight-Lifting" OR "Weight Lifting Strengthening Program" OR "Weight-Lifting Strengthening Programs" OR "Weight-Lifting Exercise Program" OR "Exercise Programs, Weight-Lifting" OR "Exercise Program, Weight-Lifting" OR "Weight Lifting Exercise Program" OR "Weight-Lifting Exercise Programs" OR "Weight-Bearing Strengthening Program" OR "Strengthening Programs, Weight-Bearing" OR "Strengthening Program, Weight-Bearing" OR "Weight Bearing Strengthening Program" OR "Weight-Bearing Strengthening Programs" OR "Weight-Bearing Exercise Program" OR "Exercise Programs, Weight-Bearing" OR "Exercise Program, Weight-Bearing" OR "Weight Bearing Exercise Program" OR "Weight-Bearing Exercise Programs") |
| 3 | TS=("Whey Proteins" OR "Proteins, Whey" OR "Protein, Whey" OR "Whey Protein" OR "whey supplementation" OR "whey protein supplementation" OR "whey intake" OR "whey protein isolate" OR "whey protein concentrate") |
| 4 | TS=("Amino Acids, Essential" OR "Acids, Essential Amino" OR "Essential Amino Acid" OR "Acid, Essential Amino" OR "Amino Acid, Essential" OR "Essential Amino Acids" OR "EAA") |
| 5 | TS=("Leucine" OR "Leucine, L-Isomer" OR "Leucine, L Isomer" OR "L-Isomer Leucine" OR "L-Leucine") |
| 6 | TS=("beta-hydroxyisovaleric acid" OR "beta hydroxy beta methylbutyrate" OR "beta-hydroxy beta-methylbutyrate" OR "3-hydroxyisovaleric acid" OR "beta-hydroxy-beta-methylbutyrate" OR "HMB-d6" OR "HMB") |
| 7 | TS=("Amino Acids, Branched-Chain" OR "Acids, Branched-Chain Amino" OR "Branched-Chain Amino Acids" OR "Branched-Chain Amino Acid" OR "Acid, Branched-Chain Amino" OR "Amino Acid, Branched-Chain" OR "Branched Chain Amino Acid" OR "Amino Acids, Branched Chain" OR "BCAA") |
| 8 | TS=("Amino Acids" OR "Acids, Amino" OR "Amino Acid" OR "Acid, Amino") |
| 9 | TS=("randomized controlled trial" OR "randomized" OR "clinical trials" OR "placebo" OR "randomly" OR "trial" OR "randomised") |
| 10 | #2 OR #3 OR #4 OR #5 OR #6 OR #7 OR #8 |
| 11 | #1 AND #9 AND #10 |

**Table S1.3** Search strategy of Cochrane Central Register of Controlled Trials

| **#** | **Searches** |
| --- | --- |
| 1 | MeSH descriptor: [Sarcopenia] explode all trees |
| 2 | MeSH descriptor: [Resistance Training] explode all trees |
| 3 | MeSH descriptor: [Amino Acids] explode all trees |
| 4 | MeSH descriptor: [Whey Proteins] explode all trees |
| 5 | MeSH descriptor: [beta-hydroxyisovaleric acid] explode all trees |
| 6 | MeSH descriptor: [Amino Acids, Essential] explode all trees |
| 7 | MeSH descriptor: [Leucine] explode all trees |
| 8 | MeSH descriptor: [Amino Acids, Branched-Chain] explode all trees |
| 9 | #2 OR #3 OR #4 OR #5 OR #6 OR #7 OR #8 |
| 10 | #1 AND #9 |

**Table 1.4** Search strategy of Embase

| **#** | **Searches** |
| --- | --- |
| 1 | 'Sarcopenia'/exp OR sarcopenia:ab,ti OR 'Muscle loss':ab,ti OR sarcopenias:ab,ti |
| 2 | 'Resistance training'/exp OR 'Resistance training':ab,ti OR 'Training, Resistance':ab,ti OR 'Strength Training':ab,ti OR 'Training, Strength':ab,ti OR 'Weight-Lifting Strengthening Program':ab,ti OR 'Strengthening Programs, Weight-Lifting':ab,ti OR 'Strengthening Program, Weight-Lifting':ab,ti OR 'Weight Lifting Strengthening Program':ab,ti OR 'Weight-Lifting Strengthening Programs':ab,ti OR 'Weight-Lifting Exercise Program':ab,ti OR 'Exercise Programs, Weight-Lifting':ab,ti OR 'Exercise Program, Weight-Lifting':ab,ti OR 'Weight Lifting Exercise Program':ab,ti OR 'Weight-Lifting Exercise Programs':ab,ti OR 'Weight-Bearing Strengthening Program':ab,ti OR 'Strengthening Programs, Weight-Bearing':ab,ti OR 'Strengthening Program, Weight-Bearing':ab,ti OR 'Weight Bearing Strengthening Program':ab,ti OR 'Weight-Bearing Strengthening Programs':ab,ti OR 'Weight-Bearing Exercise Program':ab,ti OR 'Exercise Programs, Weight-Bearing':ab,ti OR 'Exercise Program, Weight-Bearing':ab,ti OR 'Weight Bearing Exercise Program':ab,ti OR 'Weight-Bearing Exercise Programs':ab,ti |
| 3 | 'Whey Proteins'/exp OR 'Proteins, Whey':ab,ti OR 'Protein, Whey':ab,ti OR 'Whey Protein':ab,ti OR 'whey supplementation':ab,ti OR 'whey protein supplementation':ab,ti OR 'whey intake':ab,ti OR 'whey protein isolate':ab,ti OR 'whey protein concentrate':ab,ti |
| 4 | 'beta-hydroxyisovaleric acid'/exp OR 'beta hydroxy beta methylbutyrate':ab,ti OR 'beta-hydroxy beta-methylbutyrate':ab,ti OR '3-hydroxyisovaleric acid':ab,ti OR 'beta-hydroxy-beta-methylbutyrate':ab,ti OR 'HMB-d6':ab,ti OR 'HMB':ab,ti |
| 5 | 'Amino Acids, Essential'/exp OR 'Acids, Essential Amino':ab,ti OR 'Essential Amino Acid':ab,ti OR 'Acid, Essential Amino':ab,ti OR 'Amino Acid, Essential':ab,ti OR 'Essential Amino Acids':ab,ti OR 'EAA':ab,ti |
| 6 | 'Leucine'/exp OR 'Leucine, L-Isomer':ab,ti OR 'Leucine, L Isomer':ab,ti OR 'L-Isomer Leucine':ab,ti OR 'L-Leucine':ab,ti |
| 7 | 'Amino Acids, Branched-Chain'/exp OR 'Acids, Branched-Chain Amino':ab,ti OR 'Branched-Chain Amino Acids':ab,ti OR 'Branched-Chain Amino Acid':ab,ti OR 'Acid, Branched-Chain Amino':ab,ti OR 'Amino Acid, Branched-Chain':ab,ti OR 'Branched Chain Amino Acid':ab,ti OR 'Amino Acids, Branched Chain':ab,ti OR 'BCAA':ab,ti |
| 8 | 'Amino Acids'/exp OR 'Acids, Amino':ab,ti OR 'Amino Acid':ab,ti OR 'Acid, Amino':ab,ti |
| 9 | 'randomized controlled trial'/exp OR 'randomized':ab,ti OR 'clinical trials':ab,ti OR 'placebo':ab,ti OR 'randomly':ab,ti OR 'trial':ab,ti OR 'randomised':ab,ti |
| 10 | #2 OR #3 OR #4 OR #5 OR #6 OR #7 OR #8 |
| 11 | #1 AND #9 AND #10 |

# Appendix 2: Characteristics of included studies

**Table S2.1:** Baseline of characteristics of included studies

| Study ID | Group | Age (Mean±SD) | Sample Size | Period  (weeks) | Exercise Frequency (times/week) | Exercise Duration (min) | Exercise Intervention Details | Nutritional Intervention Details | Diagnostic Criteria | Country | Setting |
| --- | --- | --- | --- | --- | --- | --- | --- | --- | --- | --- | --- |
| Amasene et al 2021^1^ | Exercise+Nutrition | 82.9±5.67 | 15 | 12 | 2 | 60 | Resistance and balance training | Received daily supplementation with 21 g whey protein and 6 g leucine | EWGSOP2 | Spain | Institution |
|  | Exercise | 81.2±6.14 | 13 | 12 | 2 | 60 | Resistance and balance training | Isoenergetic placebo | EWGSOP2 | Spain | Institution |
| Bauer et al 2015^2^ | Nutrition | 77.3±6.7 | 172 | 13 |  |  |  | Daily oral intake of vitamin D (800 IU) and leucine-enriched whey protein (20 g protein + 3 g leucine) | EWGSOP2010 | Germany | Institution |
|  | CG | 78.1±7 | 158 | 13 |  |  |  | Isoenergetic, isovolumetric placebo without vitamin D or protein | EWGSOP2010 | Germany | Institution |
| Bo Yacong et al2019^3^ | CG | 74.83±5.94 | 30 | 26 |  |  |  | Isoenergetic placebo supplement | AWGS2014 | China | Institution |
|  | Nutrition | 73.23±6.52 | 30 | 26 |  |  |  | nutritional supplement twice daily (40 g per serving reconstituted in 100–150 mL water), providing a total of 44 g protein (57.5% of energy, primarily whey), 1404 IU vitamin D, and 218 mg vitamin E per day | AWGS2014 | China | Institution |
| Chen et al 2018^4^ | Exercise | 66.7±5.3 | 17 | 8 | 2 | 60 | Resistance training |  | AWGS2014 | China | Community |
|  | CG | 68.3±2.8 | 16 | 8 |  |  |  |  | AWGS2014 | China | Community |
| Hassan et al2016^5^ | Exercise | 85.7±7.0 | 21 | 26 | 2 | 60 | Resistance and balance training |  | EWGSOP 2010 | Australia | Institution |
|  | CG | 86.1±8.2 | 21 | 26 |  |  |  |  | EWGSOP 2010 | Australia | Institution |
| Jung et al 2019^6^ | Exercise | 75±3.9 | 13 | 12 | 3 | 40 | Aerobic, resistance, and balance training |  | AWGS2014 | Korea | Community |
|  | CG | 74.9±5.2 | 13 |  |  |  |  |  | AWGS2014 | Korea | Community |
| Jung et al 2024^7^ | Exercise | 78.14 ± 3.72 | 14 | 12 | 3 | 40 | Aerobic, resistance, and balance training |  | AWGS2014 | Korea | Community |
|  | CG | 78.21 ± 3.72 | 14 |  |  |  |  |  | AWGS2014 | Korea | Community |
| Kemmler et al 2020^8,9^ | Nutrition | 77.8±3.6 | 22 | 52 |  |  |  | Daily protein intake of 1.2 g/kg body weight, with calcium (1000 mg/day) and vitamin D (800 IU/day) supplementation | EWGSOP 2010 | Germany | Community |
|  | Exercise+Nutrition | 79.2±4.7 | 21 | 52 | 2 | 47 | Resistance training | whey protein supplementation targeting 1.5–1.6 g/kg/day protein intake, with additional calcium (1000 mg/day) and vitamin D (800 IU/day) | EWGSOP 2010 | Germany | Community |
| Kyun et al2020^10^ | Exercise | 76.94±9.43 | 19 | 2 | 5 | 50 | Aerobic, resistance, and balance training |  | AWGS 2019 | Korea | Institution |
|  | Exercise | 81.15±4.9 | 19 | 2 | 5 | 50 | Resistance and balance training |  | AWGS 2019 | Korea | Institution |
| Lee et al 2021^11^ | Exercise | 70.13±4.51 | 15 | 12 | 3 | 55 | Resistance training |  | EWGSOP2010 | China | Community |
|  | CG | 71.82±5.33 | 12 | 12 |  |  |  |  | EWGSOP2010 | China | Community |
| Li et al 2020^12^ | Nutrition | 70.04±3.98 | 51 | 12 |  |  |  | nutrition supplementation groups consumed 10 g of whey protein powder with each main meal (total 30 g/day), along with 2 daily doses of fish oil capsules containing 300 mg EPA and 200 mg DHA, and 500 IU of vitamin D₃ (250 IU per dose) | AWGS2014 | China | Community |
|  | Exercise | 73.73±5.69 | 37 | 12 | 3 | 90 | Aerobic and resistance training |  | AWGS2014 | China | Community |
|  | Exercise+Nutrition | 71.52±5.28 | 48 | 12 | 3 | 90 | Aerobic and resistance training | nutrition supplementation groups consumed 10 g of whey protein powder with each main meal (total 30 g/day), along with 2 daily doses of fish oil capsules containing 300 mg EPA and 200 mg DHA, and 500 IU of vitamin D₃ (250 IU per dose) | AWGS2014 | China | Community |
|  | CG | 72.91±6.29 | 33 | 12 |  |  |  | Received general health counseling | AWGS2014 | China | Community |
| Liang et al 2020^13^ | Exercise | 87.3±6 | 30 | 12 | 2 | 55 | Resistance and balance training |  | AWGS | China | Institution |
|  | Exercise | 86.8±4.7 | 30 | 12 | 2 | 55 | Resistance training |  | AWGS | China | Institution |
| Liao et al 2017^14^ | Exercise | 68.42±5.86 | 25 | 12 | 3 | 60 | Resistance training |  | EWGSOP2010 | China | Institution |
|  | CG | 66.39±4.49 | 21 | 12 |  |  |  |  | EWGSOP2010 | China | Institution |
| Liao et al2024^15^ | Nutrition | 73.21±4.98 | 46 | 16 |  |  |  | Received nutrition education only | AWGS2019 | China | Community |
|  | Exercise | 72.04±5.02 | 35 | 16 | 5 | 60 | Aerobic and resistance training | oral peptide-based supplement (185 kcal/day, 24.2 g protein: 11 g plant peptides, 4 g casein peptides, 2.5 g CaHMB) | AWGS2019 | China | Community |
|  | Exercise+Nutrition | 72.68±5.59 | 48 | 16 |  |  | Aerobic and resistance training | oral peptide-based supplement (185 kcal/day, 24.2 g protein: 11 g plant peptides, 4 g casein peptides, 2.5 g CaHMB). | AWGS2019 | China | Community |
|  | CG | 70.52±3.3 | 30 | 16 | 5 | 60 |  | oral peptide-based supplement (185 kcal/day, 24.2 g protein: 11 g plant peptides, 4 g casein peptides, 2.5 g CaHMB). | AWGS2019 | China | Community |
| Lichtenberg et al2019^16^ | Nu | 79.2±4.7 | 22 | 28 |  |  |  | Dietary protein supplementation (1.2 g/kg/day) combined with vitamin D (800 IU/day) | EWGSOP2010 | Germany | Community |
|  | Exercise+Nutrition | 77.8±3.6 | 21 | 28 | 2 | 60 | Resistance training | protein supplementation (1.5 g/kg/day) and vitamin D (800 IU/day) | EWGSOP2010 | Germany | Community |
| Lin et al 2020^17^ | Nutrition | 73.8±8.11 | 28 | 12 |  |  |  | daily intake from supplements was 25.6 g protein, 2.4 g leucine, and 240 IU vitamin D. | AWGS2014 | China | Institution |
|  | CG | 72.5±5.57 | 28 | 12 |  |  |  | Calorie-controlled diet | AWGS2014 | China | Institution |
| Liu et al2024^18^ | CG | 75.6±6.35 | 45 | 12 |  |  |  | Daily activities maintained | AWGS2019 | China | Community |
|  | Exercise+Nutrition | 74.2±4.67 | 41 | 12 | 3 | 60 | Aerobic and resistance training |  | AWGS2019 | China | Community |
| Martínez-Arnau et al2020^19^ | Nutrition | 78.4±8.4 | 23 | 13 |  |  |  | Daily oral supplementation with 6 g L-leucine | EWGSOP2010 | Spain | Institution |
|  | CG | 79.0±7.6 | 19 | 13 |  |  |  | Daily placebo supplementation with 6 g lactose | EWGSOP2010 | Spain | Institution |
| Meza-Valderrama et al 2024^20^ | Exercise+Nutrition | 81.8±8.8 | 17 | 12 | 3 | 60 | Resistance and balance training | Received daily supplementation with 3 g Ca-HMB | EWGSOP2 | Spain | Institution |
|  | Exercise | 81.3±10.2 | 15 | 12 | 3 | 60 | Resistance and balance training |  | EWGSOP2 | Spain | Institution |
| Molnár et al2016^21^ | Exercise | 66.35±1.79 | 17 | 12 | 2 | 30 | Resistance training |  | EWGSOP2010 | USA | Institution |
|  | Exercise+Nutrition | 66.59±1.63 | 17 | 12 | 2 | 30 | Resistance training | Received twice-daily supplementation with FortiFit (Nutricia), providing a total of 40 g whey protein, 20 g EAA (including 3 g leucine), 18 g carbohydrates, 6 g fat, and 1600 IU vitamin D | EWGSOP2010 | USA | Institution |
| Mori et al 2022^22^ | Exercise | 77.6±5.2 | 23 | 24 | 2 | 40 | Resistance training |  | AWGS2014 | Japan | Community |
|  | Exercise+Nutrition | 77.7±3.3 | 23 | 24 | 2 | 40 | Resistance training | Received twice-weekly supplementation with 11 g whey protein and 2.3 g leucine per dose (total 22 g protein and 4.6 g leucine per week) | AWGS2014 | Japan | Community |
| Nasimi et al 2021^23^ | Nutrition | 71.0±6.35 | 33 | 12 |  |  |  | Daily intake of 3 g HMB, 1000 IU vitamin D, and 500 mg vitamin C | AWGS2014 | Iran | Community |
|  | CG | 69.0±13.4 | 33 | 12 |  |  |  |  | AWGS2014 | Iran | Community |
| Nie et al 2023^24^ | Exercise+Nutrition | 66.76±5.15 | 50 | 12 | 5 | 30 | Aerobic and resistance training | Daily supplementation of 400 kcal nutritional formula plus 0.6–1.0 g/kg whey protein powder | AWGS2014 | China | Institution |
|  | CG | 67.28±5.13 | 50 | 12 |  |  |  | Usual care provided | AWGS2014 | China | Institution |
| Rondanelli et al 2024^25^ | Exercise | 79.7±4.8 | 29 | 16 | 5 | 30 | Resistance and balance training |  | EWGSOP2 | Italy | Institution |
|  | Exercise+Nutrition | 79.7±4.8 | 30 | 16 | 5 | 30 | Resistance and balance training | Received daily supplementation with 1.5 g Ca-HMB, 125 mg L-carnosine, 50 mg lactoferrin, 250 mg sodium butyrate, and 150 mg magnesium | EWGSOP2 | Italy | Institution |
| Rufino et al2023^26^ | Exercise | 79.9±7.2 | 20 | 26 | 2 | 65 | Resistance training |  | EWGSOP2010 | Spain | Community |
|  | CG | 79.6±7.7 | 18 | 26 |  |  |  |  | EWGSOP2010 | Spain | Community |
| Seo et al 2021^27^ | Exercise | 70.3±5.38 | 12 | 16 | 3 | 60 | Resistance training |  | EWGSOP2010 | Korea | Institution |
|  | CG | 72.9±4.75 | 10 | 16 |  |  |  |  | EWGSOP2010 | Korea | Institution |
| Tokuda et al2021^28^ | Exercise | 79±2.2 | 15 | 24 | 2 | 40 | Resistance training |  | AWGS2019 | Japan | Community |
|  | Exercise+Nutrition | 78±1.48 | 15 | 24 | 2 | 40 | Resistance training | Received daily supplementation with 3 g EAA | AWGS2019 | Japan | Community |
| Tsekoura et al2018^29^ | Exercise | 74.56±6.04 | 18 | 12 | 5 | 50 | Aerobic, resistance, and balance training |  | EWGSOP2010 | Greece | Institution |
|  | CG | 72.89±8.31 | 18 | 12 |  |  |  | Health education only | EWGSOP2010 | Greece | Institution |
| Wang et al2022^30^ | CG | 69.88±3.29 | 51 | 12 | 10 |  |  | Health education only | EWGSOP2 | China | Community |
|  | Exercise+Nutrition | 70.16±4.32 | 50 | 12 | 10 | 45 | Aerobic and resistance training | 1.2–1.5 g/kg/day protein (≥50% high-quality protein) and 600–800 IU/day vitamin D | EWGSOP2 | China | Community |
|  | Nutrition | 68.18±3.93 | 50 | 12 | 10 |  |  | 1.2–1.5 g/kg/day protein (≥50% high-quality protein) and 600–800 IU/day vitamin D | EWGSOP | China | Community |
|  | Exercise | 69.72±3.60 | 50 | 12 | 10 | 45 | Aerobic and resistance training | Internet-based training program (daily 40–60 min aerobic/resistance) | EWGSOP | China | Community |
| Wei et al2022^31^ | Exercise | 66.87±3.84 | 30 | 24 | 3 | 60 | Resistance training |  | AWGS2016 | China | Institution |
|  | CG | 65.42±3.100 | 30 | 24 |  |  |  |  | AWGS2016 | China | Institution |
| Yamada et al2019^32^ | Exercise+Nutrition | 84.9±5.6 | 28 | 12 | 3 | 30 | Resistance training | Received daily supplementation with 10 g whey protein and 800 IU vitamin D | AWGS2014 | Japan | Community |
|  | Exercise | 84.7±5.1 | 28 | 12 | 3 | 30 | Resistance training |  | AWGS2014 | Japan | Community |
|  | CG | 83.9±5.7 | 28 | 12 |  |  |  |  | AWGS2014 | Japan | Community |
| Yang et al 2023^33^ | Exercise+Nutrition | 72.89±7.02 | 18 | 12 | 2 | 40 | Resistance training | Received twice-daily supplementation with 1.5 g Ca-HMB (3 g/day total) | AWGS2019 | China | Community |
|  | Exercise | 71.44±5.22 | 16 | 12 | 2 | 40 | Resistance training | isoenergetic placebo | AWGS2019 | China | Community |
| Yuenyongchaiwat et al 2022^34^ | Exercise | 69.23±6.71 | 30 | 12 | 7 |  | Aerobic and resistance training |  | AWGS2019 | Thailand | Community |
|  | CG | 71.93±5.19 | 30 | 12 |  |  |  | Maintained daily activity | AWGS2019 | Thailand | Community |
| Zhu et al 2018^35^ | CG | 72.2±6.6 | 37 | 12 |  |  |  | Maintained usual activities | AWGS2014 | China | Community |
|  | Exercise | 74.5±7.1 | 40 | 12 | 3 | 90 | Aerobic and resistance training |  | AWGS2014 | China | Community |
|  | Exercise+Nutrition | 74.8±6.9 | 36 | 12 | 3 | 90 | Aerobic and resistance training |  | AWGS2014 | China | Community |

**References**

1. Amasene, M. *et al.* Effects of resistance training intervention along with leucine-enriched whey protein supplementation on sarcopenia and frailty in post-hospitalized older adults: Preliminary findings of a randomized controlled trial. *JCM* **11**, 97 (2021).

2. Bauer, J. M. *et al.* Effects of a vitamin D and leucine-enriched whey protein nutritional supplement on measures of sarcopenia in older adults, the PROVIDE study: A randomized, double-blind, placebo-controlled trial. *Journal of the American Medical Directors Association* **16**, 740–747 (2015).

3. Bo, Y. *et al.* A high whey protein, vitamin D and E supplement preserves muscle mass, strength, and quality of life in sarcopenic older adults: A double-blind randomized controlled trial. *Clinical Nutrition* **38**, 159–164 (2019).

4. Chen, H.-T., Wu, H.-J., Chen, Y.-J., Ho, S.-Y. & Chung, Y.-C. Effects of 8-week kettlebell training on body composition, muscle strength, pulmonary function, and chronic low-grade inflammation in elderly women with sarcopenia. *Experimental Gerontology* **112**, 112–118 (2018).

5. Hassan, B. H. *et al.* Impact of resistance training on sarcopenia in nursing care facilities: A pilot study. *Geriatric Nursing* **37**, 116–121 (2016).

6. Jung, W.-S., Kim, Y.-Y. & Park, H.-Y. Circuit training improvements in korean women with sarcopenia. *Percept Mot Skills* **126**, 828–842 (2019).

7. Jung, W.-S., Ahn, H., Kim, S.-W. & Park, H.-Y. Effects of 12-week circuit exercise intervention on blood pressure, vascular function, and inflammatory cytokines in obese older women with sarcopenia. *Rev. Cardiovasc. Med.* **25**, 185 (2024).

8. Kemmler, W., Kohl, M., Jakob, F., Engelke, K. & Von Stengel, S. Effects of High Intensity Dynamic Resistance Exercise and Whey Protein Supplements on Osteosarcopenia in Older Men with Low Bone and Muscle Mass. Final Results of the Randomized Controlled FrOST Study. *Nutrients* **12**, 2341 (2020).

9. Kemmler, W. *et al.* Effects of high-intensity resistance training on fitness and fatness in older men with osteosarcopenia. *Front. Physiol.* **11**, 1014 (2020).

10. Oh, M.-K. *et al.* Efficacy of combined antigravity treadmill and conventional rehabilitation after hip fracture in patients with sarcopenia. *The Journals of Gerontology: Series A* **75**, e173–e181 (2020).

11. Lee, Y.-H. *et al.* Effects of progressive elastic band resistance exercise for aged osteosarcopenic adiposity women. *Experimental Gerontology* **147**, 111272 (2021).

12. Li, Z. *et al.* Effects of nutrition supplementation and physical exercise on muscle mass, muscle strength and fat mass among sarcopenic elderly: A randomized controlled trial. *Appl. Physiol. Nutr. Metab.* **46**, 494–500 (2021).

13. Liang, Y., Wang, R., Jiang, J., Tan, L. & Yang, M. A randomized controlled trial of resistance and balance exercise for sarcopenic patients aged 80–99 years. *Sci Rep* **10**, 18756 (2020).

14. Liao, C.-D. *et al.* Effects of elastic resistance exercise on body composition and physical capacity in older women with sarcopenic obesity: A CONSORT-compliant prospective randomized controlled trial. *Medicine* **96**, e7115 (2017).

15. Liao, X. *et al.* Effects of oral oligopeptide preparation and exercise intervention in older people with sarcopenia: A randomized controlled trial. *BMC Geriatr* **24**, 260 (2024).

16. Lichtenberg, T., Von Stengel, S., Sieber, C. & Kemmler, W. The favorable effects of a high-intensity resistance training on sarcopenia in older community-dwelling men with osteosarcopenia: The randomized controlled FrOST study. *CIA* **Volume 14**, 2173–2186 (2019).

17. Lin, C.-C., Shih, M.-H., Chen, C.-D. & Yeh, S.-L. Effects of adequate dietary protein with whey protein, leucine, and vitamin D supplementation on sarcopenia in older adults: An open-label, parallel-group study. *Clinical Nutrition* **40**, 1323–1329 (2021).

18. Liu, M. *et al.* Graded progressive home-based resistance combined with aerobic exercise in community-dwelling older adults with sarcopenia: A randomized controlled trial. *CIA* **Volume 19**, 1581–1595 (2024).

19. Martínez-Arnau, F. M. *et al.* Effects of leucine administration in sarcopenia: A randomized and placebo-controlled clinical trial. *Nutrients* **12**, 932 (2020).

20. Meza-Valderrama, D. *et al.* Supplementation with β-hydroxy-β-methylbutyrate after resistance training in post-acute care patients with sarcopenia: A randomized, double-blind placebo-controlled trial. *Archives of Gerontology and Geriatrics* **119**, 105323 (2024).

21. Molnár, A. *et al.* Special nutrition intervention is required for muscle protective efficacy of physical exercise in elderly people at highest risk of sarcopenia. *Physiology International* **103**, 368–376 (2016).

22. Mori, H. & Tokuda, Y. De-training effects following leucine-enriched whey protein supplementation and resistance training in older adults with sarcopenia: A randomized controlled trial with 24 weeks of follow-up. *The Journal of nutrition, health and aging* **26**, 994–1002 (2022).

23. Nasimi, N. *et al.* A novel fortified dairy product and sarcopenia measures in sarcopenic older adults: A double-blind randomized controlled trial. *Journal of the American Medical Directors Association* **22**, 809–815 (2021).

24. Nie, N. *et al.* Clinical efficacy of nutritional intervention combined with muscle exercise on sarcopenia patients with femoral fracture: A pilot randomized controlled trial. *BioMed Research International* **2023**, 3222686 (2023).

25. Rondanelli, M. *et al.* A patented dietary supplement (hydroxy-methyl-butyrate, carnosine, magnesium, butyrate, lactoferrin) is a promising therapeutic target for age-related sarcopenia through the regulation of gut permeability: A randomized controlled trial. *Nutrients* **16**, 1369 (2024).

26. Flor-Rufino, C. *et al.* Fat infiltration and muscle hydration improve after high-intensity resistance training in women with sarcopenia. A randomized clinical trial. *Maturitas* **168**, 29–36 (2023).

27. Sen, E. I. *et al.* Effect of home-based exercise program on physical function and balance in older adults with sarcopenia: A multicenter randomized controlled study. *Journal of Aging and Physical Activity* **29**, 1010–1017 (2021).

28. Tokuda, Y. & Mori, H. Essential amino acid and tea catechin supplementation after resistance exercise improves skeletal muscle mass in older adults with sarcopenia: An open-label, pilot, randomized controlled trial. *Journal of the American Nutrition Association* **42**, 255–262 (2023).

29. Tsekoura, M. *et al.* The effects of group and home-based exercise programs in elderly with sarcopenia: A randomized controlled trial. *JCM* **7**, 480 (2018).

30. Wang, H., Shen, B. & Bo, J. Examining situational interest in physical education: A new inventory. *Journal of Teaching in Physical Education* **41**, 270–277 (2022).

31. Wei, M. *et al.* Hybrid exercise program for sarcopenia in older adults: The effectiveness of explainable artificial intelligence-based clinical assistance in assessing skeletal muscle area. *IJERPH* **19**, 9952 (2022).

32. Yamada, M. *et al.* Synergistic effect of bodyweight resistance exercise and protein supplementation on skeletal muscle in sarcopenic or dynapenic older adults. *Geriatrics Gerontology Int* **19**, 429–437 (2019).

33. Yang, C. *et al.* Effects of beta-hydroxy-beta-methylbutyrate supplementation on older adults with sarcopenia: A randomized, double-blind, placebo-controlled study. *The Journal of nutrition, health and aging* **27**, 329–339 (2023).

34. Yoshimura, Y., Uchida, K., Jeong, S. & Yamaga, M. Effects of nutritional supplements on muscle mass and activities of daily living in elderly rehabilitation patients with decreased muscle mass: A randomized controlled trial. *The Journal of nutrition, health and aging* **20**, 185–191 (2016).

35. Zhu, Y. *et al.* Tai chi and whole-body vibrating therapy in sarcopenic men in advanced old age: A clinical randomized controlled trial. *Eur J Ageing* **16**, 273–282 (2019).

# Appendix 3: Risk of bias of randomized clinical trials

**Table S3.1:** Study level risk of bias assessment using Cochrane risk of bias tool 2.0 for assessing risk of bias of randomized clinical trials.

| Unique ID | Randomization process | Deviations from intended interventions | Missing outcome data | Measurement of the outcome | Selection of the reported result | Overall Bias |
| --- | --- | --- | --- | --- | --- | --- |
| Amasene et al2021 | Some concerns | Some concerns | High | Low | Some concerns | High |
| Bauer et al2015 | Low | Low | Low | Low | Low | Low |
| Bo et al2019 | Low | Low | Low | Low | Low | Low |
| Chen et al 2018 | Some concerns | Low | Low | Low | Some concerns | Some concerns |
| Hassan et al2016 | Some concerns | Low | Low | Low | Some concerns | Some concerns |
| Jung et al2019 | Some concerns | Some concerns | Low | Low | Some concerns | Some concerns |
| Jung et al2024 | Some concerns | Some concerns | Low | Low | Some concerns | Some concerns |
| Kemmler et al2020a | Low | Low | Low | Low | Low | Low |
| Kemmler et al2020b | Low | Low | Low | Low | Low | Low |
| Kyun et al2020 | Low | Some concerns | Some concerns | Low | Low | Some concerns |
| Lee et al2021 | Low | Low | Low | Low | Low | Low |
| Li et al2020 | High | High | High | Low | Some concerns | High |
| Liang et al2020 | Some concerns | Low | Low | Low | Low | Some concerns |
| Liao et al 2017 | Some concerns | Low | Low | Low | Some concerns | Some concerns |
| Liao et al2024 | Some concerns | High | High | Low | Some concerns | High |
| Lichtenberg et al2019 | Low | Low | Low | Low | Low | Low |
| Lin et al2020 | Some concerns | High | High | Low | Some concerns | High |
| Liu et al2024 | Low | Low | Low | Low | Low | Low |
| Martínez-Arnau et al2020 | Low | Low | Low | Low | Low | Low |
| Meza-Valderrama et al2024 | Low | Low | Low | Low | Low | Low |
| Molnar et al2016 | High | Some concerns | Low | Low | Some concerns | High |
| Mori et al2022 | Low | Some concerns | Low | Low | Some concerns | Some concerns |
| Nasimi et al2021 | Low | Low | Low | Low | Low | Low |
| Nie et al2023 | Some concerns | Some concerns | Low | Low | Some concerns | Some concerns |
| Rondanelli et al2024 | Low | Low | Low | Low | Low | Low |
| Rufino et al2023 | Low | Low | Low | Low | Low | Low |
| Seo et al2021 | Some concerns | Low | Low | Low | Some concerns | Some concerns |
| Tokuda et al2021 | Low | Some concerns | Low | Low | Low | Some concerns |
| Tsekoura et al2018 | Low | Low | Low | Low | Low | Low |
| Wang et al2022 | High | Some concerns | Low | Low | Low | High |
| Wei et al2022 | Some concerns | Some concerns | Low | Low | Some concerns | Some concerns |
| Yamada et al2019 | Some concerns | Low | Low | Low | Some concerns | Some concerns |
| Yang et al2023 | Low | Low | Low | Low | Low | Low |
| Yuenyongchaiwat et al2022 | Some concerns | Low | Low | Low | Some concerns | Some concerns |
| Zhu et al2018 | Low | Low | Low | Low | Low | Low |

**Appendix 4: Model Fit, Global Inconsistency, and Heterogeneity Evaluation**

**Table S4.1:** Evaluation of consistency, global inconsistency, and heterogeneity (τ²) for outcomes.

| **Outcomes** | **Study** | **τ** 2 | **Consistency** | | | **Global inconsistency** | | |
| --- | --- | --- | --- | --- | --- | --- | --- | --- |
|  |  |  | **Residual deviance** | **pD** | **DIC** | **Residual deviance** | **pD** | **DIC** |
| Handgrip strength | 30 studies,  n= 1954 | 3.73 | 65.5 | 56.7 | 122.2 | 66.1 | 56.8 | 122.9 |
| Gait speed | 27 studies,  n= 1804 | 0.006 | 60.5 | 50.1 | 110.2 | 59 | 50.5 | 109.4 |
| ASMI | 17 studies,  n= 1137 | 0.067 | 34 | 24.1 | 58 | 33.2 | 23.6 | 56.8 |

**Appendix 5: Node-Splitting Results for Local Inconsistency**

**Figure S5.1**: Density Plots for Node-Splitting Analysis of Handgrip Strength

**
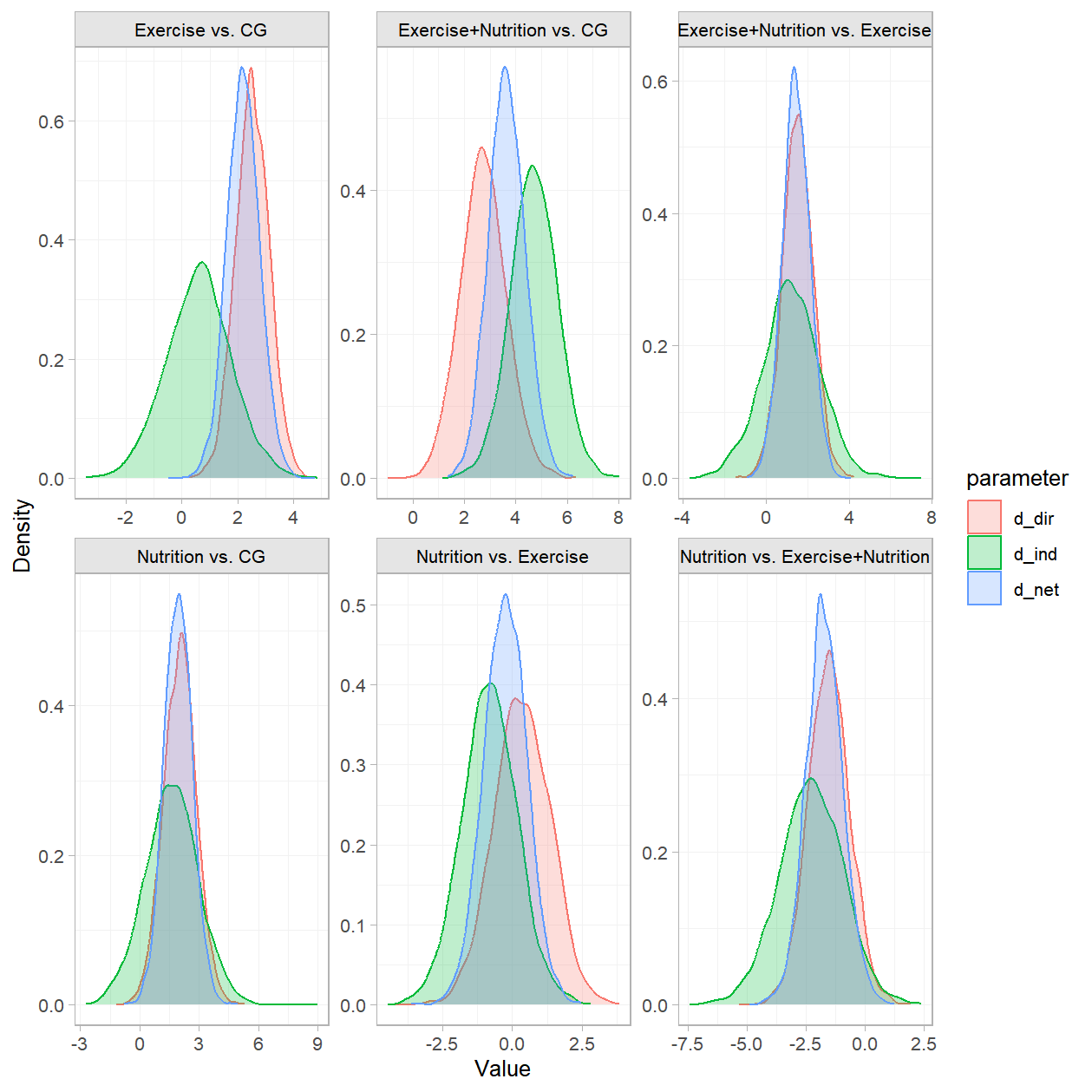
**

**Table S5.2:** Side-splitting of Handgrip Strength. Inconsistency test between direct and indirect treatment comparisons with heterogeneity (τ²) estimates in mixed treatment comparison.

| **Comparison** |  | **mean** | **sd** | **2.50%** | **25%** | **50%** | **75%** | **97.50%** | **Bayesian p-value** |
| --- | --- | --- | --- | --- | --- | --- | --- | --- | --- |
| **Exercise**  **vs. CG** | **d_net** | 2.21 | 0.58 | 1.07 | 1.81 | 2.2 | 2.6 | 3.37 | 0.12 |
|  | **d_dir** | 2.53 | 0.61 | 1.34 | 2.13 | 2.52 | 2.94 | 3.71 |  |
|  | **d_ind** | 0.62 | 1.16 | -1.66 | -0.16 | 0.63 | 1.38 | 2.96 |  |
|  | **omega** | 1.91 | 1.22 | -0.49 | 1.11 | 1.92 | 2.75 | 4.32 |  |
|  | **tau** | 1.91 | 0.34 | 1.33 | 1.68 | 1.88 | 2.11 | 2.69 |  |
|  | **tau_**  **consistency** | 1.97 | 0.34 | 1.4 | 1.74 | 1.93 | 2.17 | 2.73 |  |
| **Exercise+Nutrition**  **vs. CG** | **d_net** | 3.66 | 0.71 | 2.3 | 3.18 | 3.65 | 4.13 | 5.09 | 0.086 |
|  | **d_dir** | 2.75 | 0.9 | 1 | 2.15 | 2.73 | 3.32 | 4.56 |  |
|  | **d_ind** | 4.71 | 0.93 | 2.87 | 4.1 | 4.7 | 5.33 | 6.52 |  |
|  | **omega** | -1.96 | 1.15 | -4.16 | -2.75 | -1.96 | -1.19 | 0.27 |  |
|  | **tau** | 1.88 | 0.34 | 1.31 | 1.64 | 1.85 | 2.08 | 2.63 |  |
|  | **tau_**  **consistency** | 1.97 | 0.34 | 1.4 | 1.74 | 1.93 | 2.17 | 2.73 |  |
| **Nutrition**  **vs. CG** | **d_net** | 1.96 | 0.71 | 0.57 | 1.47 | 1.96 | 2.44 | 3.37 | 0.77 |
|  | **d_dir** | 2.04 | 0.83 | 0.42 | 1.49 | 2.06 | 2.59 | 3.69 |  |
|  | **d_ind** | 1.63 | 1.35 | -1.07 | 0.73 | 1.63 | 2.52 | 4.28 |  |
|  | **omega** | 0.42 | 1.47 | -2.53 | -0.55 | 0.42 | 1.37 | 3.38 |  |
|  | **tau** | 2.01 | 0.34 | 1.42 | 1.77 | 1.98 | 2.22 | 2.75 |  |
|  | **tau_**  **consistency** | 1.97 | 0.34 | 1.4 | 1.74 | 1.93 | 2.17 | 2.73 |  |
| **Exercise+Nutrition**  **vs. Exercise** | **d_net** | -0.25 | 0.77 | -1.78 | -0.77 | -0.24 | 0.26 | 1.25 | 0.85 |
|  | **d_dir** | 0.35 | 1.02 | -1.69 | -0.34 | 0.34 | 1.05 | 2.3 |  |
|  | **d_ind** | -0.81 | 1 | -2.81 | -1.47 | -0.81 | -0.14 | 1.19 |  |
|  | **omega** | 1.16 | 1.3 | -1.45 | 0.3 | 1.14 | 2.02 | 3.65 |  |
|  | **tau** | 1.99 | 0.34 | 1.4 | 1.75 | 1.96 | 2.2 | 2.76 |  |
|  | **tau_**  **consistency** | 1.97 | 0.34 | 1.4 | 1.74 | 1.93 | 2.17 | 2.73 |  |
| **Nutrition**  **vs. Exercise** | **d_net** | -1.71 | 0.79 | -3.24 | -2.22 | -1.72 | -1.19 | -0.1 | 0.37 |
|  | **d_dir** | -1.52 | 0.89 | -3.22 | -2.11 | -1.52 | -0.94 | 0.22 |  |
|  | **d_ind** | -2.27 | 1.36 | -5 | -3.16 | -2.28 | -1.34 | 0.33 |  |
|  | **omega** | 0.76 | 1.48 | -2.15 | -0.21 | 0.73 | 1.7 | 3.74 |  |
|  | **tau** | 2.01 | 0.34 | 1.43 | 1.77 | 1.97 | 2.21 | 2.75 |  |
|  | **tau_**  **consistency** | 1.97 | 0.34 | 1.4 | 1.74 | 1.93 | 2.17 | 2.73 |  |
| **Nutrition**  **vs. Exercise+Nutrition** | **d_net** | -1.71 | 0.79 | -3.24 | -2.22 | -1.72 | -1.19 | -0.1 | 0.61 |
|  | **d_dir** | -1.52 | 0.89 | -3.22 | -2.11 | -1.52 | -0.94 | 0.22 |  |
|  | **d_ind** | -2.27 | 1.36 | -5 | -3.16 | -2.28 | -1.34 | 0.33 |  |
|  | **omega** | 0.76 | 1.48 | -2.15 | -0.21 | 0.73 | 1.7 | 3.74 |  |
|  | **tau** | 2.01 | 0.34 | 1.43 | 1.77 | 1.97 | 2.21 | 2.75 |  |
|  | **tau_**  **consistency** | 1.97 | 0.34 | 1.4 | 1.74 | 1.93 | 2.17 | 2.73 |  |

**Figure S5.2**: Density Plots for Node-Splitting Analysis of Gait speed


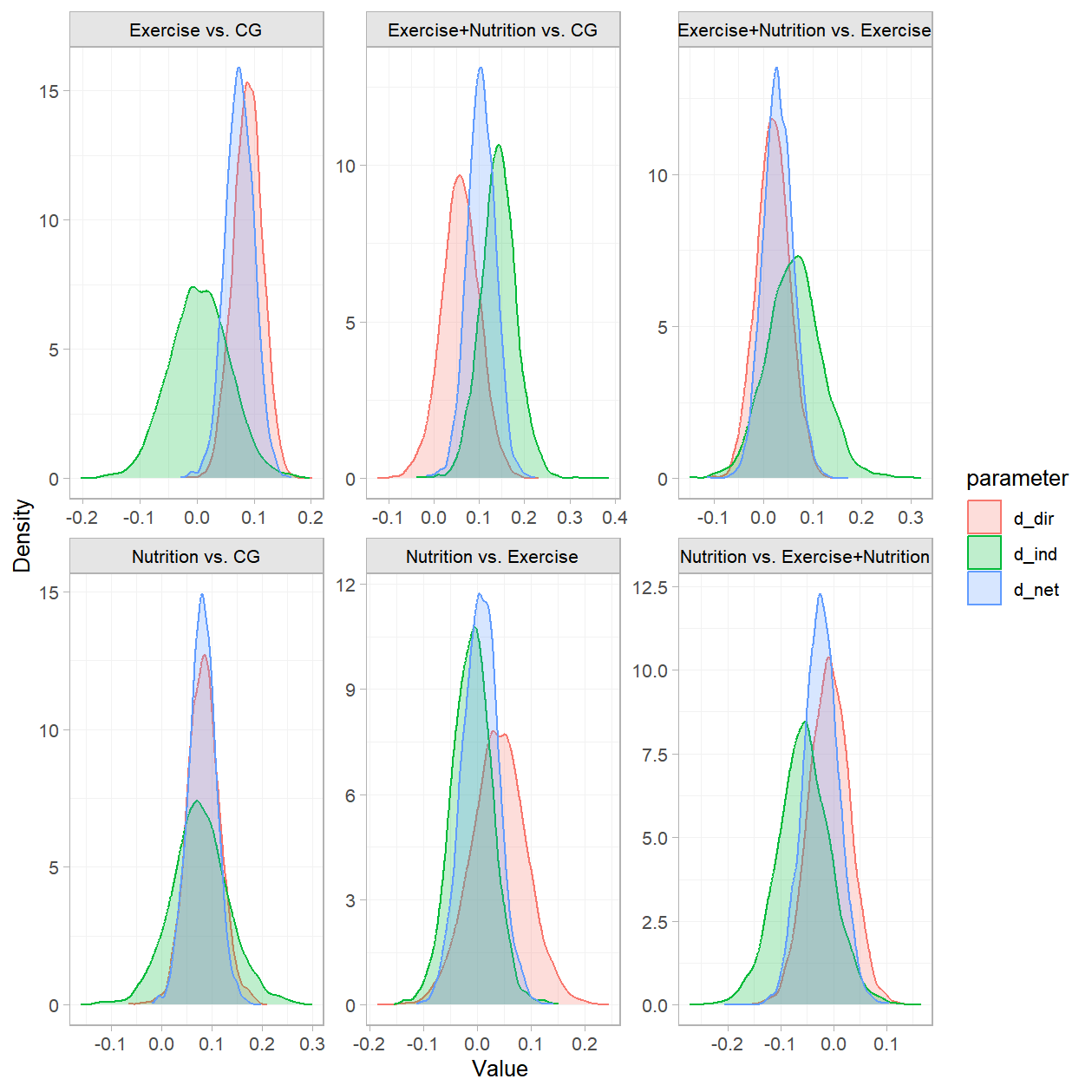


**Table S5.3:** Side-splitting of Gait speed. Inconsistency test between direct and indirect treatment comparisons with heterogeneity (τ²) estimates in mixed treatment comparison.

| **Comparison** |  | **mean** | **sd** | **2.50%** | **25%** | **50%** | **75%** | **97.50%** | **Bayesian p-value** |
| --- | --- | --- | --- | --- | --- | --- | --- | --- | --- |
| **Exercise**  **vs. CG** | **d_net** | 0.07 | 0.03 | 0.03 | 0.06 | 0.07 | 0.09 | 0.12 | 0.12 |
|  | **d_dir** | 0.09 | 0.03 | 0.04 | 0.07 | 0.09 | 0.11 | 0.14 |  |
|  | **d_ind** | 0 | 0.05 | -0.1 | -0.03 | 0 | 0.04 | 0.11 |  |
|  | **omega** | 0.08 | 0.06 | -0.03 | 0.05 | 0.08 | 0.12 | 0.19 |  |
|  | **tau** | 0.07 | 0.02 | 0.05 | 0.06 | 0.07 | 0.08 | 0.11 |  |
|  | **tau_**  **consistency** | 0.08 | 0.02 | 0.05 | 0.07 | 0.07 | 0.09 | 0.11 |  |
| **Exercise+Nutrition**  **vs. CG** | **d_net** | 0.1 | 0.03 | 0.04 | 0.08 | 0.1 | 0.13 | 0.16 | 0.13 |
|  | **d_dir** | 0.06 | 0.04 | -0.03 | 0.03 | 0.06 | 0.09 | 0.14 |  |
|  | **d_ind** | 0.14 | 0.04 | 0.06 | 0.12 | 0.14 | 0.17 | 0.22 |  |
|  | **omega** | -0.08 | 0.06 | -0.2 | -0.12 | -0.08 | -0.05 | 0.03 |  |
|  | **tau** | 0.07 | 0.02 | 0.05 | 0.06 | 0.07 | 0.08 | 0.11 |  |
|  | **tau_**  **consistency** | 0.08 | 0.02 | 0.05 | 0.07 | 0.07 | 0.09 | 0.11 |  |
| **Nutrition**  **vs. CG** | **d_net** | 0.08 | 0.03 | 0.03 | 0.06 | 0.08 | 0.1 | 0.14 | 0.91 |
|  | **d_dir** | 0.08 | 0.03 | 0.02 | 0.06 | 0.08 | 0.1 | 0.15 |  |
|  | **d_ind** | 0.08 | 0.06 | -0.04 | 0.04 | 0.08 | 0.11 | 0.2 |  |
|  | **omega** | 0.01 | 0.06 | -0.12 | -0.03 | 0.01 | 0.05 | 0.13 |  |
|  | **tau** | 0.08 | 0.02 | 0.05 | 0.07 | 0.08 | 0.09 | 0.11 |  |
|  | **tau_**  **consistency** | 0.08 | 0.02 | 0.05 | 0.07 | 0.07 | 0.09 | 0.11 |  |
| **Exercise+Nutrition**  **vs. Exercise** | **d_net** | 0.03 | 0.03 | -0.03 | 0.01 | 0.03 | 0.05 | 0.09 | 0.46 |
|  | **d_dir** | 0.02 | 0.03 | -0.05 | 0 | 0.02 | 0.04 | 0.09 |  |
|  | **d_ind** | 0.06 | 0.06 | -0.04 | 0.03 | 0.06 | 0.1 | 0.17 |  |
|  | **omega** | -0.04 | 0.06 | -0.17 | -0.08 | -0.04 | 0 | 0.08 |  |
|  | **tau** | 0.08 | 0.02 | 0.05 | 0.07 | 0.08 | 0.09 | 0.11 |  |
|  | **tau_**  **consistency** | 0.08 | 0.02 | 0.05 | 0.07 | 0.07 | 0.09 | 0.11 |  |
| **Nutrition**  **vs. Exercise** | **d_net** | 0.01 | 0.03 | -0.06 | -0.02 | 0.01 | 0.03 | 0.07 | 0.38 |
|  | **d_dir** | 0.04 | 0.05 | -0.06 | 0.01 | 0.04 | 0.07 | 0.14 |  |
|  | **d_ind** | -0.01 | 0.04 | -0.08 | -0.03 | -0.01 | 0.02 | 0.07 |  |
|  | **omega** | 0.05 | 0.06 | -0.07 | 0.01 | 0.05 | 0.09 | 0.16 |  |
|  | **tau** | 0.08 | 0.02 | 0.05 | 0.07 | 0.08 | 0.09 | 0.11 |  |
|  | **tau_**  **consistency** | 0.08 | 0.02 | 0.05 | 0.07 | 0.07 | 0.09 | 0.11 |  |
| **Nutrition**  **vs. Exercise+Nutrition** | **d_net** | -0.02 | 0.03 | -0.09 | -0.05 | -0.02 | 0 | 0.04 | 0.41 |
|  | **d_dir** | -0.01 | 0.04 | -0.08 | -0.03 | -0.01 | 0.02 | 0.07 |  |
|  | **d_ind** | -0.06 | 0.05 | -0.16 | -0.09 | -0.06 | -0.02 | 0.05 |  |
|  | **omega** | 0.05 | 0.06 | -0.07 | 0.01 | 0.05 | 0.09 | 0.17 |  |
|  | **tau** | 0.08 | 0.02 | 0.05 | 0.07 | 0.08 | 0.09 | 0.11 |  |
|  | **tau_**  **consistency** | 0.08 | 0.02 | 0.05 | 0.07 | 0.07 | 0.09 | 0.11 |  |

**Figure S5.3**: Density Plots for Node-Splitting Analysis of ASMI

**
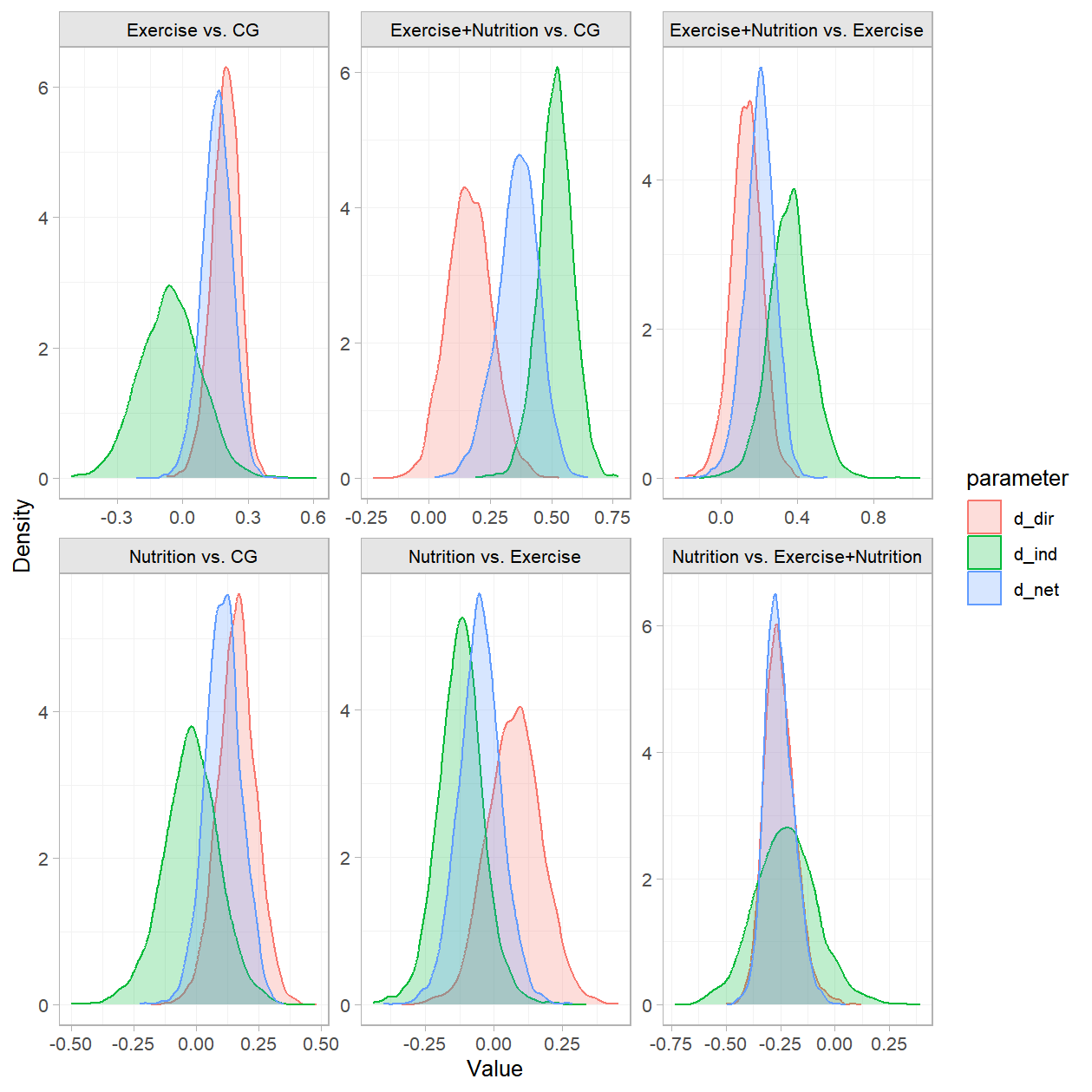
**

**Table S5.4:** Side-splitting of ASMI. Inconsistency test between direct and indirect treatment comparisons with heterogeneity (τ²) estimates in mixed treatment comparison.

| **Comparison** |  | **mean** | **sd** | **2.50%** | **25%** | **50%** | **75%** | **97.50%** | **Bayesian p-value** |
| --- | --- | --- | --- | --- | --- | --- | --- | --- | --- |
| **Exercise**  **vs. CG** | **d_net** | 0.16 | 0.07 | 0.02 | 0.11 | 0.16 | 0.2 | 0.29 | 0.09 |
|  | **d_dir** | 0.2 | 0.06 | 0.06 | 0.16 | 0.2 | 0.24 | 0.31 |  |
|  | **d_ind** | -0.06 | 0.14 | -0.33 | -0.15 | -0.06 | 0.03 | 0.21 |  |
|  | **omega** | 0.25 | 0.15 | -0.05 | 0.16 | 0.26 | 0.35 | 0.53 |  |
|  | **tau** | 0.09 | 0.05 | 0 | 0.05 | 0.08 | 0.12 | 0.2 |  |
|  | **tau_**  **consistency** | 0.12 | 0.05 | 0.02 | 0.08 | 0.12 | 0.15 | 0.22 |  |
| **Exercise+Nutrition**  **vs. CG** | **d_net** | 0.36 | 0.09 | 0.18 | 0.31 | 0.37 | 0.42 | 0.52 | 0.01 |
|  | **d_dir** | 0.17 | 0.09 | 0 | 0.1 | 0.16 | 0.23 | 0.35 |  |
|  | **d_ind** | 0.52 | 0.07 | 0.37 | 0.47 | 0.52 | 0.56 | 0.65 |  |
|  | **omega** | -0.35 | 0.1 | -0.56 | -0.42 | -0.35 | -0.28 | -0.15 |  |
|  | **tau** | 0.05 | 0.04 | 0 | 0.02 | 0.05 | 0.07 | 0.14 |  |
|  | **tau_**  **consistency** | 0.12 | 0.05 | 0.02 | 0.08 | 0.12 | 0.15 | 0.22 |  |
| **Nutrition**  **vs. CG** | **d_net** | 0.11 | 0.07 | -0.04 | 0.06 | 0.11 | 0.15 | 0.25 | 0.14 |
|  | **d_dir** | 0.16 | 0.08 | 0.01 | 0.11 | 0.16 | 0.21 | 0.31 |  |
|  | **d_ind** | -0.02 | 0.11 | -0.25 | -0.09 | -0.02 | 0.05 | 0.2 |  |
|  | **omega** | 0.18 | 0.13 | -0.07 | 0.1 | 0.18 | 0.26 | 0.44 |  |
|  | **tau** | 0.11 | 0.05 | 0.01 | 0.07 | 0.11 | 0.14 | 0.22 |  |
|  | **tau_**  **consistency** | 0.12 | 0.05 | 0.02 | 0.08 | 0.12 | 0.15 | 0.22 |  |
| **Exercise+Nutrition**  **vs. Exercise** | **d_net** | 0.2 | 0.08 | 0.04 | 0.16 | 0.21 | 0.26 | 0.35 | 0.053 |
|  | **d_dir** | 0.13 | 0.08 | -0.03 | 0.08 | 0.14 | 0.19 | 0.28 |  |
|  | **d_ind** | 0.36 | 0.11 | 0.14 | 0.29 | 0.36 | 0.43 | 0.58 |  |
|  | **omega** | -0.23 | 0.12 | -0.47 | -0.31 | -0.22 | -0.15 | 0 |  |
|  | **tau** | 0.1 | 0.05 | 0.01 | 0.07 | 0.1 | 0.14 | 0.21 |  |
|  | **tau_**  **consistency** | 0.12 | 0.05 | 0.02 | 0.08 | 0.12 | 0.15 | 0.22 |  |
| **Nutrition**  **vs. Exercise** | **d_net** | -0.05 | 0.08 | -0.21 | -0.1 | -0.05 | 0 | 0.11 | 0.088 |
|  | **d_dir** | 0.08 | 0.1 | -0.11 | 0.01 | 0.08 | 0.14 | 0.27 |  |
|  | **d_ind** | -0.12 | 0.08 | -0.29 | -0.17 | -0.12 | -0.07 | 0.05 |  |
|  | **omega** | 0.2 | 0.11 | -0.04 | 0.13 | 0.2 | 0.27 | 0.42 |  |
|  | **tau** | 0.1 | 0.05 | 0.01 | 0.06 | 0.1 | 0.13 | 0.21 |  |
|  | **tau_**  **consistency** | 0.12 | 0.05 | 0.02 | 0.08 | 0.12 | 0.15 | 0.22 |  |
| **Nutrition**  **vs. Exercise+Nutrition** | **d_net** | -0.25 | 0.07 | -0.37 | -0.3 | -0.26 | -0.21 | -0.11 | 0.83 |
|  | **d_dir** | -0.25 | 0.07 | -0.37 | -0.3 | -0.26 | -0.21 | -0.09 |  |
|  | **d_ind** | -0.23 | 0.14 | -0.51 | -0.32 | -0.23 | -0.13 | 0.07 |  |
|  | **omega** | -0.03 | 0.15 | -0.32 | -0.12 | -0.03 | 0.07 | 0.27 |  |
|  | **tau** | 0.13 | 0.05 | 0.03 | 0.09 | 0.13 | 0.16 | 0.24 |  |
|  | **tau_**  **consistency** | 0.12 | 0.05 | 0.02 | 0.08 | 0.12 | 0.15 | 0.22 |  |

**Appendix 6: Subgroup Analysis**

**Appendix 6.1:** Handgrip strength

**Figure S6.1.1**: Pairwise Forest Plot for Handgrip Strength


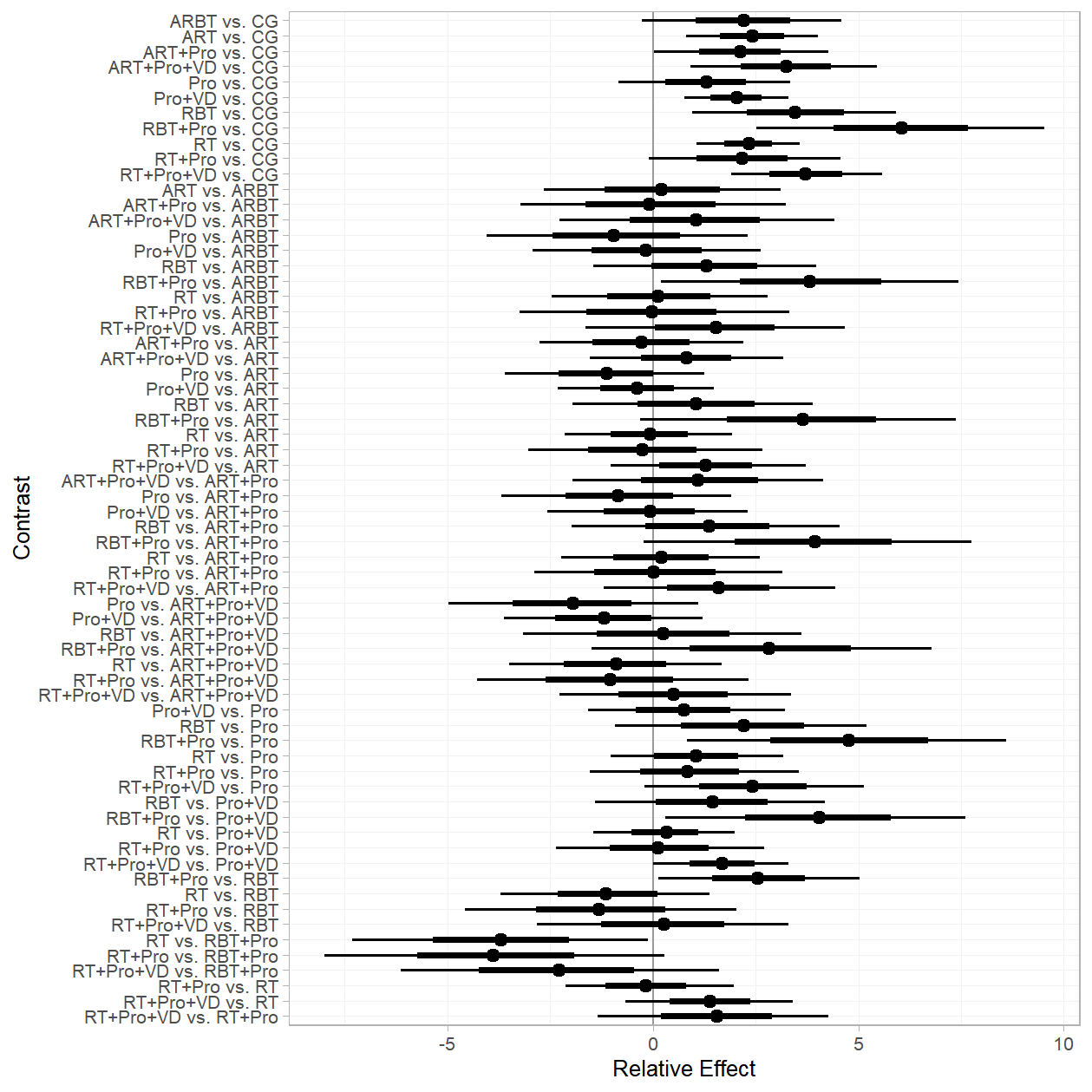


**Appendix 6.2:** Gait speed

**Figure S6.2.3**: Pairwise Forest Plot for Gait speed


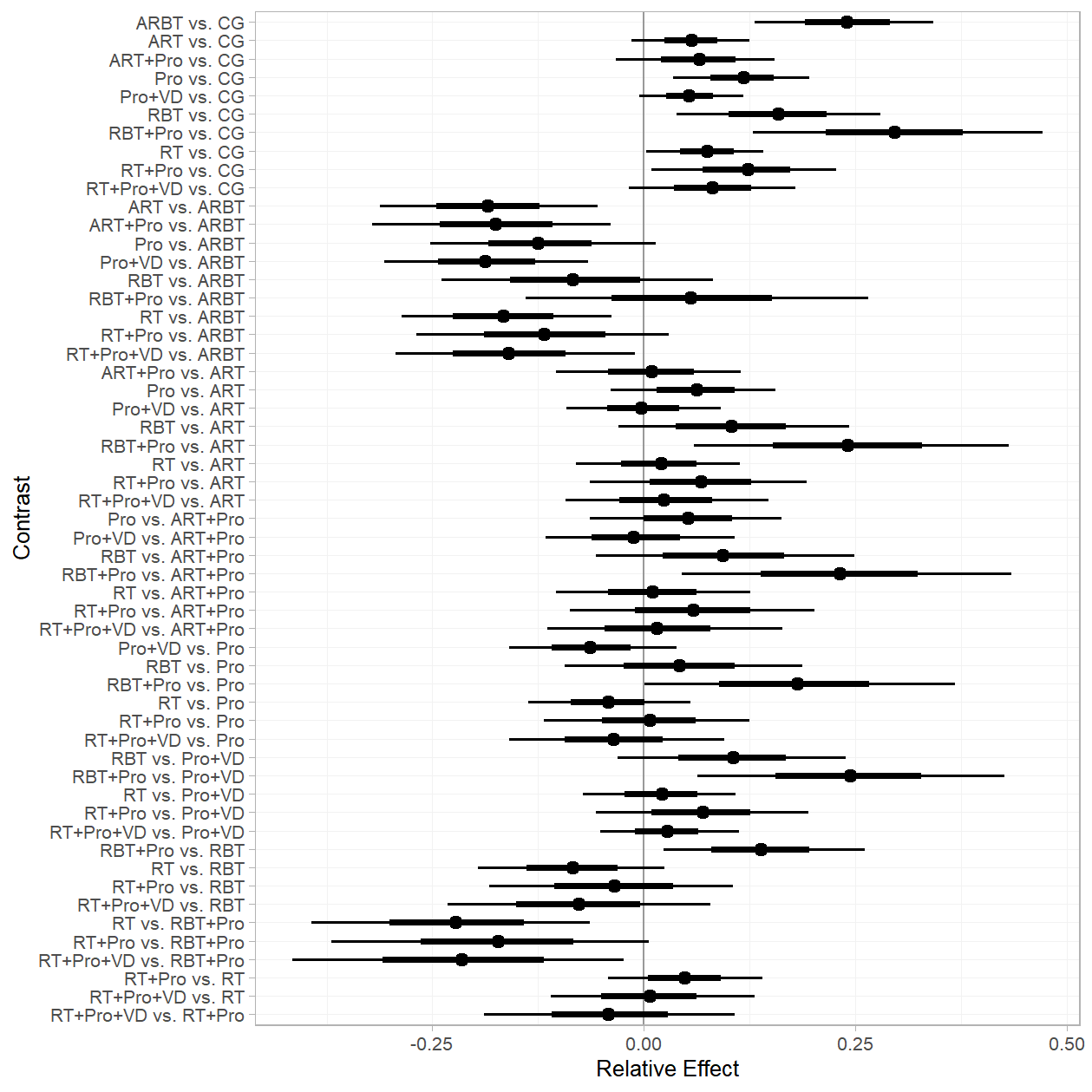


**Appendix 6.3:** ASMI

**Figure S6.3.3**: Pairwise Forest Plot for ASMI


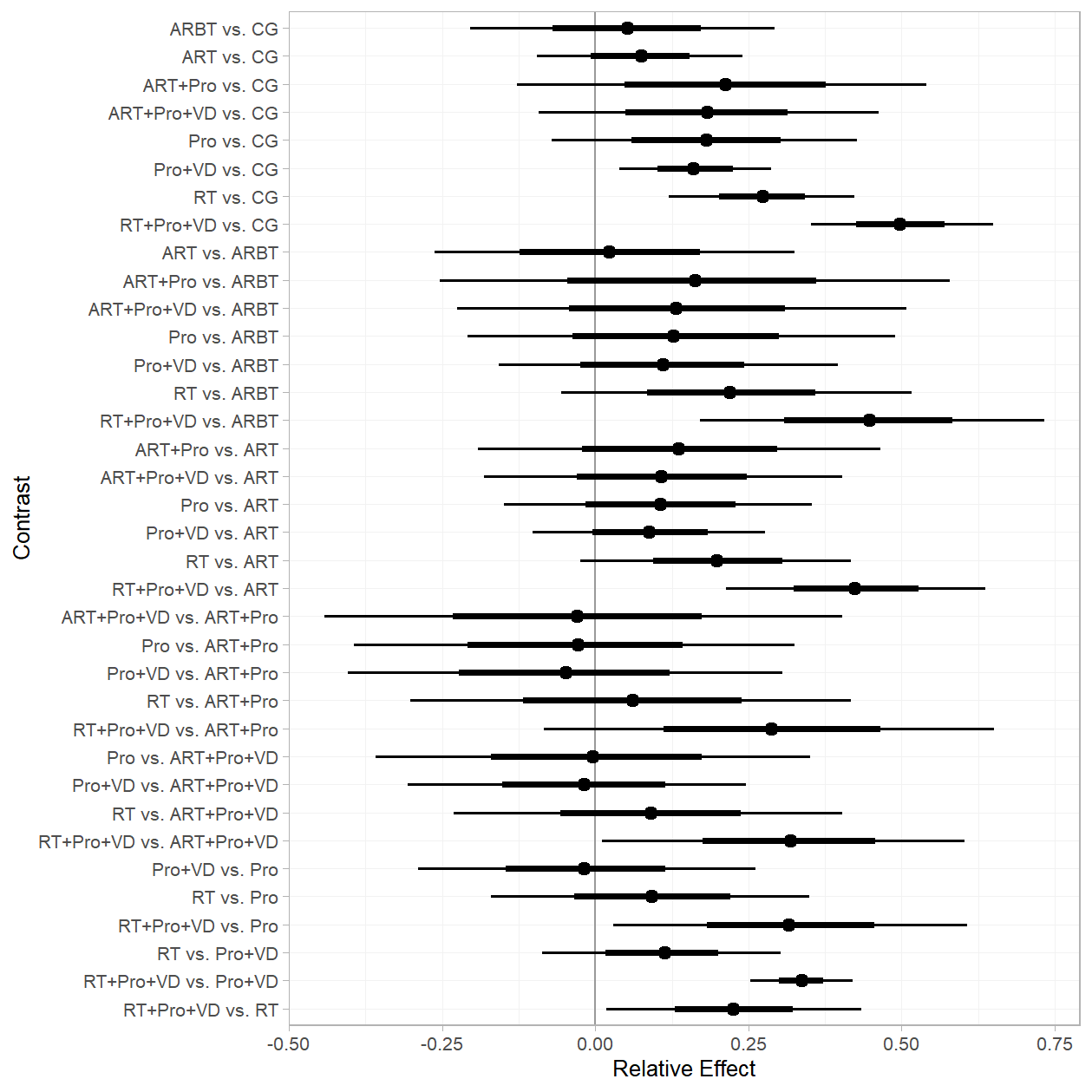


# Appendix 7: SUCRA and cumulative probability plots

**Figure S7.1:** Cumulative Ranking Plot for Handgrip Strength

**
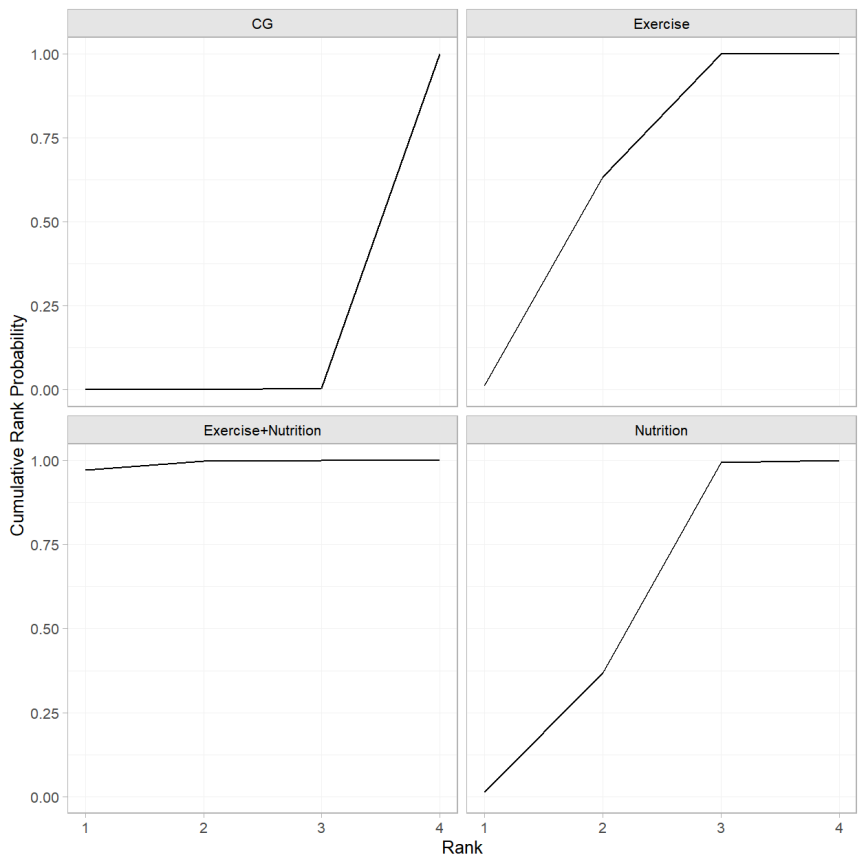
**

**Table S7.1:** SUCRA and Rank Probabilities for Handgrip Strength

| Treatment | Rank 1 | Rank 2 | Rank 3 | Rank 4 | SUCRA |
| --- | --- | --- | --- | --- | --- |
| CG | 0.00000 | 0.00004 | 0.00470 | 0.99526 | 0.16 |
| Exercise | 0.01423 | 0.61738 | 0.36818 | 0.00023 | 54.85 |
| Exercise+Nutrition | 0.97315 | 0.02491 | 0.00194 | 0.00000 | 99.04 |
| Nutrition | 0.01263 | 0.35768 | 0.62519 | 0.00451 | 45.95 |

**Figure S7.2:** Cumulative Ranking Plot for Gait Speed


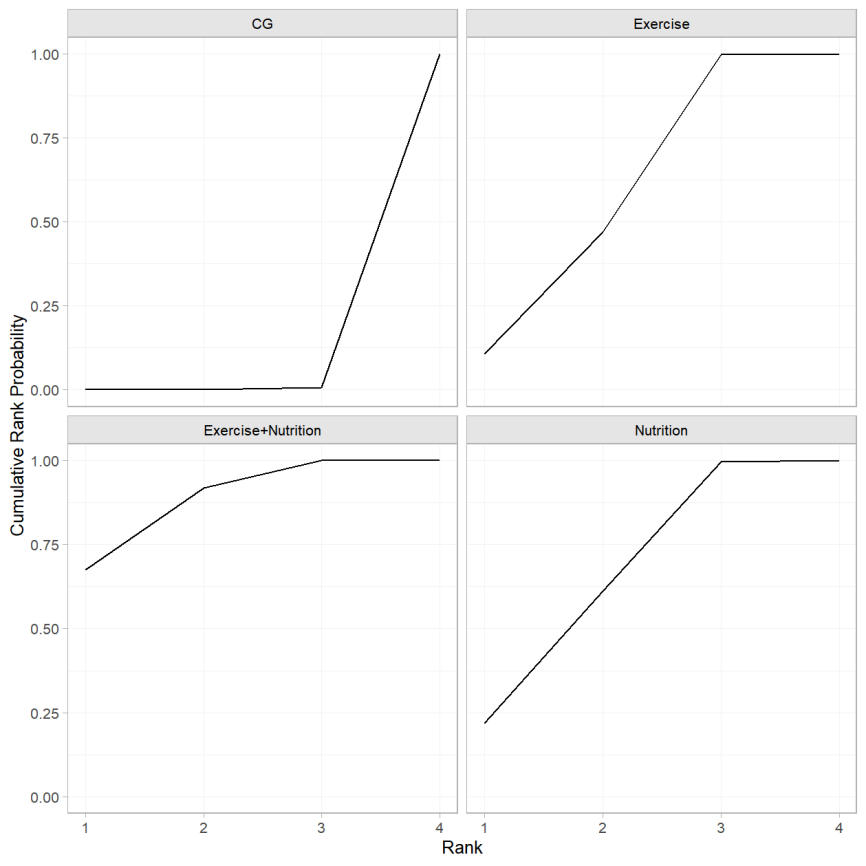


**Table S7.2:** SUCRA and Rank Probabilities for Gait Speed

| Treatment | Rank 1 | Rank 2 | Rank 3 | Rank 4 | SUCRA |
| --- | --- | --- | --- | --- | --- |
| CG | 0.0000 | 0.0002 | 0.0059 | 0.9939 | 0.21 |
| Exercise | 0.10933 | 0.3539 | 0.5344 | 0.0023 | 52.34 |
| Exercise+Nutrition | 0.69028 | 0.2336 | 0.0756 | 0.0006 | 87.12 |
| Nutrition | 0.20039 | 0.4124 | 0.3841 | 0.0032 | 60.33 |

**Figure S7.3:** Cumulative Ranking Plot for ASMI


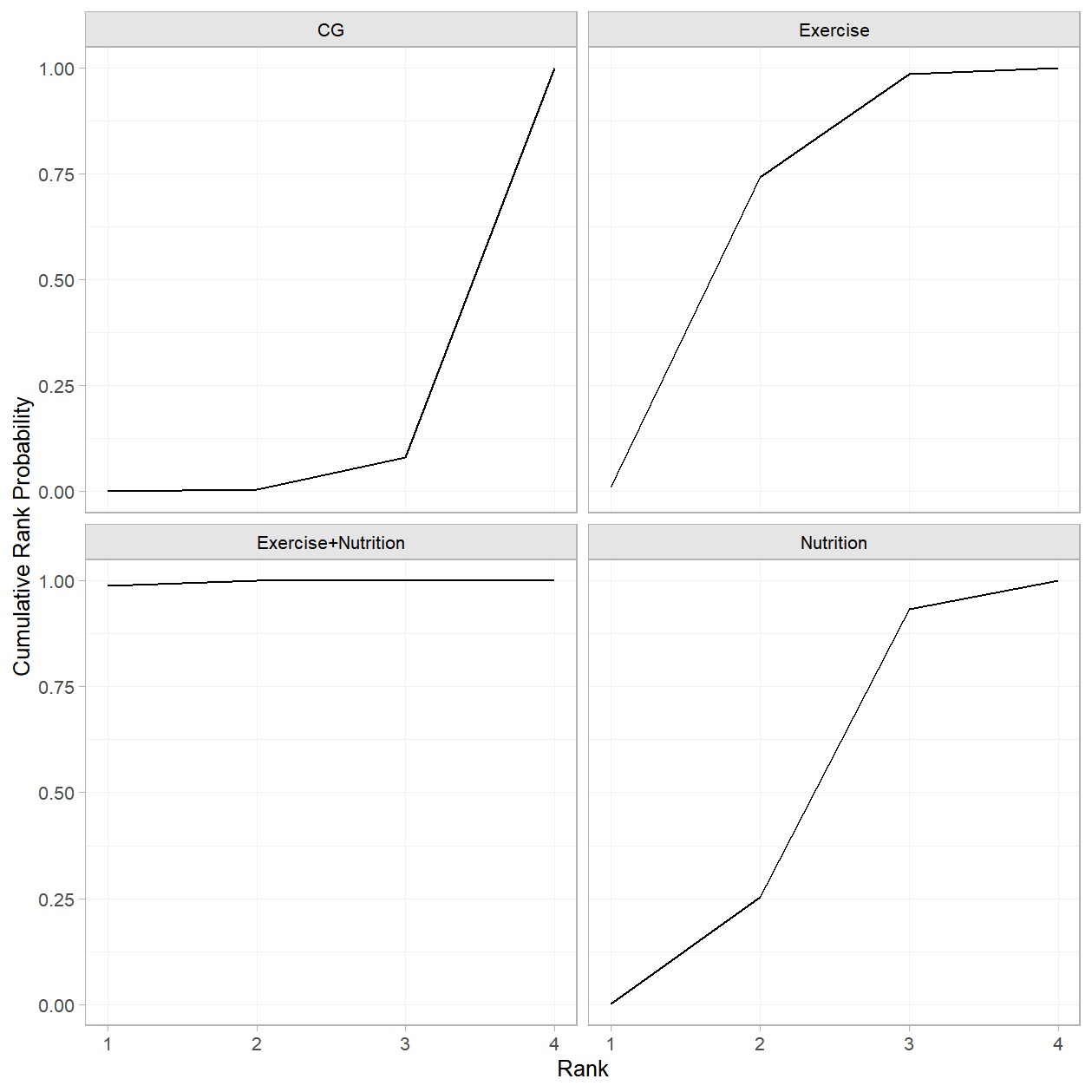


**Figure S7.3:** Cumulative Ranking Plot for ASMI

| Treatment | Rank 1 | Rank 2 | Rank 3 | Rank 4 | SUCRA |
| --- | --- | --- | --- | --- | --- |
| CG | 0.0001 | 0.0032 | 0.0372 | 0.9595 | 1.46 |
| Exercise | 0.0038 | 0.6286 | 0.3533 | 0.0144 | 54.06 |
| Exercise+Nutrition | 0.9951 | 0.0045 | 0.0004 | 0.0000 | 99.82 |
| Nutrition | 0.0011 | 0.3638 | 0.6091 | 0.0261 | 44.66 |

# Appendix 8: CINeMA Assessment

We use the CINeMA framework to assess evidence certainty, evaluating each network estimate based on the following criteria:

- **Within study bias:** We classified the overall risk of bias for each study as low risk of bias, the risk of bias as moderate when none of the four assessed risk of bias items were rated as high risk, and the risk of bias as high when one or both items were rated as high risk. See **Appendix 3** for the bias assessment.
- **Reporting bias:** We judged it visually by a funnel plot **(Appendix 9)**.
- **Indirectness:** Transferability assumptions were assessed by reporting baseline glycated hemoglobin levels in the included study population and by comparing age and BMI at baseline concordance between groups.
- **Imprecision:** We use the CINeMA website to grade the accuracy of each comparison.
- **Heterogeneity:** We assessed the degree of worry by comparing clinical reasoning based on 95% confidence intervals (CIs) while applying the same clinical reasoning framework as for inaccuracy. In particular, we judged the consistency of our findings based on the confidence and prediction intervals associated with clinically important effect sizes. And we used the same thresholds of clinical significance as described above and followed the recommendations automatically provided by CINeMA (https://cinema.ispm.unibe.ch/).
- **Inconsistency:** For inconsistency, we looked at the results for node splitting **(Appendix 5)** and we saw major problems when p<0.10, but otherwise no problems.

**Figure S8.1:** Risk of bias contribution by intervention group in Handgrip strength


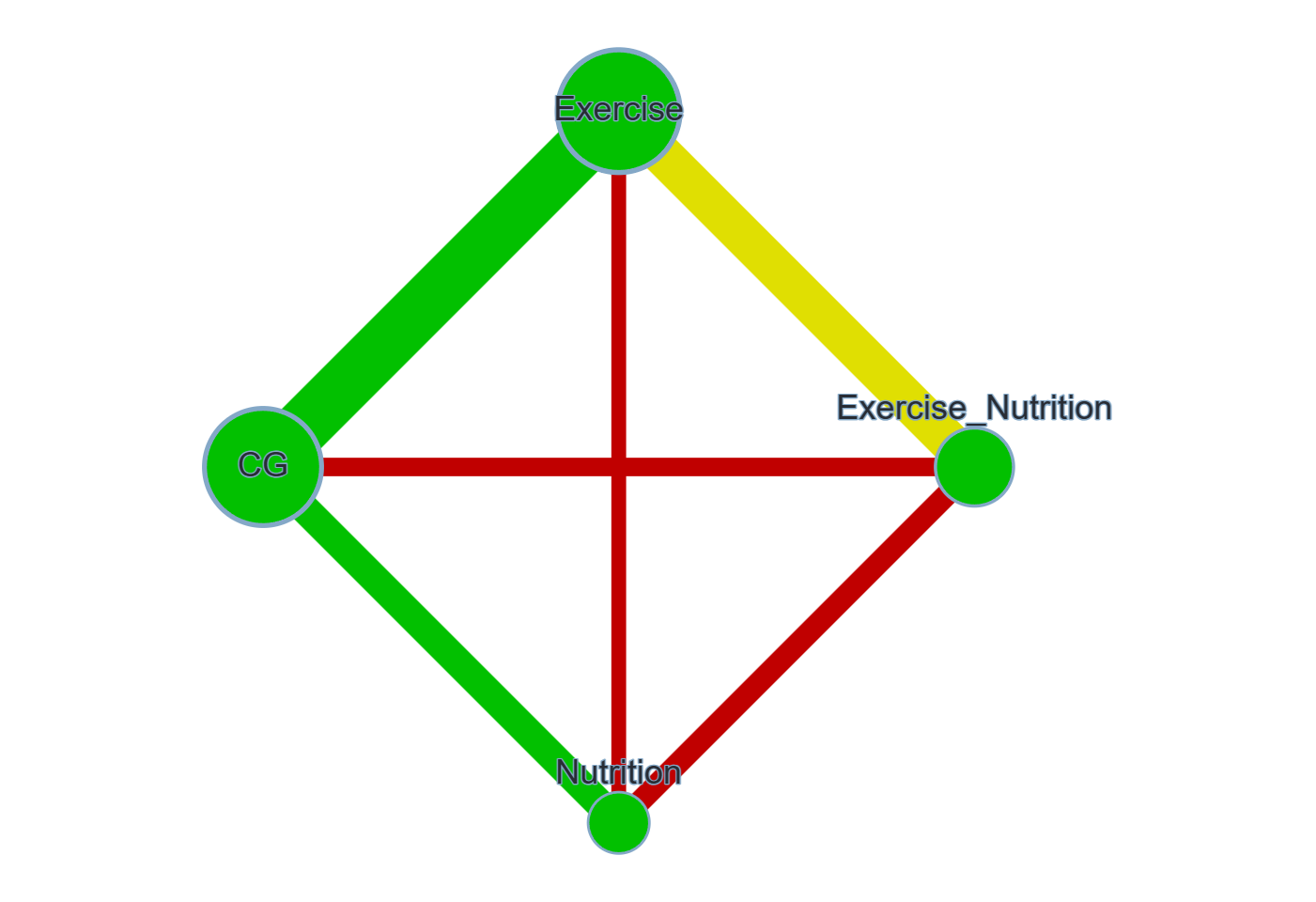


**Figure S8.2:** Overall risk of bias by treatment comparison in Handgrip strength


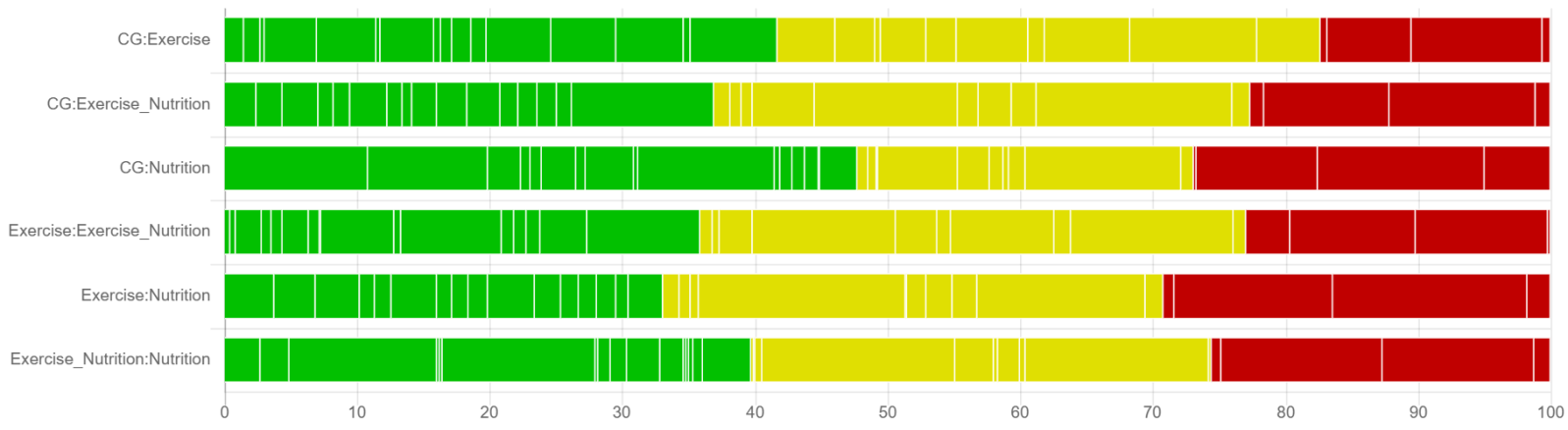


**Table S8.1:** CINeMA Results of Handgrip strength

| Comparison | Number of  studies | Within-  study bias | Reporting bias | Indirectness | Imprecision | Heterogeneity | Incoherence | Confidence rating |
| --- | --- | --- | --- | --- | --- | --- | --- | --- |
| CG:Exercise | 15 | • | • | • | • | • | • | Low |
| CG:Exercise_Nutrition | 5 | • | • | • | • | • | • | Very low |
| CG:Nutrition | 8 | • | • | • | • | • | • | Low |
| Exercise:Exercise_Nutrition | 11 | • | • | • | • | • | • | Very low |
| Exercise:Nutrition | 4 | • | • | • | • | • | • | Very low |
| Exercise_Nutrition:Nutrition | 6 | • | • | • | • | • | • | Low |
| Risk of bias:•Major concerns or high;•,Some concerns or unclear;•,No concerns or low. | | | | | | |  |  |

**Figure S8.3:** Risk of bias contribution by intervention group in Gait speed


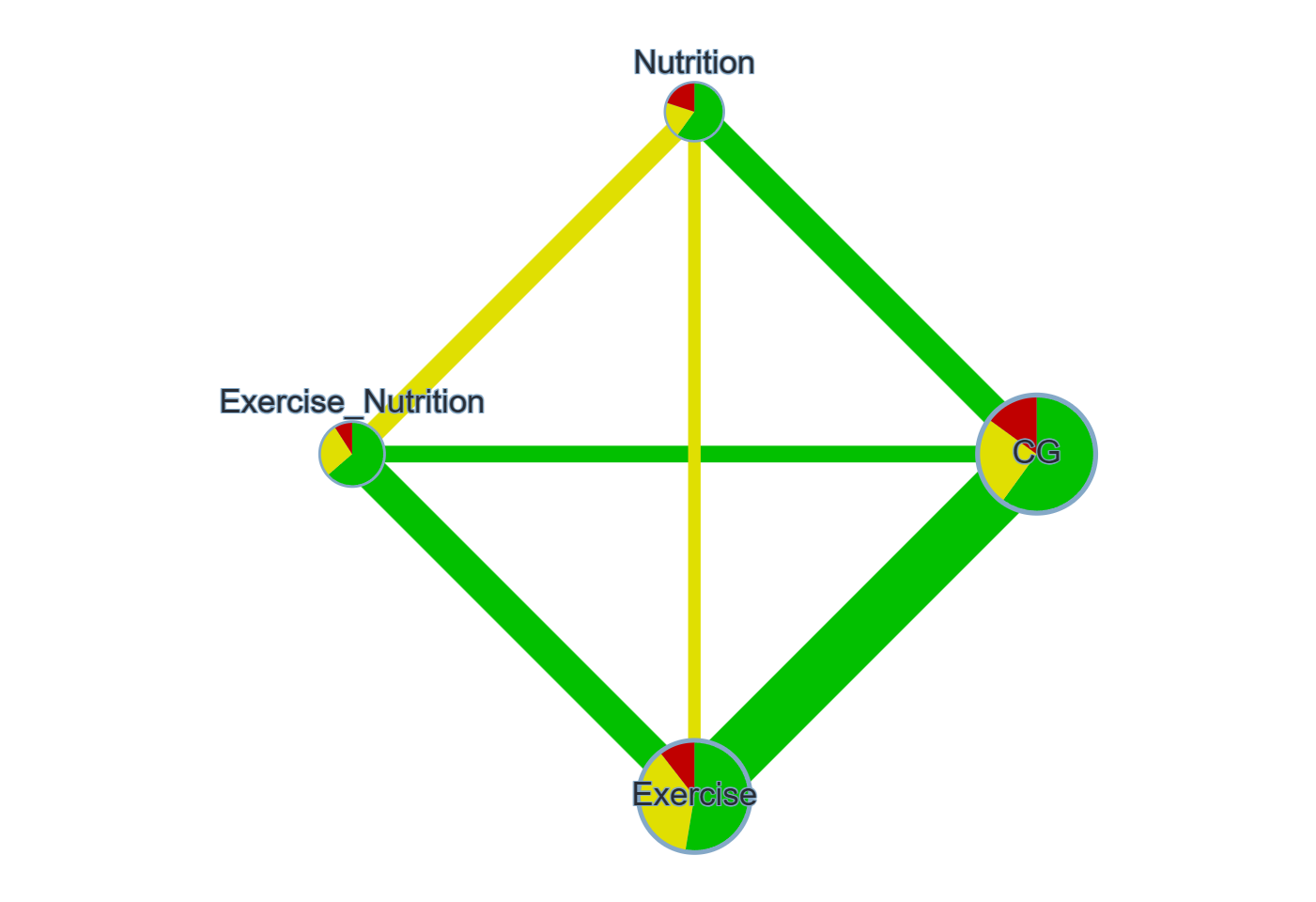


**Figure S8.4:** Overall risk of bias by treatment comparison in Gait speed


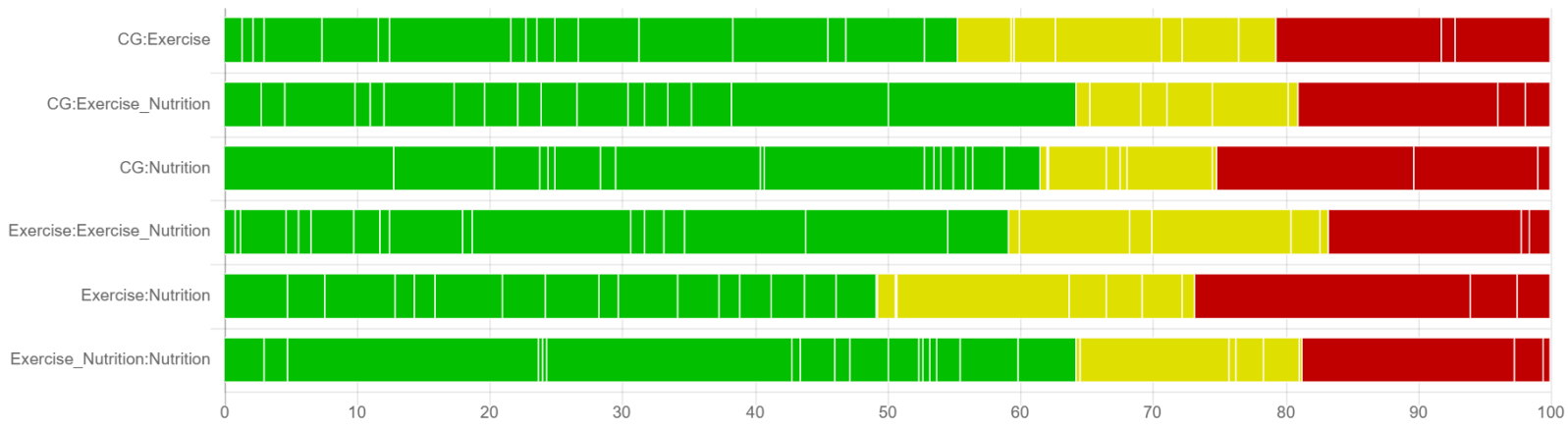


**Table S8.2:** CINeMA Results of Grip speed

| Comparison | Number of  studies | Within-  study bias | Reporting bias | Indirectness | Imprecision | Heterogeneity | Incoherence | Confidence rating |
| --- | --- | --- | --- | --- | --- | --- | --- | --- |
| CG:Exercise | 14 | • | • | • | • | • | • | Low |
| CG:Exercise_Nutrition | 4 | • | • | • | • | • | • | Low |
| CG:Nutrition | 7 | • | • | • | • | • | • | Low |
| Exercise:Exercise_Nutrition | 8 | • | • | • | • | • | • | Low |
| Exercise:Nutrition | 3 | • | • | • | • | • | • | Low |
| Exercise_Nutrition:Nutrition | 5 | • | • | • | • | • | • | Very low |
| Risk of bias:•Major concerns or high;•,Some concerns or unclear;•,No concerns or low. | | | | | | |  |  |

**Figure S8.5:** Risk of bias contribution by intervention group in ASMI


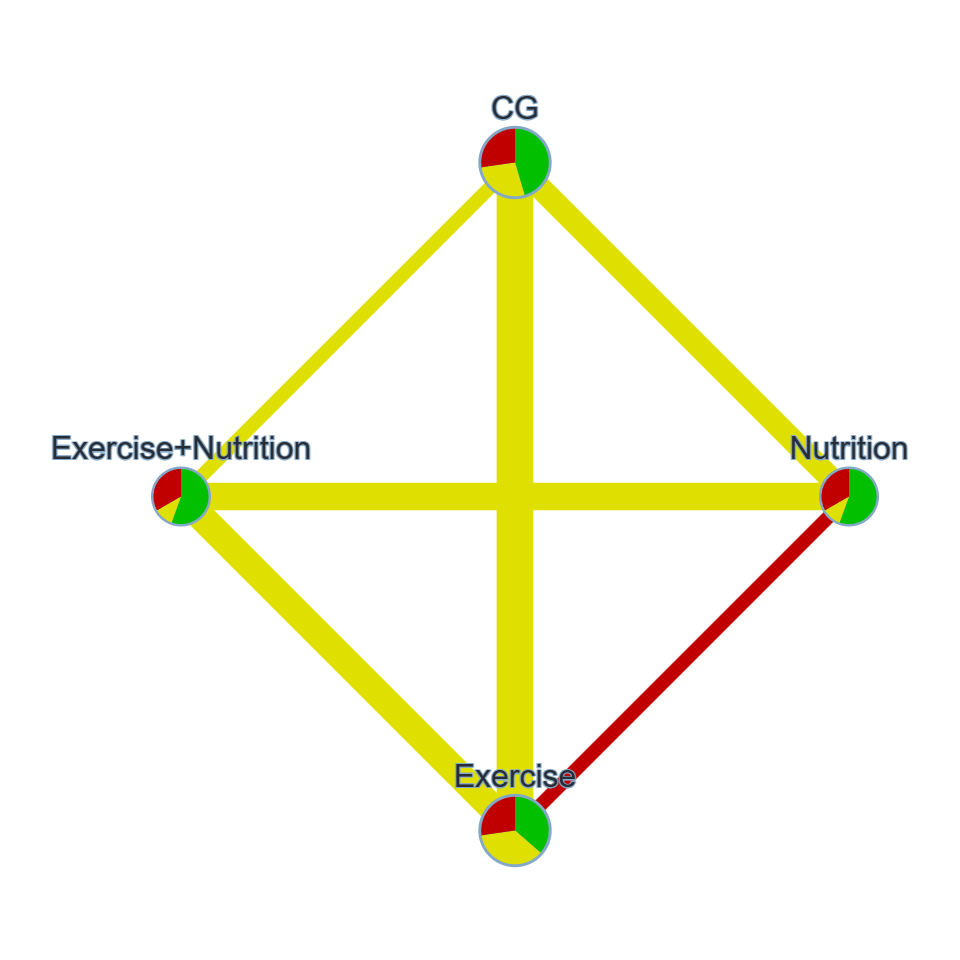


**Figure S8.6:** Overall risk of bias by treatment comparison in ASMI


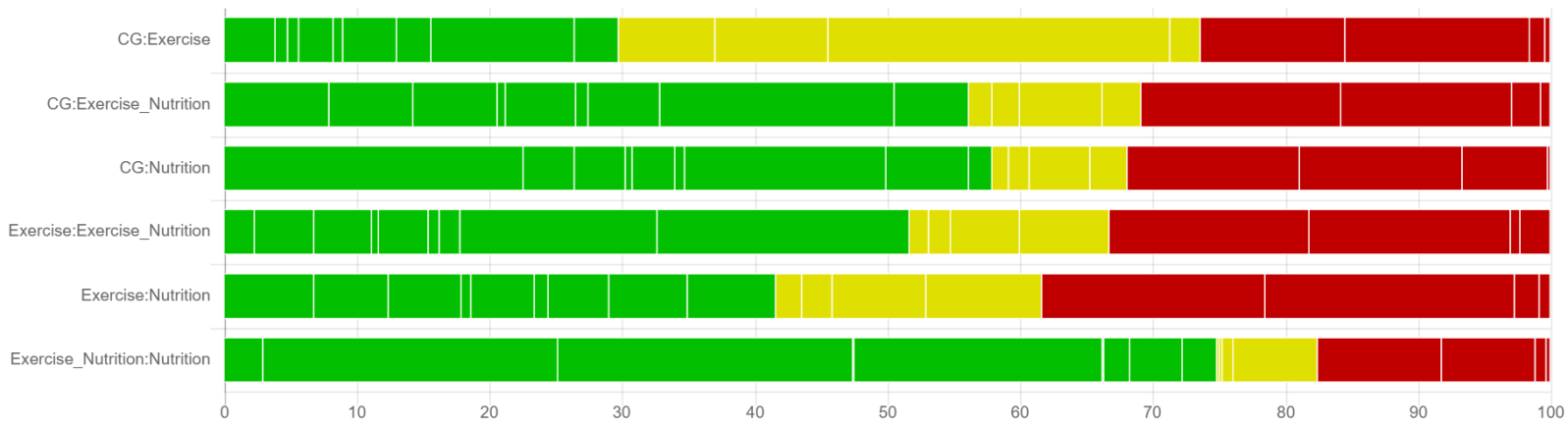


**Table S8.3:** CINeMA Results of ASMI

| Comparison | Number of  studies | Within-  study bias | Reporting bias | Indirectness | Imprecision | Heterogeneity | Incoherence | Confidence rating |
| --- | --- | --- | --- | --- | --- | --- | --- | --- |
| CG:Exercise | 8 | • | • | • | • | • | • | Very low |
| CG:Exercise_Nutrition | 3 | • | • | • | • | • | • | Low |
| CG:Nutrition | 5 | • | • | • | • | • | • | Low |
| Exercise:Exercise_Nutrition | 6 | • | • | • | • | • | • | High |
| Exercise:Nutrition | 3 | • | • | • | • | • | • | Low |
| Exercise_Nutrition:Nutrition | 6 | • | • | • | • | • | • | High |
| Risk of bias:•Major concerns or high;•,Some concerns or unclear;•,No concerns or low. | | | | | | |  |  |

# Appendix 9: Funnel plots

**Figure S9.1:** Funnel plot of Handgrip strength


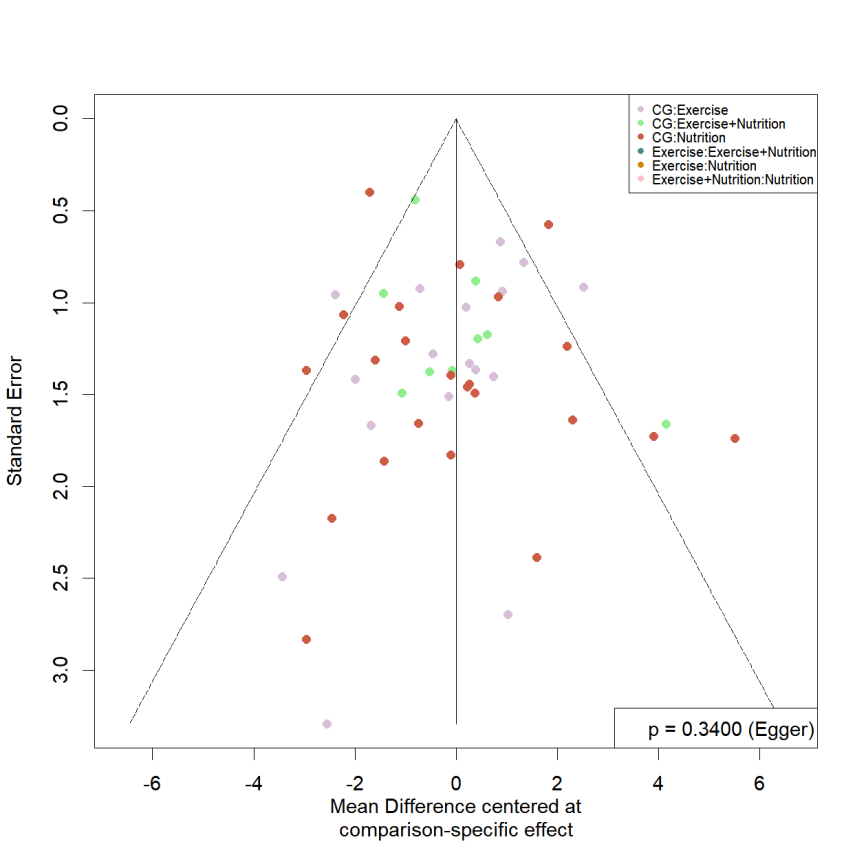


**Figure S9.2:** Funnel plot of Gait speed


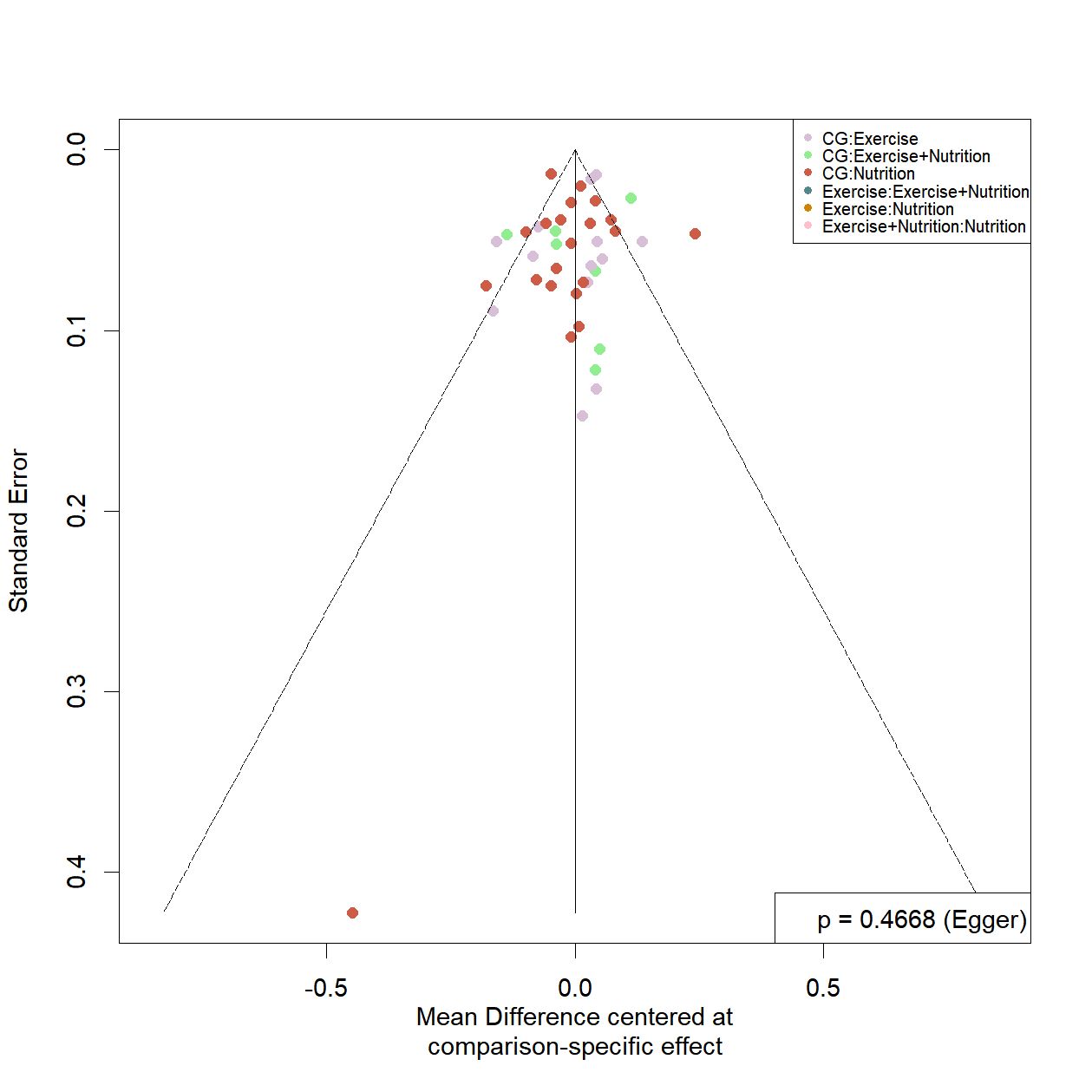


**Figure S9.3:** Funnel plot of ASMI


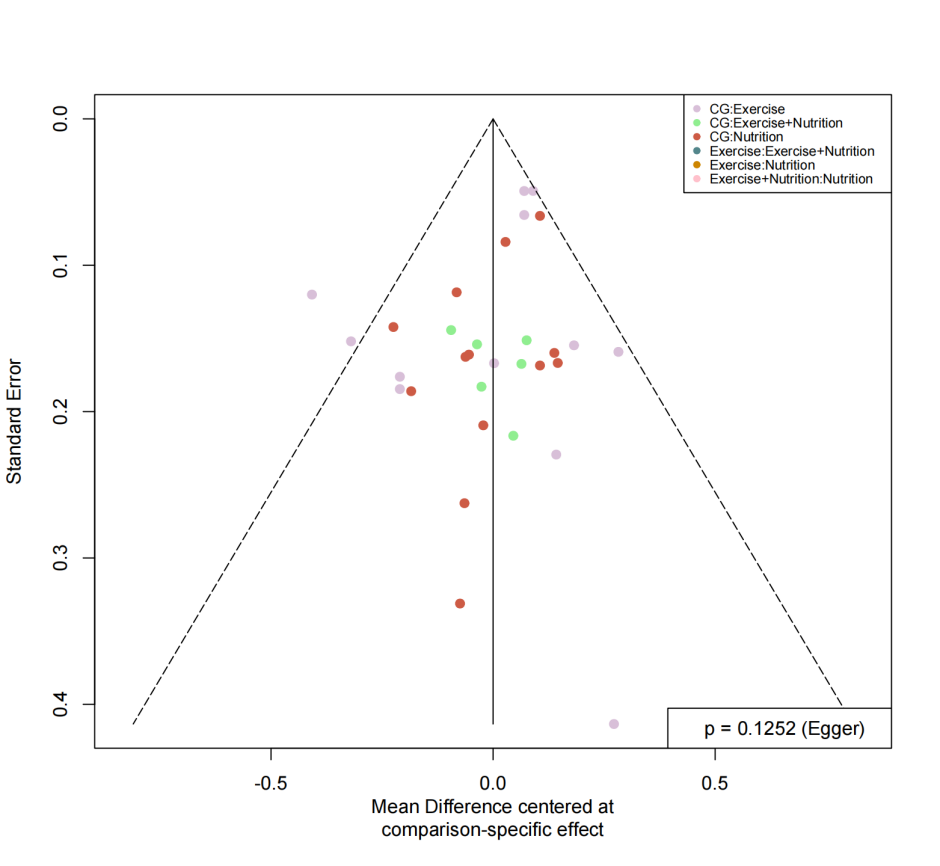


# Appendix10: Network Meta-Regression

**Table S10.1 Outcome of Network Meta-Regression**

| Outcome | Covariate | Covariate | β | Lower | Upper | Median |
| --- | --- | --- | --- | --- | --- | --- |
| Handgrip  strength | Mean Age | Exercise | -1.05 | -3.27 | 1.2 | 74.59 |
|  |  | Exercise+Nutrition | -0.29 | -3.07 | 2.44 |  |
|  |  | Nutrition | -2.34 | -5.87 | 1.17 |  |
|  | Period | Exercise | 1.48 | -2.16 | 5.11 | 16.8 |
|  |  | Exercise+Nutrition | 0.16 | -4.13 | 4.4 |  |
|  |  | Nutrition | 0.22 | -4.44 | 4.02 |  |
|  | Simple size | Exercise | -0.47 | -4.43 | 3.54 | 28.93 |
|  |  | Exercise+Nutrition | -0.60 | -4.77 | 3.57 |  |
|  |  | Nutrition | -0.44 | -2.26 | 1.45 |  |
|  | Proportion of males | Exercise | -0.70 | -4.44 | 2.96 | 35.05% |
|  |  | Exercise+Nutrition | -0.12 | -4.06 | 3.77 |  |
|  |  | Nutrition | -0.75 | -4.87 | 3.33 |  |
|  | BMI | Exercise | 0.38 | -2.59 | 3.31 | 23.3 |
|  |  | Exercise+Nutrition | 0.52 | -3.37 | 4.33 |  |
|  |  | Nutrition | -2.03 | -5.76 | 1.62 |  |
|  | Handgrip strength | Exercise | 0.36 | -4.16 | 4.87 | 19.69 |
|  |  | Exercise+Nutrition | -0.49 | -4.46 | 3.45 |  |
|  |  | Nutrition | -1.43 | -5.23 | 2.32 |  |
|  | ASMI | Exercise | 0.44 | -0.57 | 3.49 | 6.16 |
|  |  | Exercise+Nutrition | -1.55 | -2.82 | 2.26 |  |
|  |  | Nutrition | -1.61 | -2.83 | 1.99 |  |
| Gait speed | Mean Age | Exercise | -0.013 | -0.113 | 0.09 | 76.26 |
|  |  | Exercise+Nutrition | 0.018 | -0.1058 | 0.147 |  |
|  |  | Nutrition | -0.017 | -0.168 | 0.136 |  |
|  | Period | Exercise | -0.012 | -0.184 | 0.162 | 17.03 |
|  |  | Exercise+Nutrition | -0.057 | -0.238 | 0.11 |  |
|  |  | Nutrition | -0.052 | -0.236 | 0.118 |  |
|  | Simple size | Exercise | 0.045 | -0.093 | 0.184 | 31 |
|  |  | Exercise+Nutrition | 0.078 | -0.683 | 0.227 |  |
|  |  | Nutrition | 0.018 | -0.099 | 0.134 |  |
|  | Proportion of males | Exercise | -0.045 | -0.234 | 0.143 | 36.26% |
|  |  | Exercise+Nutrition | -0.043 | -0.218 | 0.13 |  |
|  |  | Nutrition | -0.046 | -0.236 | 0.147 |  |
|  | BMI | Exercise | -0.046 | -0.159 | 0.066 | 23.17 |
|  |  | Exercise+Nutrition | 0.063 | -0.117 | 0.246 |  |
|  |  | Nutrition | 0.006 | -0.105 | 0.118 |  |
|  | Handgrip strength | Exercise | -0.071 | -0.248 | 0.107 | 19.56 |
|  |  | Exercise+Nutrition | -0.066 | -0.199 | 0.069 |  |
|  |  | Nutrition | -0.054 | -0.190 | 0.088 |  |
|  | ASMI | Exercise | 0.045 | -0.093 | 0.184 | 6.11 |
|  |  | Exercise+Nutrition | 0.078 | -0.068 | 0.227 |  |
|  |  | Nutrition | 0.018 | -0.099 | 0.134 |  |
| ASMI | Mean Age | Exercise | -0.071 | -0.248 | 0.107 | 75.12 |
|  |  | Exercise+Nutrition | -0.066 | -0.199 | 0.069 |  |
|  |  | Nutrition | -0.054 | -0.190 | 0.088 |  |
|  | Period | Exercise | 0.111 | -0.460 | 0.650 | 20.85 |
|  |  | Exercise+Nutrition | 0.206 | -0.218 | 0.632 |  |
|  |  | Nutrition | 0.118 | -0.318 | 0.548 |  |
|  | Simple size | Exercise | -0.022 | -0.239 | 0.197 | 27.71 |
|  |  | Exercise+Nutrition | -0.242 | -0.536 | 0.064 |  |
|  |  | Nutrition | 0.027 | -0.265 | 0.327 |  |
|  | Proportion of males | Exercise | -0.003 | -0.441 | 0.425 | 48.74% |
|  |  | Exercise+Nutrition | -0.065 | -0.433 | 0.307 |  |
|  |  | Nutrition | -0.233 | -0.656 | 0.186 |  |
|  | BMI | Exercise | 0.183 | -0.204 | 0.566 | 22.68 |
|  |  | Exercise+Nutrition | 0.163 | -0.234 | 0.563 |  |
|  |  | Nutrition | -0.08 | 0.5 | 0.318 |  |
|  | Handgrip strength | Exercise | 0.033 | -0.528 | 0.612 | 21.62 |
|  |  | Exercise+Nutrition | 0.111 | -0.256 | 0.485 |  |
|  |  | Nutrition | -0.012 | -0.373 | 0.346 |  |
|  | ASMI | Exercise | -0.048 | -0.431 | 0.369 | 5.95 |
|  |  | Exercise+Nutrition | -0.048 | -0.379 | 0.302 |  |
|  |  | Nutrition | -0.241 | -0.618 | 0.144 |  |

Crl:credible interval;*:significant influence factors, 95% Crl does not contain zero

# Appendix11: Sensitivity analysis

**Appendix 11.1** Meta-Regression-Based Sensitivity Analysis

**11.1.1** Sensitivity Analysis for Handgrip Strength

**11.1.1.1** Mean Age

When the model was adjusted for centering value of mean age, the hierarchy from the unadjusted model retained.

**Figure S11.1.1.1** presents the impact of various interventions on overall symptom changes after adjusting for the mean age 74.59.


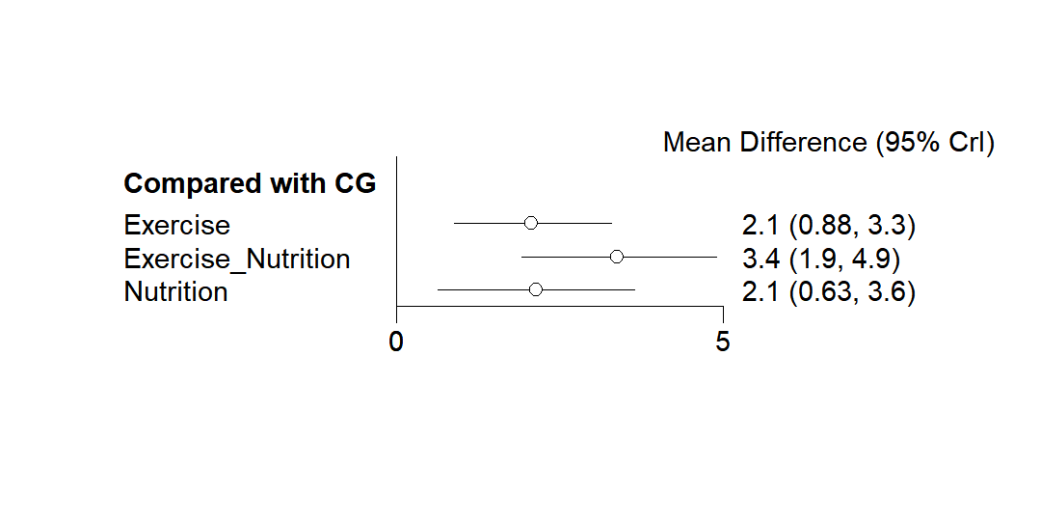


**11.1.1.2** Sample size
When the model was adjusted for centering value of sample size, the hierarchy from the unadjusted model retained.

**Figure S11.1.1.2** presents the impact of various interventions on overall symptom changes after adjusting for the sample size 28.93.


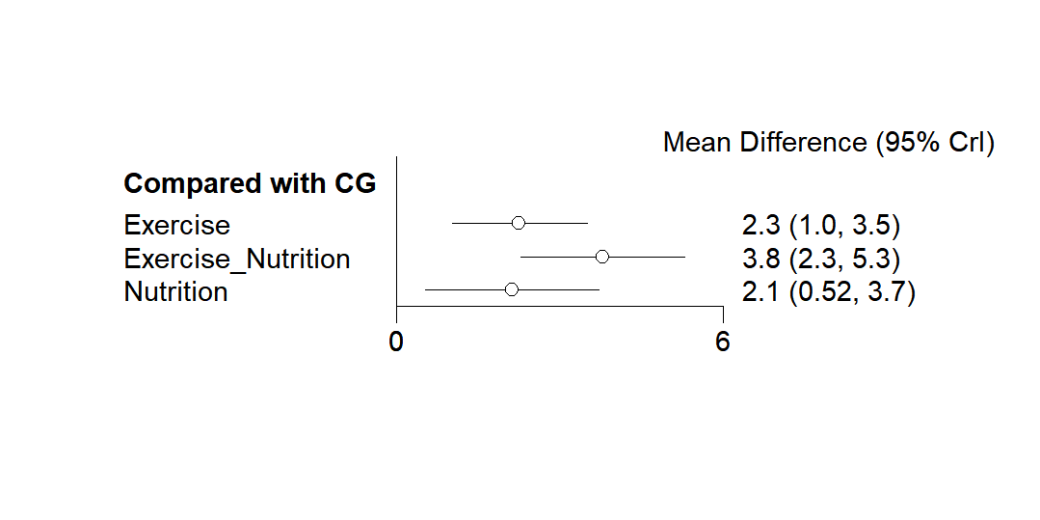


**11.1.1.3** Period
When the model was adjusted for centering value of period, the hierarchy from the unadjusted model retained.

**Figure S11.1.1.3** presents the impact of various interventions on overall symptom changes after adjusting for the period of 16.8.


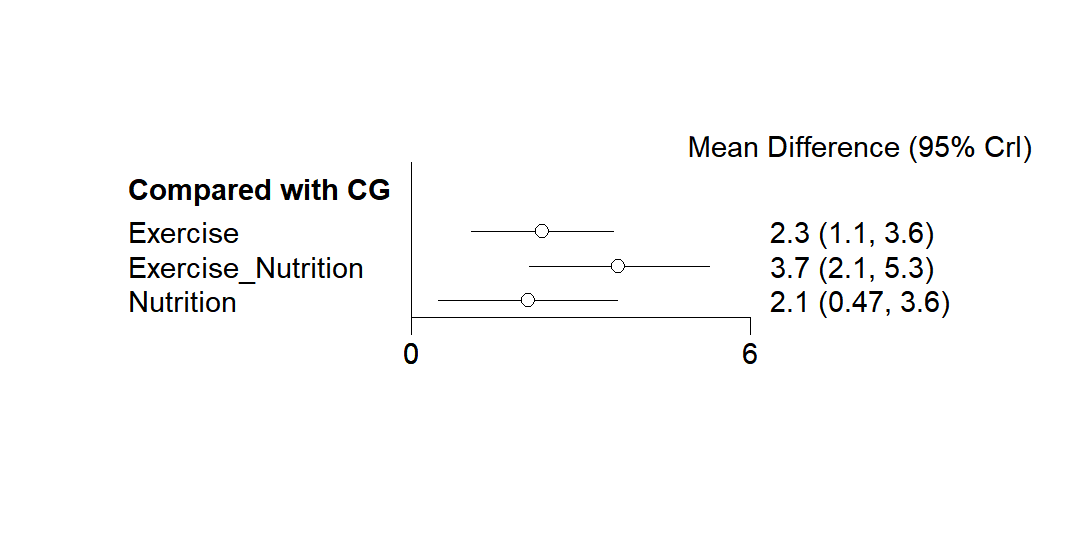


**11.1.1.4** BMI

When the model was adjusted for centering value of BMI, the hierarchy from the unadjusted model retained.

**Figure S11.1.1.4** presents the impact of various interventions on overall symptom changes after adjusting for the BMI of 23.3.


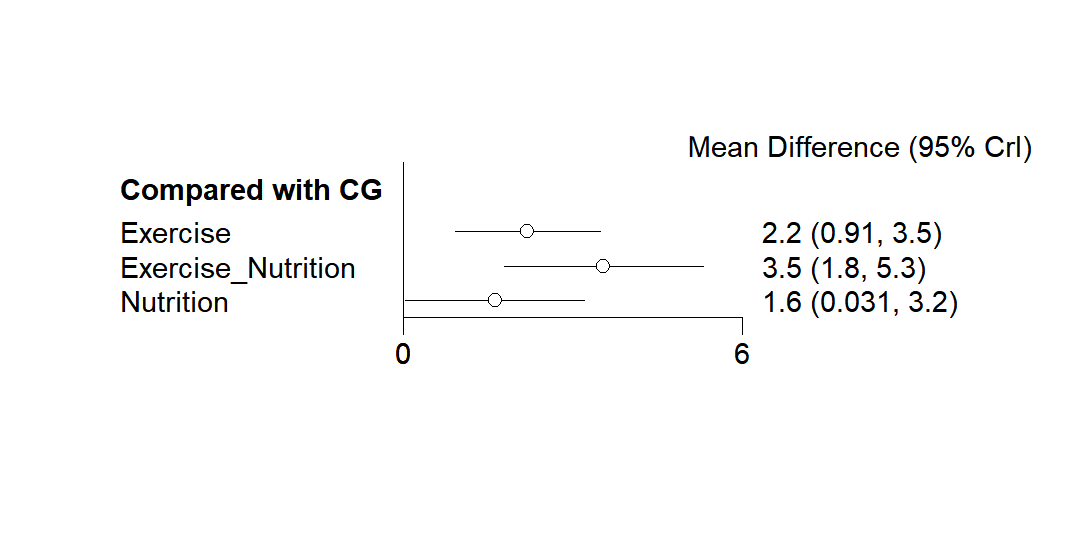


**11.1.1.5** Handgrip strength
When the model was adjusted for centering value of handgrip strength , the hierarchy from the unadjusted model retained.

**Figure S11.1.1.5** presents the impact of various interventions on overall symptom changes after adjusting for handgrip strength of 19.69.


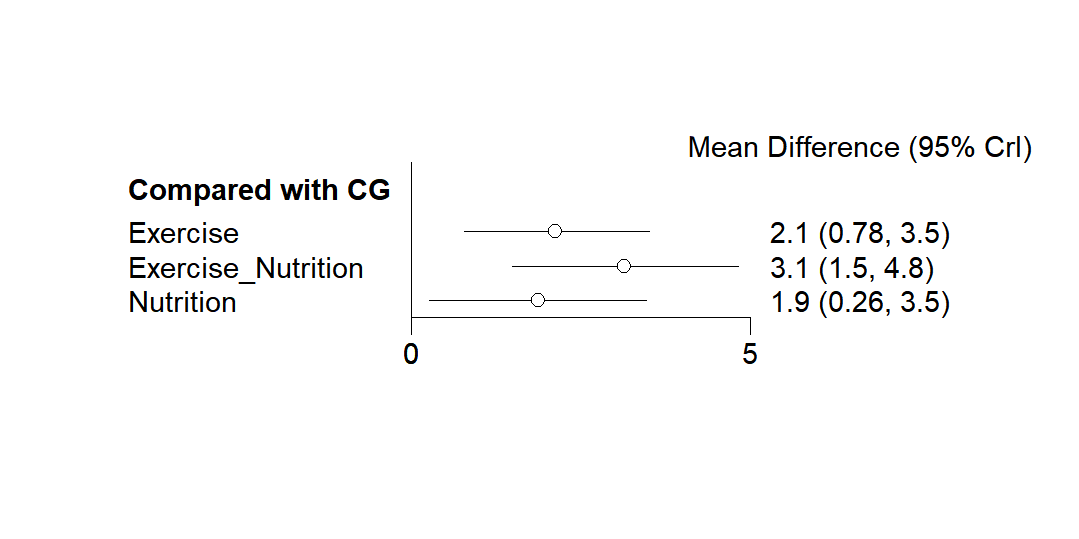


**11.1.1.6** Percentage of Male
When the model was adjusted for centering value of Percentage of Male, the hierarchy from the unadjusted model retained.

**Figure S11.1.1.6** presents the impact of various interventions on overall symptom changes after adjusting for the percentage of male of 35.05%.


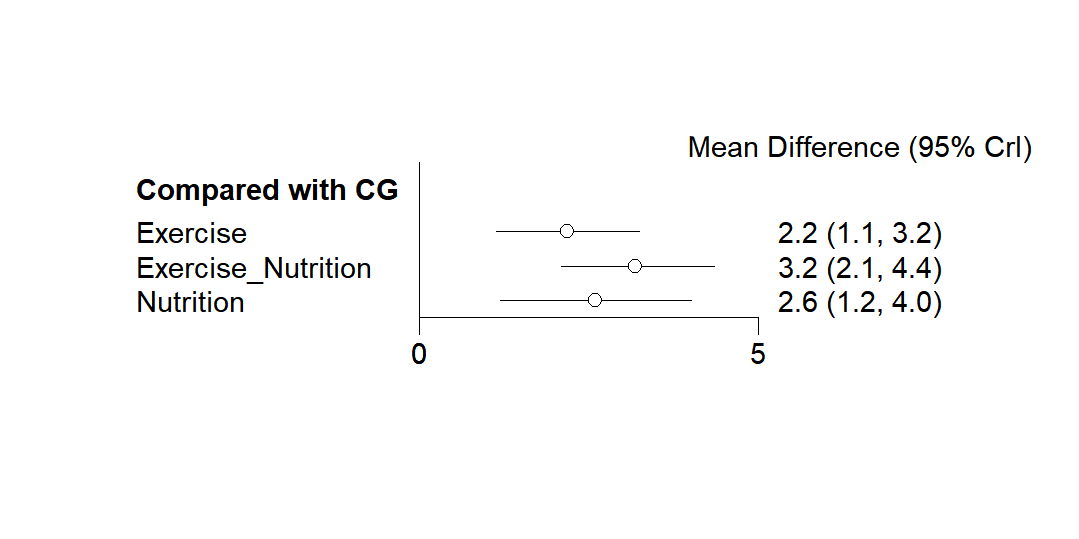


**11.1.1.7** ASMI
When the model was adjusted for centering value of ASMI, the hierarchy from the unadjusted model retained.

**Figure S11.1.1.7** presents the impact of various interventions on overall symptom changes after adjusting for the ASMI of 6.16.


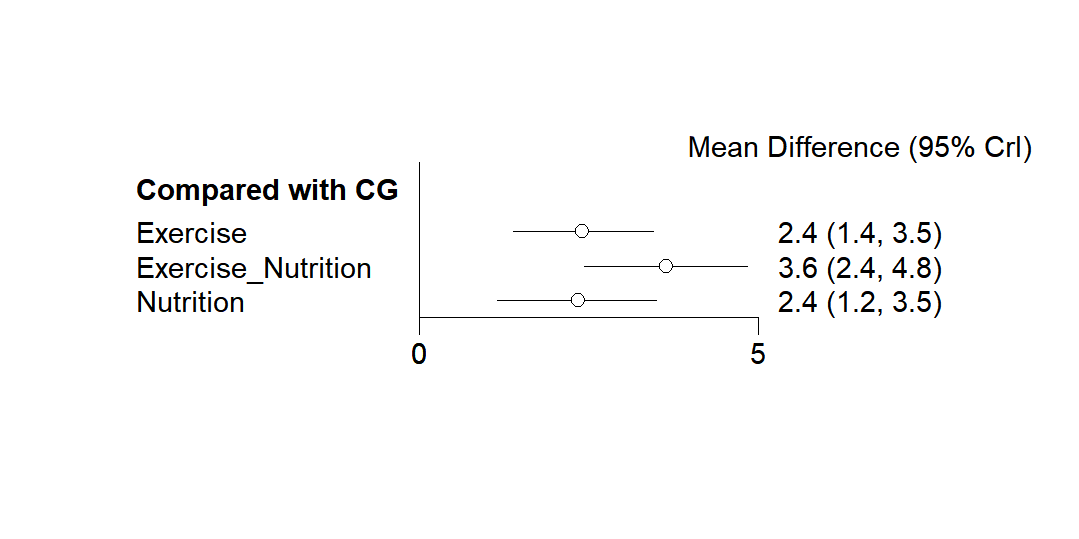


**11.1.2** Sensitivity Analysis for Gait Speed

**11.1.2.1** Mean Age

When the model was adjusted for centering value of mean age, the hierarchy from the unadjusted model retained.

**Figure S11.1.2.1** presents the impact of various interventions on overall symptom changes after adjusting for the mean age 76.26.


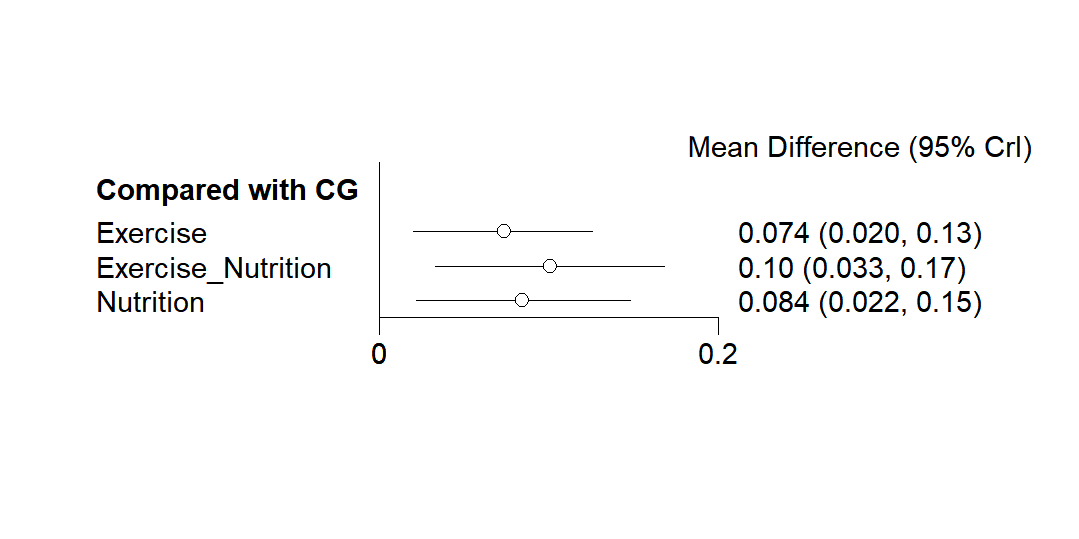


**11.1.2.2** Sample size
When the model was adjusted for centering value of sample size, the hierarchy from the unadjusted model retained.

**Figure S11.1.2.2** presents the impact of various interventions on overall symptom changes after adjusting for the sample size 31.


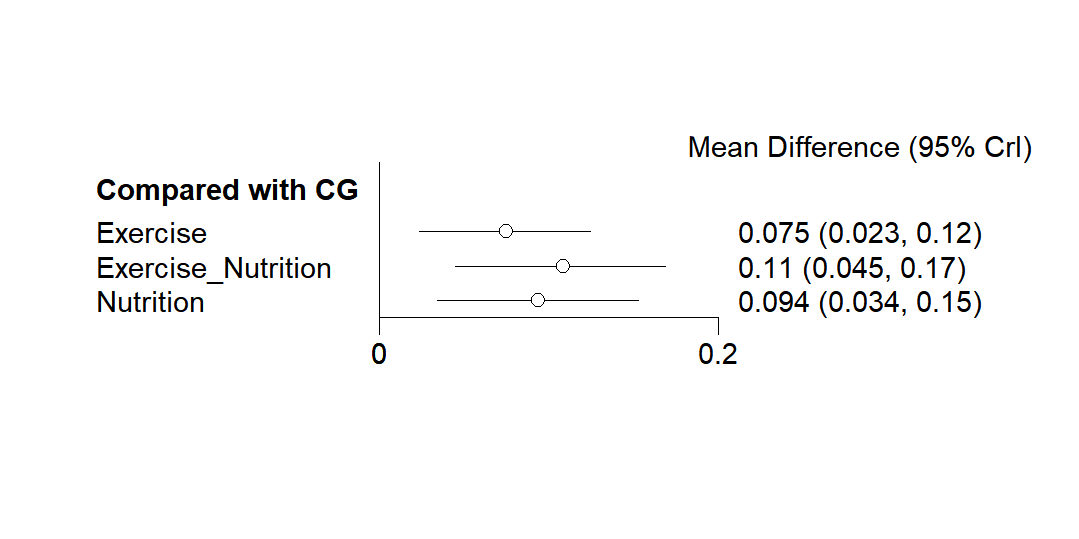


**11.1.2.3** Period
When the model was adjusted for centering value of period, the hierarchy from the unadjusted model retained.

**Figure S11.1.2.3** presents the impact of various interventions on overall symptom changes after adjusting for the period of 17.03.


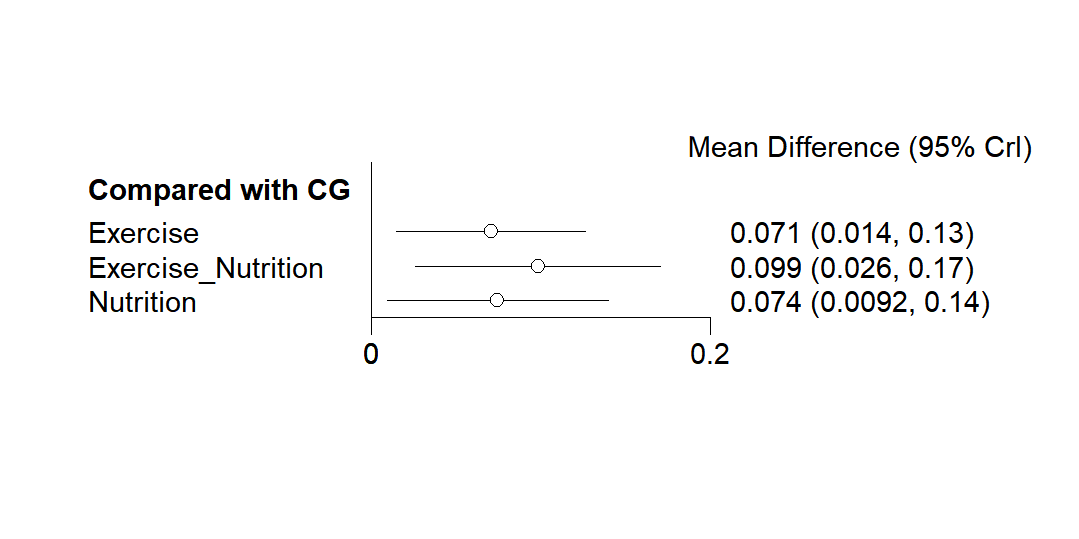


**11.1.2.4** BMI

When the model was adjusted for centering value of BMI, the hierarchy from the unadjusted model retained.

**Figure S11.1.2.4** presents the impact of exercise interventions on overall symptom changes after adjusting for the BMI of 23.17.


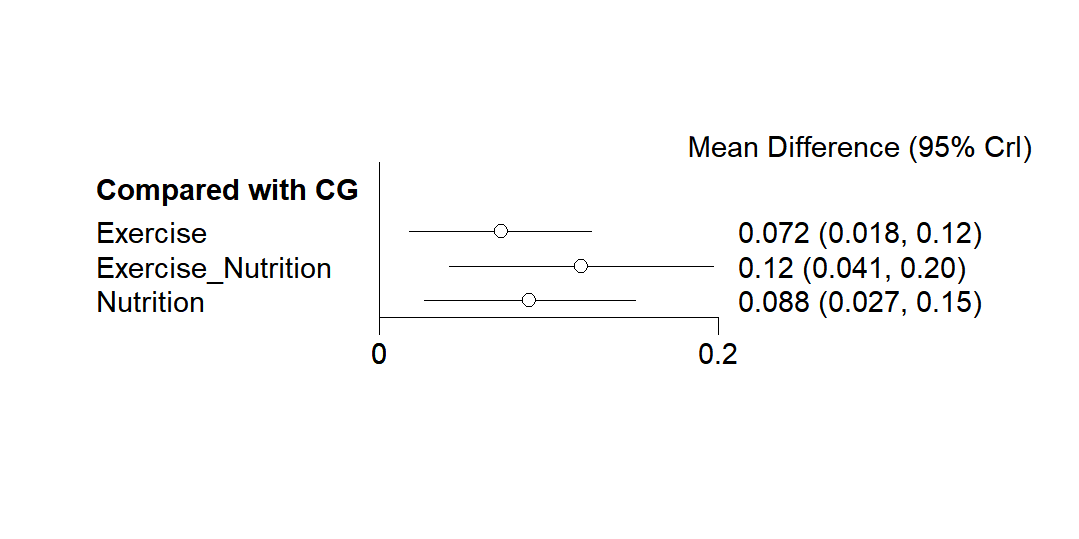


**11.1.2.5** Handgrip strength
When the model was adjusted for centering value of handgrip strength, the hierarchy from the unadjusted model retained.

**Figure S11.1.2.5** presents the impact of various interventions on overall symptom changes after adjusting for handgrip strength of 19.56.


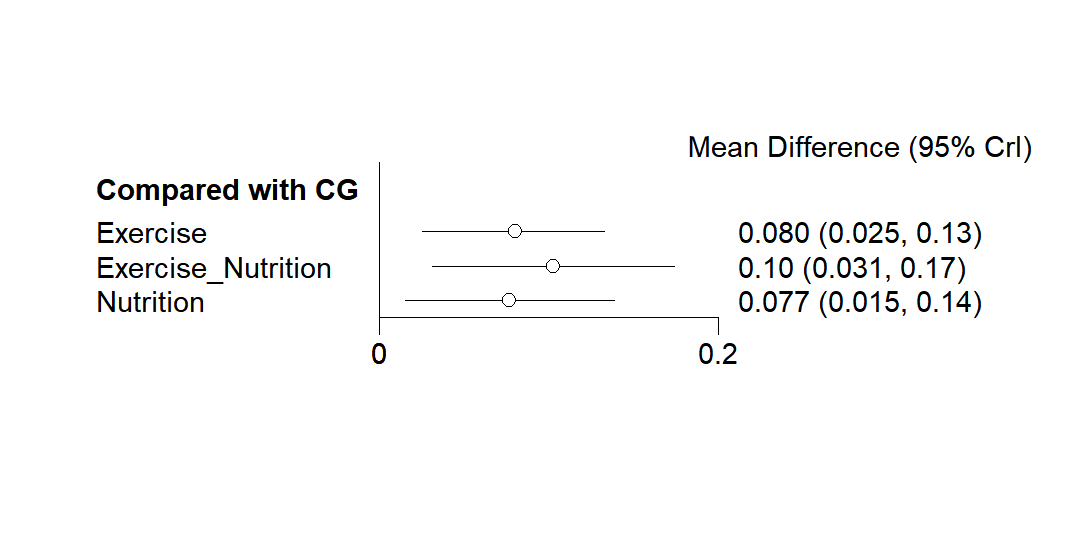


**11.1.2.6** Percentage of Male
When the model was adjusted for centering value of Percentage of Male 36.26%, the hierarchy from the unadjusted model retained.

**Figure S11.1.2.6** presents the impact of various interventions on overall symptom changes after adjusting for the percentage of male of 36.26%.


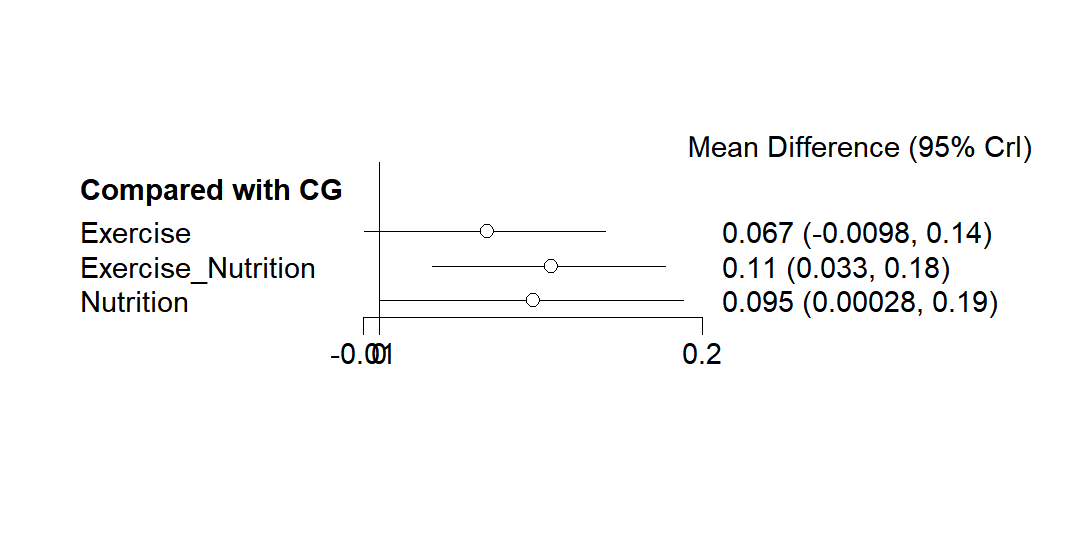


**11.1.2.7** ASMI
When the model was adjusted for centering value of ASMI, the hierarchy from the unadjusted model retained.

**Figure S11.1.2.7** presents the impact of various interventions on overall symptom changes after adjusting for the ASMI of 6.11.


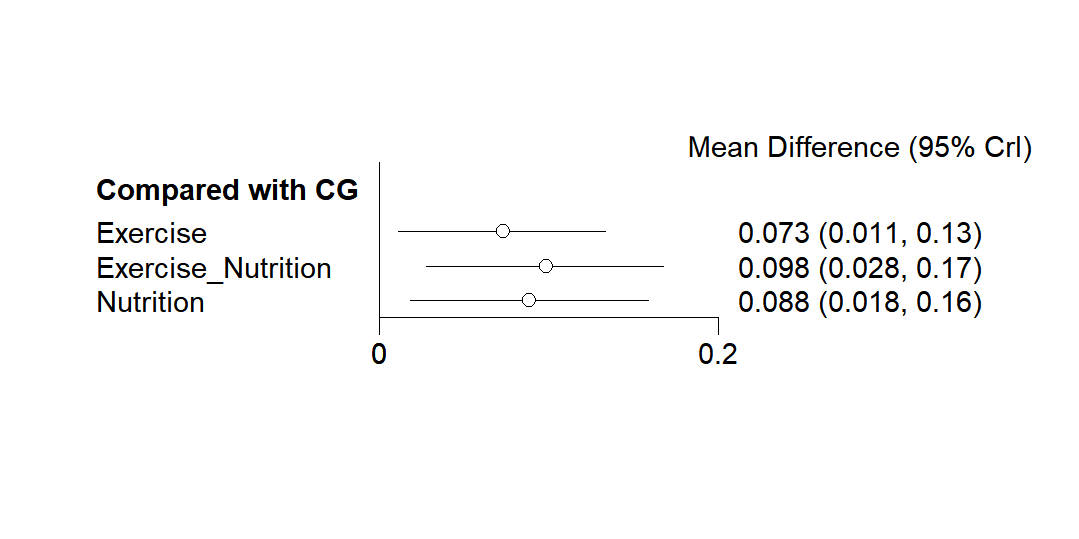


**11.1.3** Sensitivity Analysis for ASMI

**11.1.3.1** Mean Age

When the model was adjusted for centering value of mean age, the hierarchy from the unadjusted model retained.

**Figure S11.1.3.1** presents the impact of various interventions on overall symptom changes after adjusting for the mean age of 75.12.


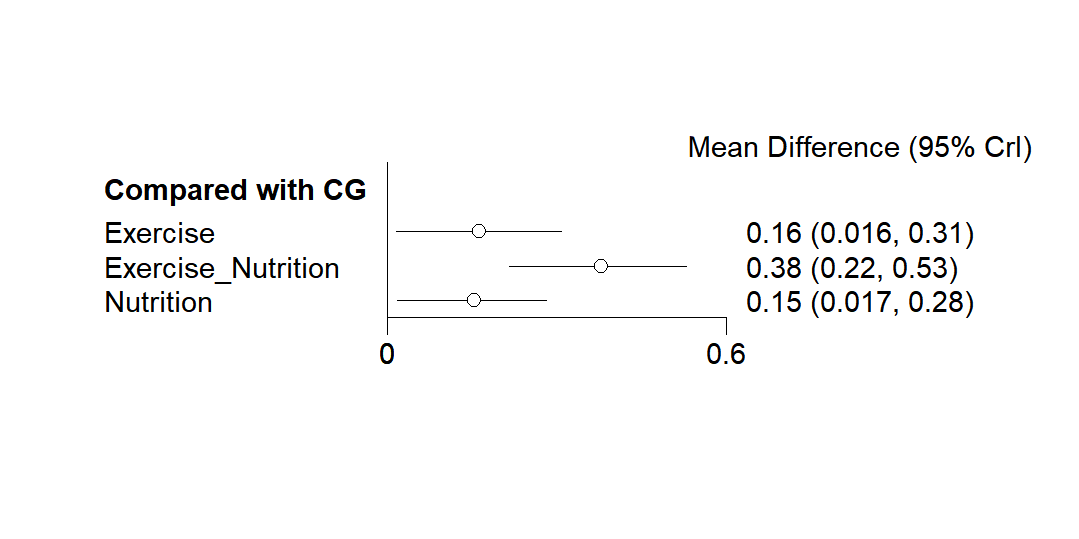


**11.1.3.2** Sample size
When the model was adjusted for centering value of sample size, the hierarchy from the unadjusted model retained.

**Figure S11.1.3.2** presents the impact of various interventions on overall symptom changes after adjusting for the sample size of 27.71.


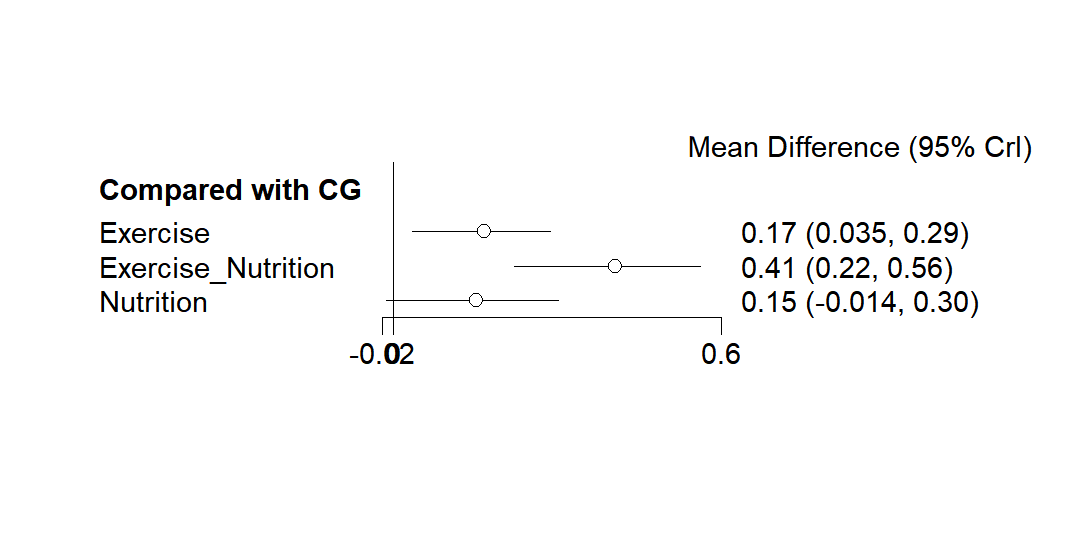


**11.1.3.3** Period
When the model was adjusted for centering value of period , the hierarchy from the unadjusted model retained.

**Figure S11.1.3.3** presents the impact of various interventions on overall symptom changes after adjusting for the period of 20.85.


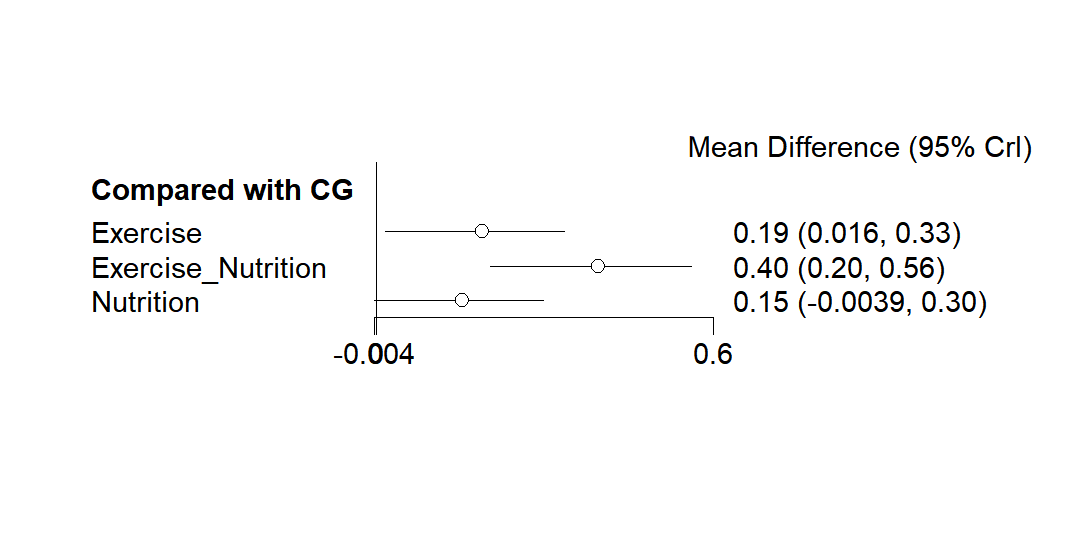


**11.1.3.4** Handgrip strength

When the model was adjusted for centering value of handgrip strength the hierarchy from the unadjusted model retained.

**Figure S11.1.3.4** presents the impact of exercise interventions on overall symptom changes after adjusting for the handgrip strength of 21.62.


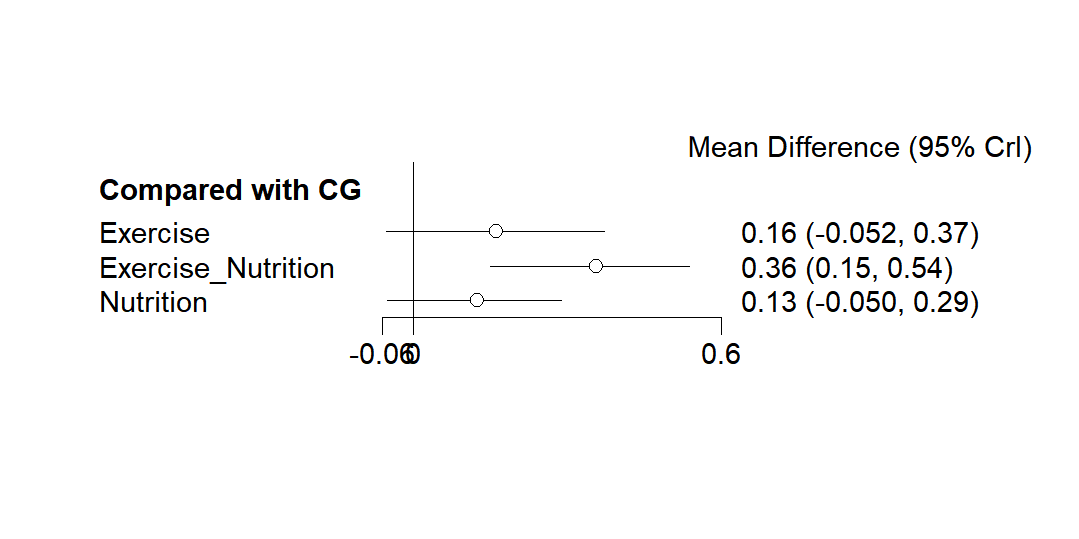


**11.1.3.5** BMI
When the model was adjusted for centering value of BMI, the hierarchy from the unadjusted model retained.

**Figure S11.1.3.5** presents the impact of various interventions on overall symptom changes after adjusting for BMI of 22.68.


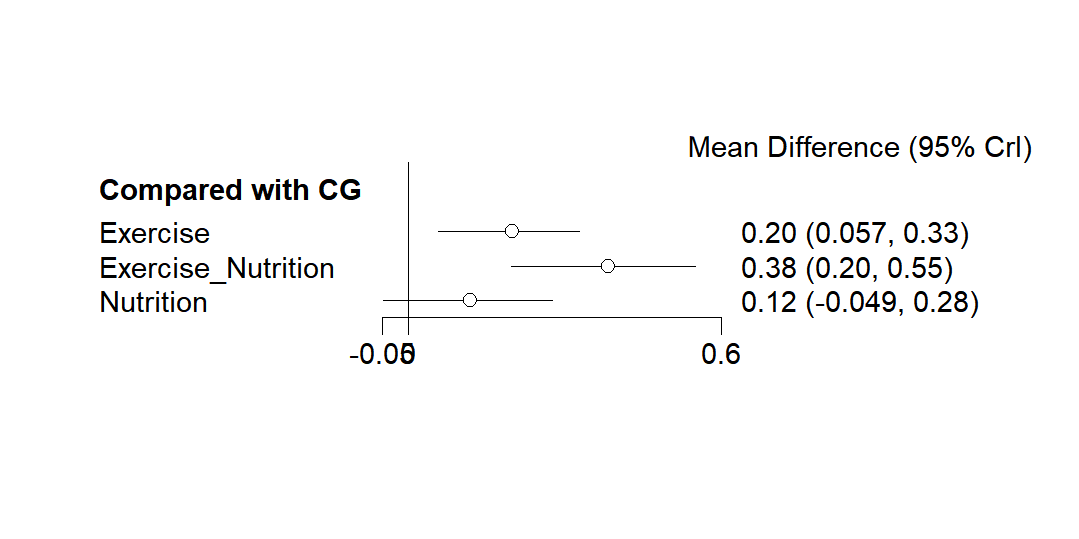


**11.1.3.6** Percentage of Male
When the model was adjusted for centering value of Percentage of Male, the hierarchy from the unadjusted model retained.

**Figure S11.1.3.6** presents the impact of various interventions on overall symptom changes after adjusting for the percentage of male of 48.74%.


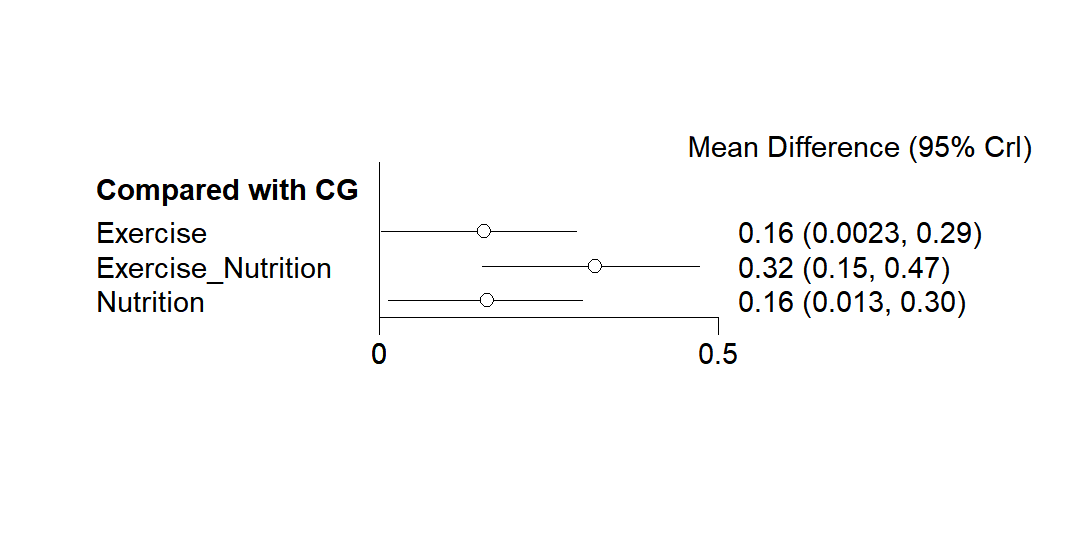


**11.1.3.7** ASMI
When the model was adjusted for centering value of ASMI, the hierarchy from the unadjusted model retained.

**Figure S11.1.3.7** presents the impact of various interventions on overall symptom changes after adjusting for the ASMI of 5.95.


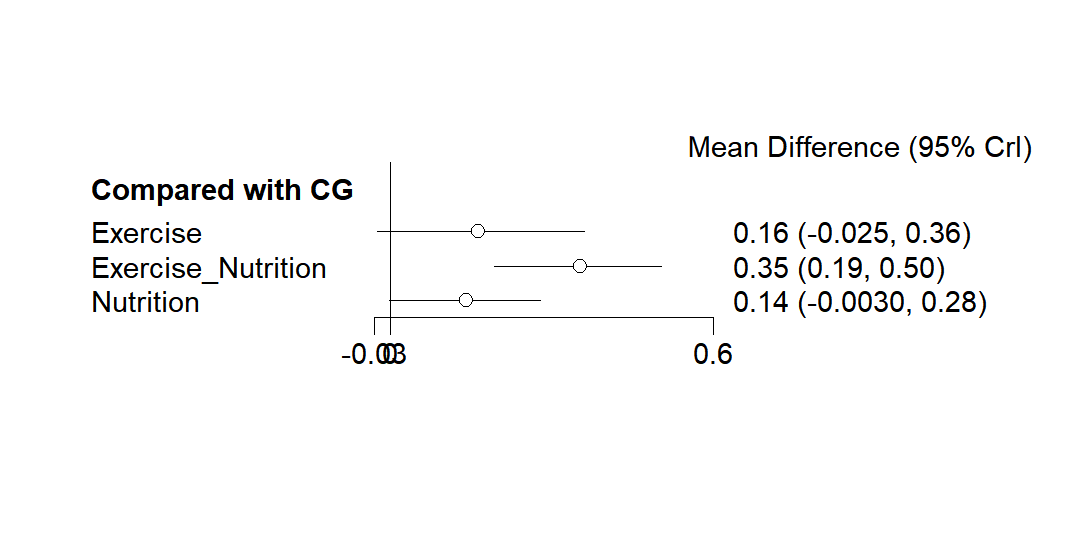


**Appendix 11.2** Risk-of-Bias-Based Sensitivity Analysis

Sensitivity analyses were undertaken by excluding studies classified as having a high risk of bias, in order to assess the robustness of the results.

**Figure S11.2.1** Forest plot of Handgrip strength


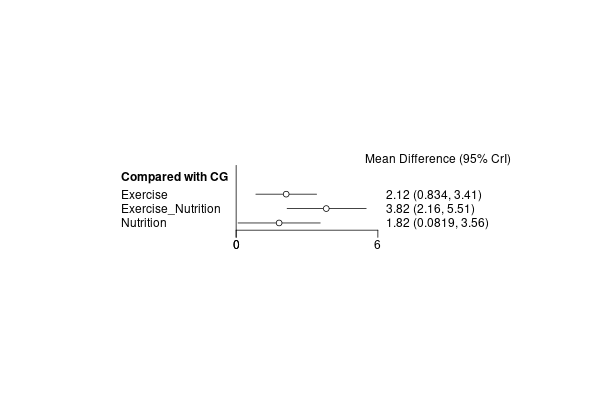


**Figure S11.2.2** Forest plot of Gait speed


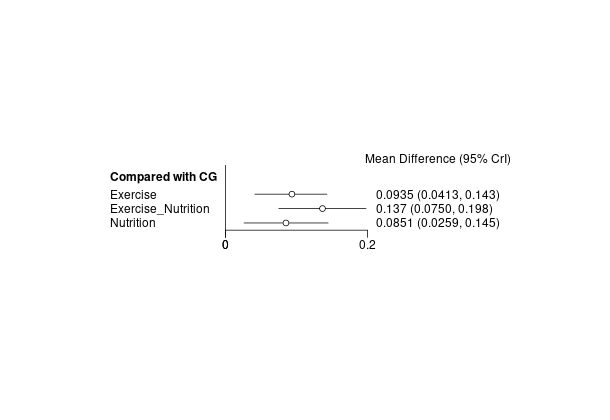


**Figure S11.2.3** Forest plot of SPPB


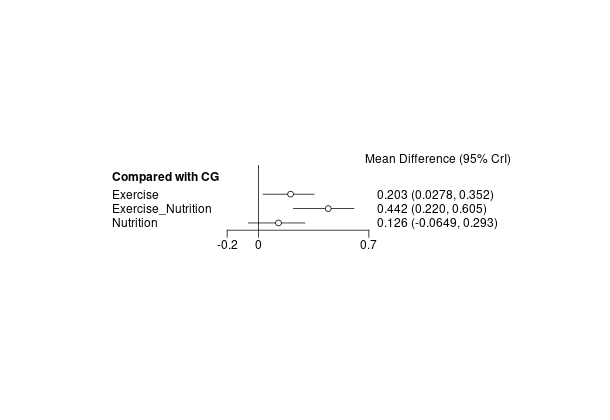

Supplement: Supplementary file 1 [file Table_1.DOCX]
